# Comparative Efficacy and Safety of Antidiabetic Drug Regimens Added to Metformin Monotherapy in Patients with Type 2 Diabetes: A Network Meta-Analysis

**DOI:** 10.1371/journal.pone.0125879

**Published:** 2015-04-28

**Authors:** Elizabeth S. Mearns, Diana M. Sobieraj, C. Michael White, Whitney J. Saulsberry, Christine G. Kohn, Yunes Doleh, Eric Zaccaro, Craig I. Coleman

**Affiliations:** 1 Department of Pharmacy Practice, School of Pharmacy, University of Connecticut, Storrs, Connecticut, United States of America; 2 Evidence-Based Practice Center, Hartford Hospital, Hartford, Connecticut, United States of America; 3 Department of Pharmacy Practice, School of Pharmacy, University of Saint Joseph, Hartford, Connecticut, United States of America; Providence VA Medical Center and Brown University, UNITED STATES

## Abstract

**Introduction:**

When first line therapy with metformin is insufficient for patients with type 2 diabetes (T2D), the optimal adjunctive therapy is unclear. We assessed the efficacy and safety of adjunctive antidiabetic agents in patients with inadequately controlled T2D on metformin alone.

**Materials and Methods:**

A search of MEDLINE and CENTRAL, clinicaltrials.gov, regulatory websites was performed. We included randomized controlled trials of 3–12 months duration, evaluating Food and Drug Administration or European Union approved agents (noninsulin and long acting, once daily basal insulins) in patients experiencing inadequate glycemic control with metformin monotherapy (≥1500 mg daily or maximally tolerated dose for ≥4 weeks). Random-effects network meta-analyses were used to compare the weighted mean difference for changes from baseline in HbA1c, body weight (BW) and systolic blood pressure (SBP), and the risk of developing hypoglycemia, urinary (UTI) and genital tract infection (GTI).

**Results:**

Sixty-two trials evaluating 25 agents were included. All agents significantly reduced HbA1c vs. placebo; albeit not to the same extent (range, 0.43% for miglitol to 1.29% for glibenclamide). Glargine, sulfonylureas (SUs) and nateglinide were associated with increased hypoglycemia risk vs. placebo (range, 4.00–11.67). Sodium glucose cotransporter-2 (SGLT2) inhibitors, glucagon-like peptide-1 analogs, miglitol and empagliflozin/linagliptin significantly reduced BW (range, 1.15–2.26kg) whereas SUs, thiazolindinediones, glargine and alogliptin/pioglitazone caused weight gain (range, 1.19–2.44kg). SGLT2 inhibitors, empagliflozin/linagliptin, liraglutide and sitagliptin decreased SBP (range, 1.88–5.43mmHg). No therapy increased UTI risk vs. placebo; however, SGLT2 inhibitors were associated with an increased risk of GTI (range, 2.16–8.03).

**Conclusions:**

Adding different AHAs to metformin was associated with varying effects on HbA1c, BW, SBP, hypoglycemia, UTI and GTI which should impact clinician choice when selecting adjunctive therapy.

## Introduction

The American Diabetes Association (ADA) and European Association for the Study of Diabetes (EASD) recommend lifestyle modifications and metformin as first-line therapy in type 2 diabetes mellitus (DM) [1]. However, initial monotherapy with maximally tolerated metformin may be insufficient to achieve hemoglobin A1c (HbA1c) goals of <7%, or given the progressive nature of Type 2 DM, glycemic control can wane over time necessitating combination therapy [1]. When monotherapy alone does not achieve/ maintain an HbA1c target over ~3 months, the next step is often to add a second agent. While there is an extensive list of pharmacologic therapies available for second-line adjunctive treatment of Type 2 DM (alpha-glucosidase inhibitors (AGIs), (dipeptidyl peptidase-4 (DPP-4) inhibitors, bile acid sequestrants, meglitinides, glucagon-like peptide-1 (GLP-1) analogs, long-acting, once-daily basal insulin, sodium glucose co-transporter-2 (SGLT2) inhibitors, sulfonylureas (SUs), thiazolidinediones (TZDs) and combinations of the above agents as either a fixed-dose combination or individual agents), randomized controlled trials (RCTs) directly comparing them are sparse.

Traditional pair-wise meta-analysis can be used to evaluate the efficacy and safety of two drugs based on evidence from RCTs that directly compare them. However, in absence of such direct head-to-head comparisons, network meta-analysis (NMA) provides a statistical framework that incorporates evidence from both direct and indirect comparisons from a network of studies of different therapies and evaluates their relative treatment effects [2–4].

We performed a NMA to assess the comparative efficacy and safety of adjunctive antidiabetic medication therapies in patients with Type 2 DM not adequately controlled on stable and optimized metformin monotherapy.

## Materials and Methods

### Study Selection

We performed a systematic literature search for all relevant articles from the earliest date through May 2014 in MEDLINE and Cochrane CENTRAL. The search strategy combined the Medical Subject Heading (MeSH) and keywords for “metformin” with terms for Type 2 DM and for glycosylated hemoglobin A1c (HbA1c). Our MEDLINE search strategy is included in S1 Appendix. We also performed a manual search of references from reports of clinical trials and review articles to identify additional relevant studies. Study results of identified studies were supplemented when possible with data identified through searches of www.clinicaltrials.gov, regulatory agency reports and by contacting investigators for clarification or additional data. Two investigators reviewed all potentially relevant citations independently (ESM, CIC).

To be included, studies had to: (1) be published in English; (2) utilize a parallel RCT design (any phase) in adults (≥18 years) with Type 2 DM; (3) compare Food and Drug Administration (FDA) or European Union (EU)-approved antidiabetic drug therapy including non-insulin and long-acting, once-daily basal insulin agents (as a single or combination adjunctive therapy) to another antidiabetic therapy or placebo (in addition to metformin); (4) include only patients who showed inadequate response to stable, optimized metformin monotherapy at randomization; (5) treat patients for 12 to 52 glycemic weeks after randomization; and (6) report change in HbA1c from baseline (our primary endpoint). As in previous NMAs [4], the criterion of stable metformin therapy was considered to be met if a study included patients who received at least 1,500mg/day (or maximum tolerated dose) of metformin or 1,000mg/day (as long as the mean dose in the study was >1,500mg/day) for at least the preceding 4 weeks before randomization.

### Validity Assessment

Validity assessment was performed by 2 investigators (ESM, DMS) independently using the Cochrane Risk of Bias Tool [2,5]. This checklist (S2 Appendix) includes 7 validity questions covering the following domains: random sequence generation, allocation concealment, blinding of participants and personnel, blinding of outcome assessment, incomplete data reporting, selective reporting, and other bias (i.e., participants were either not on stable, maximal or near-maximal metformin as monotherapy, received it for less than 12 weeks prior to randomization, and/or the study was not published in a peer-reviewed journal). Each item was scored as a low, unclear or high risk of bias.

### Data Extraction

Two investigators (ESM, DMS) used a standardized tool to independently extract all data with disagreements resolved by discussion or a third investigator (CIC). The following information was sought from each trial: (1) author identification; (2) year of publication; (3) study design and methodological quality information needed to complete the Cochrane Collaboration’s tool for assessing risk of bias [2,5]; (4) sample size; (5) inclusion/exclusion criteria; (6) duration of follow-up; (7) metformin and other antidiabetic therapies’, doses and schedules used; and (8) baseline characteristics (age, gender, anthropometrics, baseline HbA1c and duration of Type 2 DM). Endpoint data collected included mean change from baseline in HbA1c, body weight (BW), and systolic blood pressure (SBP) and the number/proportion of patients experiencing finger stick confirmed hypoglycemia, urinary tract infections (UTIs) and genital tract infections (GTIs). In cases where more than 1 published time point or article on an overlapping population was available, the most comprehensive article (e.g., the full analysis population) and primary endpoint time point (between 12 and 52 weeks) were used in the meta-analysis in order to optimize the amount of analyzable data.

### Statistical Analysis

We performed traditional meta-analyses analyzing changes in HbA1c, BW and SBP as continuous variables using StatsDirect version 2.7.8 (StatsDirect Ltd, Cheshire, UK) with a P<0.05 considered statistically significant. Separate pair-wise analyses were performed for each antidiabetic therapy, combining data from approved doses of the same therapies using the method recommended by the Cochrane Collaboration [2]. In all cases, weighted (absolute) mean differences (WMDs) and associated 95% confidence intervals (CIs) were calculated using a DerSimonian and Laird random-effects model to account for between study heterogeneity. Net changes in each of the endpoints were calculated as the difference between treatment groups in the changes (baseline to follow-up). In instances where variances for net changes were not reported directly, they were calculated from CIs, P-values or individual variances. If the standard error, standard deviation or 95%CIs) for paired differences were not reported, we calculated it from those at baseline and at the end of follow-up; assuming a correlation coefficient of 0.5 between initial and final values [2]. The proportion of patients experiencing confirmed hypoglycemia, UTI and GTI on each drug therapy (combining data from approved doses of the same therapies) was meta-analyzed using a random-effects model as dichotomous endpoints with weighted averages reported as relative risks (RRs) and associated 95%CIs. We included UTI and GTI as key adverse endpoints in this meta-analysis because they are particularly relevant to the SGLT-2 inhibitors, which are the newest second-line treatment option for patients with Type 2 DM. When at least 3 studies making the same direct comparison were available, the likelihood of statistical heterogeneity (using the I^2^ statistic with a value >50% representing important statistical heterogeneity) and publication bias (using the Egger’s weighted regression statistic with a P <0.05 suggesting a higher likelihood of publication bias) were assessed.

We then performed NMA, a generalization of traditional pairwise meta-analysis that compares all pairs of treatments within a set of treatments for the same disease state (in this case Type 2 DM) [3]. Along with analyzing direct within-trial comparisons between two treatments, the NMA framework enables incorporation of indirect comparisons constructed from two trials that have one treatment in common [3]. This type of analysis safeguards the within-trial randomized treatment comparison of each trial while combining all available comparisons between treatments; allowing for more precise estimates for compared interventions. We used the package ‘netmeta’ (version 0.5–0) in R (version 3.0.2, The R Foundation for Statistical Computing) to perform NMA [6]. The package uses a novel graph-theory methodology that exploits the analogy between treatment networks and electrical networks to construct a NMA model accounting for the correlated treatment effects in multi-arm trials [6]. We implemented a random-effects model assuming common heterogeneity across all comparisons. Inconsistency was assessed by statistically comparing the results from direct and indirect estimates, whenever indirect estimates could be calculated using a single common comparator. We ascribed clinical superiority if one therapy improved HbA1c, BW or SBP by at least 0.3% [7], 2.3 kg (5 pounds) [8] or 5 mmHg [9], respectively, versus a competitor and with 95% certainty (i.e., for one therapy to be clinically superior to another for change in HbA1c, BW or SBP the lower bound of the 95%CI must depict a reduction greater than 0.3%, 2.3 kg or 5 mmHg, respectively) (S1 Fig). To address the inherent limitations of multiple comparisons in our meta-analysis, we performed sensitivity analyses calculating more conservative 99%CIs.

## Results

### Study Characteristics

**Fig 1:**
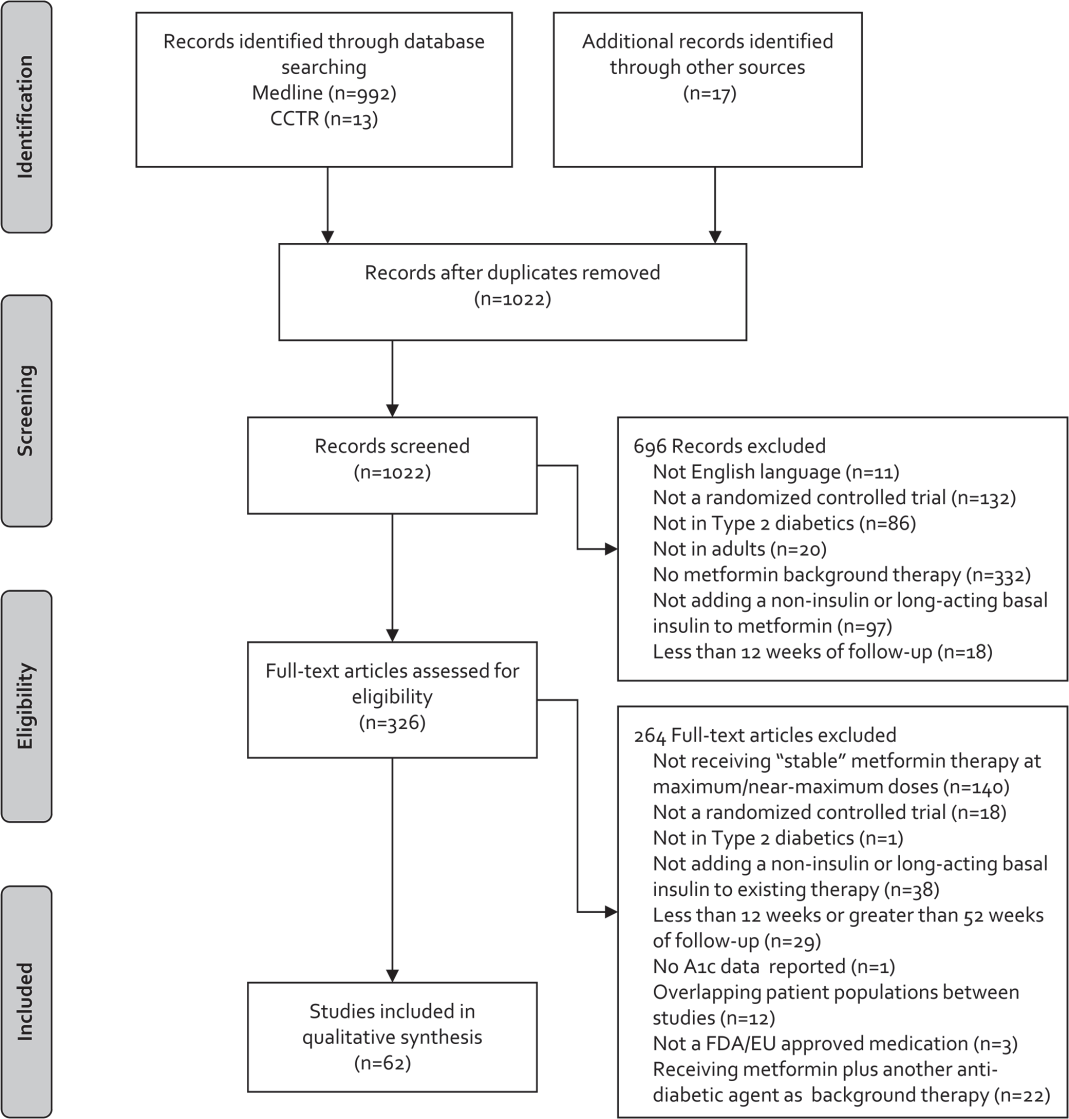
Results of the Literature Search. CCTR = Cochrane controlled trials register; EU = European Union; FDA = Food and Drug Administration; HbA1c = hemoglobin A1c.

The literature search yielded 1,005 citations, and we identified 17 additional RCTs through other sources (Fig 1). After removal of duplicates and title/abstract screening, 326 articles were eligible for full-text screening. After screening, 62 RCTs (n = 32,185 participants) were included in the NMA [10–71]. Twenty-five treatments, as well as placebo, were analyzed including AGIs (miglitol and acarbose), DPP-4 inhibitors (alogliptin, linagliptin, saxagliptin, sitagliptin and vildagliptin), the bile acid sequestrant (colesevelam), meglitinides (repaglinide and nateglinide), GLP-1 analogs (exenatide, lixisenatide and liraglutide), long-acting, once-daily basal insulin (insulin glargine), SGLT2 inhibitors (canagliflozin, dapagliflozin and empagliflozin), SUs (glibenclamide, gliclazide, glimepiride and glipizide), TZDs (rosiglitazone and pioglitazone), and combinations of the above agents as either a fixed-dose combination or individual agents (alogliptin/pioglitazone and empagliflozin/linagliptin).

**Fig 2:**
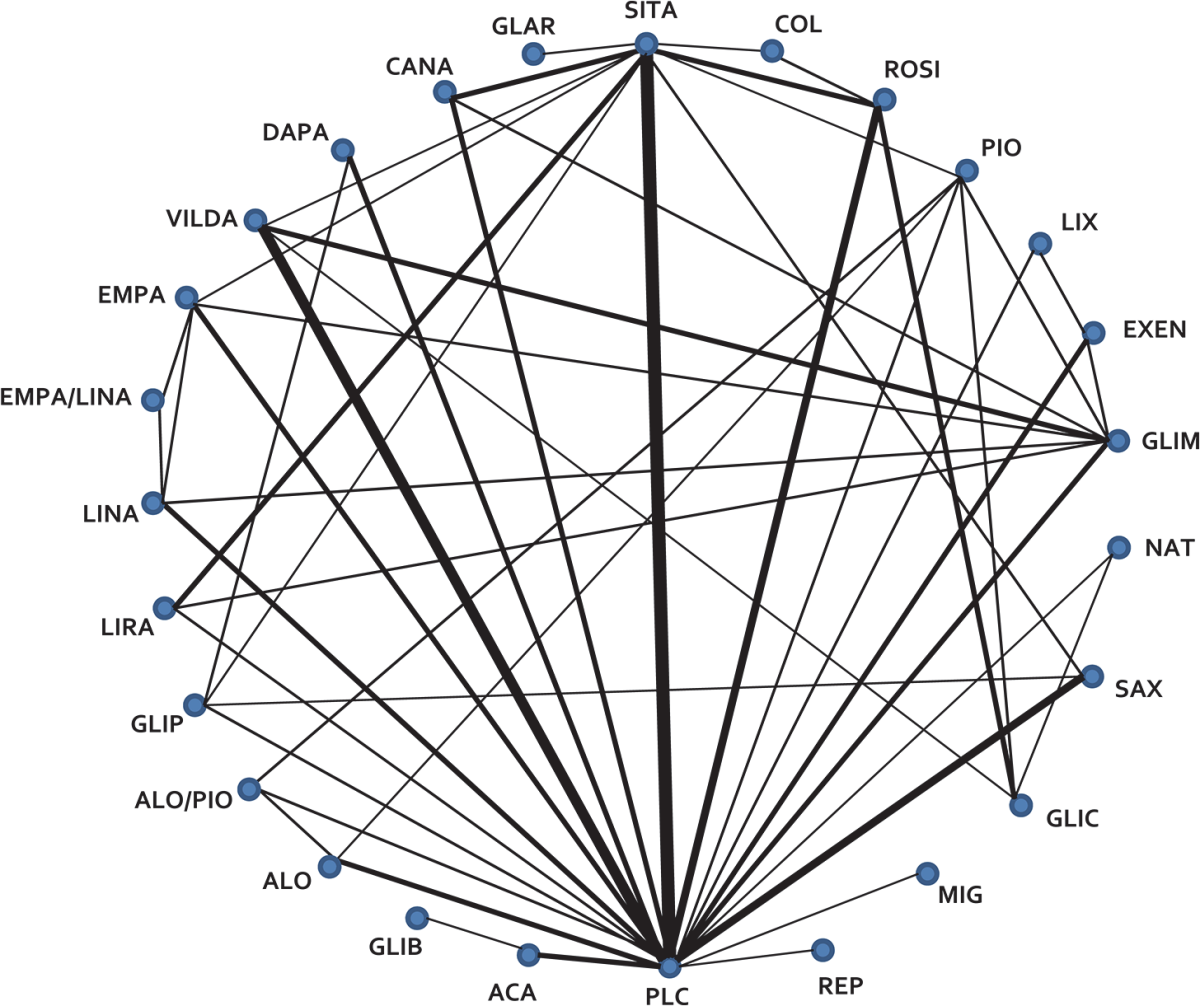
Network Diagram of Randomized Controlled Trials Evaluating Antidiabetic Therapies in Addition to Optimized Metformin in Type 2 Diabetes. All agents are in combination with optimized metformin and include oral and subcutaneous agents and long-acting, once daily basal insulins. Lines represent the presence of direct comparison trial(s). The width of the line is proportional to the number of trials with direct comparisons. ACA, acarbose; ALO, alogliptin; ALO/PIO, alogliptin/pioglitazone; CANA, canagliflozin; COL, colesevelam; DAPA, dapagliflozin; EMPA, empagliflozin; EMPA/LINA, empagliflozin/linagliptin; EXEN, exenatide; GLAR, insulin glargine; GLIB, glibenclamide; GLIC, gliclazide; GLIM, glimeperide; GLIP, glipizide; LINA, linagliptin; LIRA, liraglutide; LIX, lixisenatide; MIG, miglitol; NAT, nateglinide; PIO, pioglitizone; PLC, placebo; REP, repaglinide; ROSI, rosiglitizone; SAX, saxagliptin; SITA, sitagliptin; VILDA, vildagliptin

Characteristics of the included trials are described in S1 Table. The mean [range] trial duration was 29 [12–52] weeks; mean age ranged from 50–62 years; mean [range] BMI was 30.8 [25.0–34.6] kg/m2; mean [range] SBP was 131 [126–142] mmHg; and mean [range] baseline HbA1c was 8.0% [6.4–9.3%]. Fig 2 illustrates the network of RCTs with available direct comparisons, and individual RCT endpoint data are presented in Table 1. The overall quality of RCTs was rated as good to unclear with the majority of studies having few domains with a high risk of bias (S2 Fig).

**Table 1:** Outcomes Reported In Randomized Controlled Trials Evaluating Antidiabetic Therapies Added to Metformin in Adult Patients With Type 2 Diabetes.

Author, Year, N	Interventions Evaluated	Change in HbA1c, mean, % (SE)	Change in Body Weight, mean, kg (SE)	Change in Systolic Blood Pressure, mean, mmHg (SE)	Confirmed Hypoglycemia (n/N)	UTI (n/N)	GTI (n/N)
**Defronzo 2014, N = 686**	Empagliflozin 25 mg /Linagliptin 5 mg	-1.19 (0.06)	-3.0 (0.3)	-5.6 (1.0)	2/137	11/137	2/137
	Empagliflozin 10 mg /Linagliptin 5 mg	-1.08 (0.06)	-2.6 (0.3)	-4.1 (0.9)	2/136	6/136	6/136
	Empagliflozin 25 mg	-0.62 (0.06)	-3.2 (0.3)	-5.1 (0.9)	4/141	12/141	12/141
	Empagliflozin 10 mg	-0.66 (0.06)	-2.5 (0.3)	-4.0 (0.9)	2/140	9/140	7/140
	Linagliptin 5 mg	-0.70 (0.06)	-0.7 (0.3)	-1.0 (0.9)	2/132	16/132	1/132
**Bolli 2014, N = 484**	Lixisenatide 20 μg, 1-step titration	-0.9 (0.10)	-2.63 (0.4)	NR	3/161	NR	NR
	Lixisenatide 20 μg, 2-step titration	-0.8 (0.1)	-2.68 (0.4)	NR	4/161	NR	NR
	Placebo	-0.4 (0.1)	-1.63 (0.4)	NR	1/160	NR	NR
**Derosa 2014, N = 167**	Glimepiride 2 mg TID	-1.0 (0.07)	1.2 (0.74)	NR	NR	NR	NR
	Vildagliptin 50 mg BID	-1.0 (0.08)	-1.5 (0.70)	NR	NR	NR	NR
**Haring 2014, N = 637**	Empagliflozin 10 mg	-0.70 (0.05)	-2.08 (0.17)	-4.5 (0.7)	4/217	11/217	8/217
	Empagliflozin 25 mg	-0.77 (0.05)	-2.46 (0.17)	-5.2 (0.7)	3/213	12/213	10/213
	Placebo	-0.13 (0.05)	-0.45 (0.17)	-0.4 (0.7)	1/207	10/207	0/207
**Nauck 2014, N = 1098**	Sitagliptin 100 mg	-0.61 (0.05)	-1.49 (0.19)	-1.9 (0.7)	NR	11/315	NR
	Placebo	0.03 (0.08)	-1.59 (0.26)	1.1 (0.9)	NR	9/177	NR
**Ridderstråle 2014, N = 1549**	Empagliflozin 25 mg	-0.73 (0.03)	-3.2 (0.10)	-3.6 (0.43)	12/765	95/765	90/765
	Glimepiride 1–4 mg (mean dose = 2.7 mg/d)	-0.66 (0.03)	1.6 (0.10)	2.2 (0.46)	159/780	99/780	17/780
**White 2014, N = 160**	Saxagliptin 2.5 mg BID	-0.56 (0.09)	-0.32 (0.33)	1.59 (1.51)	0/74	NR	NR
	Placebo	-0.22 (0.08)	-0.40 (0.22)	0.83 (1.26)	1/86	NR	NR
**Charbonnel 2013, N = 650**	Sitagliptin 100 mg	-1.32 (0.05)	-0.4 (0.20)	0.9 (0.69)	39/326	NR	NR
	Liraglutide 1.2 mg	-1.42 (0.05)	-2.8 (0.23)	-1.9 (0.71)	13/324	NR	NR
**Chawla 2013, N = 52**	Sitagliptin 100 mg	-0.66 (0.04)	-0.58 (NR)	NR	NR	NR	NR
	Pioglitazone 30 mg	-0.75 (0.07)	0.90 (NR)	NR	NR	NR	NR
**Cefalu 2013, N = 1450**	Glimepiride 1–8 mg (mean dose = 5.6 mg/d)	-0.81 (0.04)	0.7 (0.2)	0.2 (0.6)	165/482	22/482	8/482
	Canagliflozin 100 mg	-0.82 (0.04)	-3.7 (0.2)	-3.3 (0.6)	27/483	31/483	43/483
	Canagliflozin 300 mg	-0.93 (0.04)	-4.0 (0.2)	-4.6 (0.6)	23/485	31/485	54/485
**Derosa 2013, N = 167**	Vildagliptin 50 mg BID	-1.2 (0.06)	-5.8 (0.6)	NR	0/84	NR	NR
	Placebo	-0.8 (0.07)	-5.1 (0.6)	NR	0/83	NR	NR
**Lavalle-González 2013, N = 1284**	Canagliflozin 100 mg	-0.79 (0.04)	-3.3 (0.2)	-3.8 (0.6)	10/368	18/368	20/368
	Canagliflozin 300 mg	-0.94 (0.04)	-3.6 (0.2)	-5.1 (0.6)	7/367	13/367	22/367
	Sitagliptin 100 mg	-0.82 0.04)	-1.1 (0.2)	-1.8 (0.6)	0/366	12/366	5/366
	Placebo	-0.17 (0.06)	-1.1 (0.2)	1.5 (0.8)	2/183	2/183	1/183
**Rosenstock 2013, N = 495**	Empagliflozin 10 mg	-0.56 (0.08)	-2.7 (0.33)	-4.39 (1.55)	0/71	3/71	7/71
	Empagliflozin 25 mg	-0.55 (0.08)	-2.6 (0.31)	-8.51 (1.53)	0/70	4/70	0/70
	Sitagliptin 100 mg	-0.45 (0.10)	-0.8 (0.33)	-1.79 (1.38)	2/71	3/71	2/71
	Placebo	0.15 (0.08)	-1.2 (0.33)	-2.23 (1.76)	0/71	2/71	0/71
**Rosenstock 2013, N = 634**	Lixisenatide 20 μg	-0.79 (0.05)	-2.96 (0.23)	NR	8/318	NR	NR
	Exenatide 10 μg BID	-0.96 (0.05)	-3.98 (0.23)	NR	25/316	NR	NR
**Aschner 2012, N = 515**	Insulin glargine w/titration	-1.72 (0.06)	0.44 (0.22)	NR	56/237	NR	NR
	Sitagliptin 100 mg	-1.13 (0.06)	-1.08 (0.2)	NR	12/264	NR	NR
**Bergenstal 2012, N = 666**	Sitagliptin 100 mg	-0.89 (0.06)	-0.9 (0.3)	NR	NR	NR	NR
	Placebo	-0.1 (0.08)	-0.5 (0.4)	NR	NR	NR	NR
**DeFronzo 2012, N = 1554**	Placebo	-0.13 (0.08)	-0.66 (0.29)	NR	NR	6/129	NR
	Alogliptin 12.5 mg	-0.64 (0.08)	-0.02 (0.29)	NR	NR	6/128	NR
	Alogliptin 25 mg	-0.90 (0.08)	-0.67 (0.29)	NR	NR	5/129	NR
	Pioglitazone 15 mg	-0.75 (0.08)	0.94 (0.29)	NR	NR	7/129	NR
	Pioglitazone 30 mg	-0.92 (0.08)	1.88 (0.29)	NR	NR	8/129	NR
	Pioglitazone 45 mg	-1.00 (0.08)	1.65 (0.29)	NR	NR	8/129	NR
	Alogliptin 12.5 mg + pioglitazone 15 mg	-1.34 (0.08)	1.25 (0.29)	NR	NR	5/130	NR
	Alogliptin 12.5 mg + pioglitazone 30 mg	-1.39 (0.08)	1.89 (0.29)	NR	NR	4/130	NR
	Alogliptin 12.5 mg + pioglitazone 45 mg	-1.55 (0.08)	2.30 (0.29)	NR	NR	5/130	NR
	Alogliptin 25 mg + pioglitazone 15 mg	-1.27 (0.08)	1.27 (0.29)	NR	NR	6/130	NR
	Alogliptin 25 mg + pioglitazone 30 mg	-1.39 (0.08)	2.10 (0.29)	NR	NR	4/130	NR
	Alogliptin 25 mg + pioglitazone 45 mg	-1.60 (0.08)	2.25 (0.29)	NR	NR	5/130	NR
**Derosa 2012, N = 171**	Exenatide 10 μg BID	-1.2 (0.02)	-6.4 (0.6)	NR	0/81	NR	NR
	Placebo	-0.4 (0.03)	-2.3 (1.0)	NR	0/82	NR	NR
**Derosa 2012, N = 178**	Sitagliptin 100 mg	-1.4 (0.01)	-2.5 (0.5)	NR	NR	NR	NR
	Placebo	-0.7 (0.02)	-2.3 (0.6)	NR	NR	NR	NR
**Gallwitz 2012, N = 1029**	Exenatide 10 μg BID	-0.66 (0.04)	NR	-3.1 (0.97)	165/511	NR	NR
	Glimepiride, 1mg—max tolerable dose (mean dose = 2.0 mg/d)	-0.48 (0.04)	NR	Referent	332/508	NR	NR
**Gallwitz 2012, N = 1551**	Linagliptin 5 mg	-0.36 (0.03)	-1.12 (0.13)	NR	41/776	50/776	NR
	Glimepiride 1–4 mg (mean dose = 2.7 mg/d)	-0.57 (0.03)	1.38 (0.14)	NR	249/775	52/775	NR
**Ljunggren 2012, N = 182**	Dapagliflozin 10 mg	-0.38 (0.06)	-4.39 (0.5)	NR	4/89	0/89	0/89
	Placebo	0.02 (0.06)	-2.03 (0.4)	NR	2/91	0/91	0/91
**Pan 2012, N = 438**	Vildagliptin 50 mg	-0.92 (0.08)	NR	NR	0/148	4/148	NR
	Vildagliptin 50 mg BID	-1.05 (0.08)	NR	NR	1/146	1/146	NR
	Placebo	-0.54 (0.08)	NR	NR	0/144	0/144	NR
**Rizzo 2012, N = 90**	Sitagliptin 100 mg	-1.2 (0.14)	NR	-1.0 (2.31)	NR	NR	NR
	Vildagliptin 50 mg BID	-1.0 (0.09)	NR	-3.0 (2.40)	NR	NR	NR
**Rosenstock 2012, N = 451**	Placebo	-0.22 (0.09)	-0.78 (0.3)	-1.3 (1.5)	1/65	4/65	1/65
	Canagliflozin 100 mg	-0.76 (0.13)	-2.25 (0.3)	1.0 (1.3)	1/64	2/64	4/64
	Canagliflozin 300 mg	-0.92 (0.09)	-2.88 (0.3)	-4.9 (1.5)	0/64	2/64	2/64
	Sitagliptin 100 mg	-0.74 (0.08)	-0.43 (0.3)	-0.8 (1.4)	3/65	1/65	1/65
**Ross 2012, N = 491**	Linagliptin 2.5 mg BID	-0.46 (0.05)	-0.4 (0.3)	NR	5/223	NR	NR
	Linagliptin 5 mg	-0.52 (0.05)	-1.0 (0.2)	NR	2/224	NR	NR
	Placebo	0.28 (0.11)	-1.1 (0.3)	NR	1/44	NR	NR
**Arechavaleta 2011, N = 1035**	Sitagliptin 100 mg	-0.47 (0.04)	-0.8 (0.15)	NR	36/516	NR	NR
	Glimepiride 1–6 mg (mean dose = 2.1 mg/d)	-0.54 (0.04)	1.2 (0.15)	NR	114/518	NR	NR
**Nauck 2011, N = 801**	Dapagliflozin 2.5–10 mg (mean dose = 9.2 mg/d)	-0.52 (0.04)	-3.22 (0.18)	-5.0 (0.84)	7/406	30/406	50/406
	Glipizide 5–20 mg (mean dose = 16.4 mg/d)	-0.52 (0.04)	1.44 (0.18)	Referent	147/408	17/408	11/408
**Pfützner 2011, N = 288**	Pioglitazone 15 mg BID	-0.83 (0.07)	0.7 (0.36)	-2.5 (1.23)	2/146	NR	NR
	Glimepiride 1 mg BID	-0.95 (0.07)	0.7 (0.36)	0.5 (1.55)	5/142	NR	NR
**Taskinen 2011, N = 700**	Placebo	0.15 (0.06)	-0.5 (NR)	NR	5/177	7/177	NR
	Linagliptin 5 mg	-0.49 (0.04)	-0.4 (NR)	NR	3/523	16/523	NR
**Wang 2011, N = 55**	Acarbose 100 mg TID	-0.7 (0.15)	-1.5 (0.4)	NR	0/28	NR	NR
	Glibenclamide 5 mg TID	-1.2 (0.30)	0.8 (0.5)	NR	NR	NR	NR
**Yang 2011, N = 570**	Saxagliptin 5 mg	-0.78 (0.05)	-1.05 (0.1)	-5.22 (NR)	0/283	13/283	NR
	Placebo	-0.37 (0.05)	-0.97 (0.1)	-3.10 (NR)	0/287	8/287	NR
**Bailey 2010, N = 546**	Placebo	-0.30 (0.07)	-0.9 (0.3)	-0.2 (1.2)	4/137	11/137	7/137
	Dapagliflozin 5 mg	-0.70 (0.07)	-3.0 (0.2)	-4.3 (1.3)	5/137	10/137	18/137
	Dapagliflozin 10 mg	-0.84 (0.07)	-2.9 (0.2)	-5.1 (1.3)	5/135	11/135	12/135
**Filozof 2010, N = 1007**	Vildagliptin 50 mg BID	-0.81 (0.06)	-1.28 (0.39)	NR	NR	NR	NR
	Gliclazide 80–320 mg (mean dose = 230 mg/d)	-0.85 (0.06)	Referent	NR	NR	NR	NR
**Goke 2010, N = 858**	Saxagliptin 5 mg	-0.74 (0.05)	-1.1 (0.19)	-4.1 (NR)	0/428	NR	NR
	Glipizide 5–20 mg (mean dose = 14.7 mg/d)	-0.80 (0.04)	1.1 (0.16)	-1.2 (NR)	38/430	NR	NR
**Pratley 2010, N = 665**	Liraglutide 1.2 mg	-1.24 (0.07)	-2.86 (0.3)	-0.55 (0.9)	12/221	NR	NR
	Liraglutide 1.8 mg	-1.50 (0.07)	-3.38 (0.3)	-0.72 (0.9)	11/218	NR	NR
	Sitagliptin 100 mg	-0.90 (0.07)	-0.96 (0.3)	-0.94 (0.9)	10/219	NR	NR
**Rigby 2010, N = 169**	Colesevelam 3.75 g	-0.3 (0.13)	-0.64 (0.3)	NR	NR	NR	NR
	Rosiglitazone 4 mg	-0.6 (0.13)	0.26 (0.5)	NR	NR	NR	NR
	Sitagliptin 100 mg	-0.4 (0.13)	-1.15 (0.3)	NR	NR	NR	NR
**Scheen 2010, N = 801**	Saxagliptin 5 mg	-0.52 (0.04)	-0.4 (NR)	NR	13/406	23/403	NR
	Sitagliptin 100 mg	-0.62 (0.04)	-0.4 (NR)	NR	11/393	21/398	NR
**DeFronzo 2009, N = 743**	Placebo	0.13 (0.07)	-0.92 (NR)	-4.5 (1.3)	1/179	8/179	NR
	Saxagliptin 2.5 mg	-0.59 (0.07)	-1.43 (NR)	-4.3 (1.2)	1/192	10/192	NR
	Saxagliptin 5 mg	-0.69 (0.07)	-0.87 (NR)	-3.6 (1.2)	1/191	10/191	NR
**Ferrannini 2009, N = 2789**	Vildagliptin 50mg BID	-0.44 (0.02)	-0.23 (0.11)	NR	23/1389	NR	NR
	Glimepiride 2–6 mg(mean dose = 4.5 mg/d)	-0.53 (0.02)	1.56 (0.12)	NR	224/1383	NR	NR
**Goodman 2009, N = 370**	Vildagliptin 100mg	-0.60 (0.08)	0.75 (0.31)	NR	2/248	NR	NR
	Placebo	0.17 (0.11)	Referent	NR	0/122	NR	NR
**Nauck 2009, N = 527**	Placebo	-0.1 (0.1)	-0.39 (0.27)	NR	3/104	4/104	NR
	Alogliptin 12.5 mg	-0.6 (0.1)	-0.39 (0.19)	NR	2/213	14/213	NR
	Alogliptin 25 mg	-0.6 (0.1)	-0.67 (0.20)	NR	0/210	6/210	NR
**Nauck 2009, N = 1087**	Liraglutide 0.6 mg	-0.7 (0.1)	-1.8 (0.2)	NR	7/242	NR	NR
	Liraglutide 1.2 mg	-1.0 (0.1)	-2.6 (0.2)	-3.2 (1.29)	7/240	NR	NR
	Liraglutide 1.8 mg	-1.0 (0.1)	-2.8 (0.2)	-2.7 (1.36)	7/242	NR	NR
	Glimepiride 4 mg	-1.0 (0.1)	1.0 (0.2)	Referent	41/242	NR	NR
	Placebo	0.1 (0.1)	-1.5 (0.3)	NR	4/121	NR	NR
**Hamann 2008, N = 596**	Rosiglitazone 4 mg	-0.78 (0.06)	2.7 (0.3)	-2.6 (1.38)^a^	1/294	7/294^a^	NR
	Gliclazide 80–320mg(mean dose = 238.1 mg/d)	-0.86 (0.06)	1.6 (0.3)	Referent	21/301	9/301	NR
**Khanolkar 2008, N = 50**	Rosiglitazone 4 mg	-1.19 (0.11)	NR	NR	NR	NR	NR
	Gliclazide 80 mg	-1.0 (0.13)	NR	NR	NR	NR	NR
**Raz 2008, N = 190**	Sitagliptin 100 mg	-0.98 (0.14)	-0.5 (NR)	NR	1/96	4/96	NR
	Placebo	0.04 (0.14)	-0.5 (NR)	NR	0/94	3/94	NR
**Scott 2008, N = 273**	Placebo	-0.22 (0.07)	-0.8 (0.2)	NR	2/91	NR	NR
	Sitagliptin 100 mg	-0.73 (0.07)	-0.4 (0.2)	NR	1/94	NR	NR
	Rosiglitazone 8 mg	-0.79 (0.07)	1.5 (0.2)	NR	1/87	NR	NR
**Bosi 2007, N = 544**	Vildagliptin 50 mg	-0.5 (0.1)	-0.4 (0.3)	NR	1/143	NR	NR
	Vildagliptin 100 mg	-0.9 (0.1)	0.2 (0.3)	NR	1/143	NR	NR
	Placebo	0.2 (0.1)	1.0 (0.3)	NR	1/130	NR	NR
**Nauck 2007, N = 1172**	Sitagliptin 100 mg	-0.67 (0.04)	-1.5 (0.28)	NR	29/588	44/588	NR
	Glipizide 5–20 mg (mean dose = 10.3 mg/d)	-0.67 (0.04)	1.1 (0.28)	NR	187/584	25/584	NR
**Ristic 2006, N = 262**	Nateglinide 60–180 mg TID (59.4% of patients on 540 mg/d)	-0.41 (0.08)	NR	NR	28/130	NR	NR
	Gliclazide 80–240 mg (35.7% of patients on 240 mg/d)	-0.57 (0.08)	NR	NR	28/126	NR	NR
**DeFronzo 2005, N = 336**	Placebo	0.1 (0.1)	-0.3 (0.3)	NR	6/113	NR	NR
	Exenatide 5 μg BID	-0.4 (0.1)	-1.6 (0.4)	NR	5/110	NR	NR
	Exenatide 10 μg BID	-0.8 (0.1)	-2.8 (0.5)	NR	6/113	NR	NR
**Feinglos 2005, N = 122**	Glipizide 2.5 mg	-0.66 (0.1)	2.1 (0.64)	NR	9/61	NR	NR
	Placebo	-0.19 (0.1)	Referent	NR	2/61	NR	NR
**Matthews 2005, N = 630**	Pioglitazone 15–45 mg (mean dose = 39 mg/d)	0.02 (0.09)	1.5 (NR)	NR	4/317	NR	NR
	Gliclazide 80–320 mg (mean dose = 212 mg/d)	Referent	1.4 (NR)	NR	35/313	NR	NR
**Ahren 2004, N = 71**	Vildagliptin 50 mg	-0.6 (0.1)	-0.4 (0.2)	NR	2/56	1/56	NR
	Placebo	0.1 (0.1)	-0.5 (0.2)	NR	0/51	3/51	NR
**Gomez-Perez 2002, N = 116**	Placebo	0.3 (0.3)	-0.86 (0.5)	NR	0/34	NR	NR
	Rosiglitazone 2 mg BID	-0.7 (0.2)	0.26 (0.6)	NR	0/35	NR	NR
	Rosiglitazone 4 mg BID	-1.2 (0.3)	2.42 (0.6)	NR	0/36	NR	NR
**Marre 2002, N = 467**	Nateglinide 60 mg TID	-0.36 (0.12)	0.4 (0.2)	NR	0/155	NR	NR
	Nateglinide 120 mg TID	-0.51 (0.12)	1.0 (0.2)	NR	5/160	NR	NR
	Placebo	Referent	0.1 (0.2)	NR	1/152	NR	NR
**Charpentier 2001, N = 372**	Placebo	0.07 (0.14)	-0.74 (0.3)	-0.65 (1.41)	11/75	NR	NR
	Glimepiride 1–6 mg (41% of patients on 6 mg/d)	-0.74 (0.08)	0.6 (0.2)	-0.14 (1.09)	22/147	NR	NR
**Van Gaal 2001, N = 152**	Miglitol 100 mg TID	-0.21 (0.1)	-2.5 (0.4)	NR	0/78	NR	NR
	Placebo	0.22 (0.1)	-0.7 (0.3)	NR	0/75	NR	NR
**Halimi 2000, N = 129**	Acarbose 50–100 mg TID	-0.7 (0.16)	NR	NR	NR	NR	NR
	Placebo	0.2 (0.16)	NR	NR	NR	NR	NR
**Fonseca 2000, N = 348**	Placebo	0.45 (0.12)	Referent	NR	2/116	NR	NR
	Rosiglitazone 4 mg	-0.56 (0.11)	1.9 (0.49)	NR	3/119	NR	NR
	Rosiglitazone 8 mg	-0.78 (0.19)	3.1 (0.80)	NR	5/113	NR	NR
**Moses 1999, N = 83**	Repaglinide 4 mg	-1.41 (0.23)	2.41 (0.5)	NR	9/27	NR	NR
	Placebo	-0.33 (0.24)	-0.86 (0.51)	NR	0/27	NR	NR
**Rosenstock 1998, N = 148**	Acarbose 50–100 mg TID	-0.71 (0.18)	-0.10 (0.42)	NR	1/73	NR	NR
	Placebo	Referent	Referent	NR	2/74	NR	NR

BMI = body mass index; DM = diabetes mellitus; HbA1c = hemoglobin A1c; n = number of patients; NR = not reported; SE = standard error

^a^ Not included in network meta-analysis because neither rosiglitazone nor gliclazide are connected to the network of trials.

### Change in HbA1c

#### SGLT2 Inhibitors

The SGLT2 inhibitors had similar effects on reducing HbA1c when compared to placebo in the NMA, ranging from 0.48% for dapagliflozin to 0.72% for canagliflozin (S3 Fig). When compared to the other active single agents, canagliflozin was associated with statistically significant reductions in HbA1c compared with dapagliflozin, nateglinide and saxagliptin; and empagliflozin was significantly more efficacious compared to dapagliflozin and saxagliptin. Dapagliflozin was inferior in reducing HbA1c when compared to 11 (50%) of the other active single agents. Along with statistical significance, all SGLT-2 inhibitors were found to be clinically superior to placebo (lower bound of the 95%CI depicted an HbA1c reduction greater than 0.3%), however none of the SGLT2 inhibitors were clinically superior in reducing HbA1c to any other active agents analyzed.

#### Combination Agents

Both combination agents were associated with significant reductions in HbA1c when compared to placebo in the NMA (alogliptin/pioglitazone: 1.24, 95% CI: 1.02–1.45%; empagliflozin/linagliptin: 1.13%, 95% CI: 0.92–1.34%). When comparing the combination medications to the other active single agents, alogliptin/pioglitazone significantly reduced HbA1c when compared to all other therapies except for insulin glargine, glibenclamide and repaglinide; whereas empagliflozin/linagliptin was more efficacious when compared to all other active single agents except for insulin glargine, glibenclamide, repaglinide and acarbose.

In terms of clinical superiority (lower bound of the 95%CI depicted an HbA1c reduction greater than 0.3%) alogliptin/pioglitazone and empagliflozin/linagliptin were clinically superior to 52% and 24% of the other antidiabetic medications analyzed, respectively. Alogliptin/pioglitazone was clinically superior to all DPP-4 inhibitors, colesevelam, dapagliflozin, glipizide, lixisenatide, miglitol, nataglinide, empagliflozin and pioglitazone. Empagliflozin/linagliptin was clinically superior to canagliflozin, dapagliflozin, glipizide, miglitol, nateglinide and saxagliptin.

#### All Other Agents

All antidiabetic agents were associated with statistically significant reductions in HbA1c relative to placebo, ranging from 0.43% for miglitol to 1.29% for glibenclamide (Table 2). When comparing single active agents, insulin glargine was associated with statistically significant reductions in HbA1c compared with all other therapies, except glibenclamide and repaglinide. Exenatide showed significant reductions in HbA1c when compared to the DPP-4 inhibitors, lixisenatide, miglitol, nateglinide, glipizide and dapagliflozin.

**Table 2:** Results of Network Meta-Analysis and Traditional Pair-Wise Meta-Analysis Comparing Antidiabetic Therapies’ Effect on Change in HbA1c, Body Weight and Systolic Blood pressure.

	Change in HbA1c, %			Change in Body Weight, kg			Change in Systolic Blood Pressure, mmHg		
**Comparison vs. Placebo**	**No. of Trials** ^**a**^	**Direct Estimate, WMD (95%CI)**	**NMA Estimate, WMD (95%CI)**	**No. of Trials** ^**a**^	**Direct Estimate, WMD (95%CI)**	**NMA Estimate, WMD (95%CI)**	**No. of Trials** ^**a**^	**Direct Estimate, WMD (95%CI)**	**NMA Estimate, WMD (95%CI)**
**Acarbose**	2	-0.78 (-1.06, -0.50)	-0.79 (-1.09,-0.48)	1	-0.10 (-0.92, 0.72)	-0.1 (-1.15, 0.95)	0	—	—
**Alogliptin**	2	-0.60 (-0.76, -0.43)	-0.57 (-0.76,-0.38)	2	0.05 (-0.41, 0.51)	0.09 (-0.53, 0.72)	0	—	—
**Alogliptin/pioglitazone**	1	-1.29 (-1.49, -1.09)	-1.24 (-1.45,-1.02)	1	2.50 (1.74, 3.26)	2.39 (1.55, 3.23)	0	—	—
**Canagliflozin**	2	-0.67 (-0.79, -0.55)	-0.72 (-0.85,-0.59)	2	-2.10 (-2.65, -1.55)	-2.15 (-2.63, -1.67)	2	-3.52 (-8.74, 1.70)	-4.14 (-5.8, -2.48)
**Colesevelam**	0	—	-0.5 (-0.85,-0.14)	0	—	0.89 (-0.11, 1.9)	0	—	—
**Dapagliflozin**	2	-0.43 (-0.55, -0.31)	-0.48 (-0.62,-0.33)	2	-2.10 (-2.62, -1.59)	-2.17 (-2.78, -1.57)	1	-4.5 (-7.54, -1.46)	-4.5 (-7.97, -1.03)
**Empagliflozin**	2	-0.64 (-0.75, -0.52)	-0.69 (-0.81,-0.57)	2	-1.74 (-2.10, -1.37)	-2.08 (-2.52, -1.63)	2	-4.41 (-5.96, -2.87)	-5.14 (-6.8, -3.48)
**Emapgliflozin/linagliptin**	0	—	-1.13 (-1.34,-0.92)	0	—	-2.07 (-2.95, -1.19)	0	—	-5.43 (-8.39, -2.47)
**Exenatide**	2	-0.79 (-0.86, -0.72)	-0.8 (-0.92,-0.67)	2	-2.76 (-4.85, -0.67)	-2.26 (-3.15, -1.37)	0	—	-2.84 (-5.89, 0.22)
**Insulin glargine**	0	—	-1.23 (-1.49,-0.97)	0	—	1.73 (0.8, 2.66)	0	—	—
**Glibenclamide**	0	—	-1.29 (-2.01,-0.56)	0	—	2.2 (0.45, 3.95)	0	—	—
**Gliclazide**	0	—	-0.7 (-0.85,-0.56)	0	—	1.19 (0.39, 1.99)	0	—	—
**Glimepiride**	2	-0.95 (-1.23, -0.67)	-0.73 (-0.82,-0.64)	2	1.92 (0.78, 3.06)	2.19 (1.84, 2.54)	1	0.51 (-3.08, 4.10)	0.26 (-1.44, 1.96)
**Glipizide**	1	-0.47 (-0.74, -0.20)	-0.55 (-0.68,-0.42)	1	2.10 (0.85, 3.35)	2.44 (1.88, 3)	0	—	0.5 (-3.69, 4.69)
**Linagliptin**	2	-0.68 (-0.81, -0.54)	-0.64 (-0.77,-0.52)	1	0.40 (-0.50, 1.30)	-0.04 (-0.62, 0.54)	0	—	-1.58 (-4.81, 1.64)
**Liraglutide**	1	-1.00 (-1.26, -0.75)	-0.86 (-1.0,-0.71)	1	-0.90 (-1.49, -0.31)	-1.6 (-2.12, -1.08)	0	—	-3.04 (-5.05, -1.03)
**Lixisenatide**	1	-0.45 (-0.69, -0.22)	-0.56 (-0.76,-0.37)	1	-1.02 (-1.96, -0.08)	-1.15 (-2.05, -0.26)	0	—	—
**Miglitol**	1	-0.43 (-0.70, -0.16)	-0.43 (-0.76,-0.1)	1	-1.80 (-2.78, -0.82)	-1.8 (-2.98,-0.62)	0	—	—
**Nateglinide**	1	-0.44 (-0.60, -0.28)	-0.48 (-0.67,-0.29)	1	0.60 (0.13, 1.07)	0.6 (-0.2, 1.4)	0	—	—
**Pioglitazone**	1	-0.76 (-0.98, -0.54)	-0.69 (-0.83,-0.55)	1	2.14 (1.32, 2.96)	2.06 (1.31, 2.81)	0	—	-2.74 (-6.82, 1.35)
**Repaglinide**	1	-1.08 (-1.73, -0.43)	-1.08 (-1.75,-0.41)	1	3.27 (1.88, 4.66)	3.27 (1.73, 4.81)	0	—	—
**Rosiglitazone**	3	-0.91 (-1.38, -0.44) ^b^	-0.75 (-0.9,-0.6)	3	2.27 (1.84, 2.70)	2.15 (1.65, 2.66)	0	—	—
**Saxagliptin**	3	-0.50 (-0.74, -0.26) ^b^	-0.51 (-0.62,-0.39)	2	-0.06 (-0.32, 0.20)	0.06 (-0.45, 0.57)	2	0.64 (-1.86, 3.13)	0.64 (-2.13, 3.41)
**Sitagliptin**	8	-0.67 (-0.73, -0.60)	-0.64 (-0.71,-0.57)	7	0.16 (-0.12, 0.45)	0.21 (-0.1, 0.53)	4	-2.11 (-3.90, -0.32)^c^	-1.88 (-3.38, -0.38)
**Vildagliptin**	5	-0.63 (-0.83, -0.43) ^b^	-0.63 (-0.72,-0.53)	4	-0.16 (-1.02, 0.70)^b^	0.04 (-0.37, 0.44)	0	—	-3.88 (-10.79, 3.02)

CI = confidence interval; HbA1c = hemoglobin A1c; NMA = network meta-analysis; WMD = weighted mean difference

^a^ Number of trials with direct comparisons

^b^ I^2^>50%

^c^ Egger’s p-value <0.5.

Along with statistical significance, all therapies were found clinically superior to placebo (lower bound of the 95%CI depicted an HbA1c reduction greater than 0.3%) except for colesevelam, nateglinide and miglitol (lower limit of 95%CI = -0.14, -0.28 and -0.16, respectively). In head-to-head comparisons, and insulin glargine was clinically superior to 40% of the other antidiabetic medications analyzed. Insulin glargine was found to be clinically superior to all DPP-4 inhibitors, colesevelam, dapagliflozin, glipizide, lixisenatide, miglitol, and nataglinide.

### Body Weight

#### SGLT2 Inhibitors

All SGLT2 inhibitors were associated with significant weight loss when compared to placebo in the NMA (range: 2.08–2.17 kg) (S4 Fig). When comparing active drugs, SGLT2 inhibitors were associated with statistically greater weight loss compared to all other agents analyzed except GLP-1 analogs, empagliflozin/linagliptin and miglitol. Moreover, SGLT2 inhibitors were associated with both statistically significant and clinically superior weight loss compared to SUs, TZDs and insulin glargine (range: 3.81–4.61 kg).

#### Combination Agents

Alogliptin/pioglitazone was associated with significant increases in BW when compared with placebo in the NMA (2.39 kg, 95% CI: 1.55–3.23 kg) whereas empagliflozin/linagliptin was associated with significant weight loss (2.07 kg, 95% CI: 1.19–2.95 kg). When compared to other single active agents, empagliflozin/linagliptin was associated with significant weight loss compared to all other agents except SGLT-2 inhibitors, and GLP-1 analogs. Alogliptin/pioglitazone caused significant increases in BW compared to all other active agents except SUs, repaglinide and TZDs. In terms of clinically superior weight gain, (lower bound of the 95%CI depicted a decrease in weight less than 2.3 kg), alogliptin/pioglitazone was associated with clinically superior weight gain compared to SGLT2 inhibitors, empagliflozin/linagliptin, GLP-1 analogs, and miglitol (range: 3.54–4.65 kg).

#### All Other Agents

The SUs, TZDs, insulin glargine and repaglinide were associated with significant increases in BW when compared with placebo in the NMA (range: 1.19–2.44 kg). GLP-1 analogs and miglitol were associated with significant weight loss (range: 1.15–2.26 kg) but there was no weight change with acarbose, any DPP-4 inhibitor, colesevelam and nateglinide when compared to placebo. When comparing active agents, GLP-1 analogs were associated with statistically greater weight loss when compared to all other agents except SGLT2 inhibitors and miglitol. While several agents exhibited statistically significant weight loss, no agent demonstrated clinically superior weight loss compared to placebo (lower bound of the 95%CI depicted a decrease in weight less than 2.3 kg). When comparing the clinical superiority of single active agents, TZDs were associated with clinically superior weight gain when compared to GLP-1 analogs (range: 3.22–4.41 kg).

### Systolic Blood Pressure

#### SGLT2 Inhibitors

All SGLT2 inhibitors were associated with a decrease in SBP compared with placebo in the NMA (range: 4.14–5.14 mmHg. When comparing active agents, SGLT2 inhibitors significantly reduced SBP when compared to the SUs (glimepiride, glipizide) (range: 4.4–5.64 mmHg), and saxagliptin and sitagliptin (range: 2.26–5.79 mmHg) (S5 Fig). No SGLT2 inhibitor showed clinical superiority (lower bound of the 95%CIs depicted a decrease in SBP less than 5 mmHg) compared to placebo or another active agent.

#### Combination Agents

Empagliflozin/linagliptin was associated with a decrease in SBP when compared with placebo in the NMA (5.43 mmHg, 95% CI: 2.47–8.39 mmHg). In head-to-head comparisons, empagliflozin/linagliptin significantly reduced SBP when compared to SUs, linagliptin, saxagliptin and sitagliptin; however it did not show clinical superiority compared to any other active agents. There were no data to evaluate alogliptin/pioglitazone for this endpoint.

#### All Other Agents

Liraglutide (3.04 mmHg, 95% CI: 1.03–5.05 mmHg) and sitagliptin (1.88 mmHg, 95% CI: 0.38–3.38 mmHg) were associated with a decrease in SBP compared with placebo. No medication showed clinical superiority (lower bound of the 95%CIs depicted a decrease in SBP less than 5 mmHg) compared to placebo or another active agent; however, there were no data to evaluate 12 (48%) of the agents for this endpoint.

### Confirmed Hypoglycemia

#### SGLT2 Inhibitors

Upon NMA, the SGLT2 inhibitors were not associated with an increased risk of confirmed hypoglycemia compared with placebo (S6 Fig). In the active drug comparisons, insulin glargine, nateglinide and all SUs were associated with significantly higher rates of confirmed hypoglycemia compared to any SGLT2 inhibitor (RR range, 4.14–22.93).

#### Combination Agents

Empagliflozin/linagliptin was not associated with increased risk of hypoglycemia compared with placebo in the NMA (0.38, 95% CI: 0.06–2.34). In the active drug comparisons, insulin glargine, nateglinide, both meglitinides and all SUs were associated with significantly higher rates of confirmed hypoglycemia compared to empagliflozin/linagliptin (RR range, 10.54–49.88). There were no data to evaluate alogliptin/pioglitazone for this endpoint.

#### All Other Agents

Insulin glargine, all SUs, and nateglinide were associated with significantly higher rates of confirmed hypoglycemia compared with placebo upon NMA (RR range, 4.00–11.67) (Table 3). All GLP-1 analogs, DPP-4 inhibitors, TZDs, repaglinide and acarbose were not associated with an increased risk of confirmed hypoglycemia compared with placebo. In the active drug comparisons, insulin glargine and all SUs were associated with significantly higher rates of confirmed hypoglycemia compared to any SGLT2 or DPP-4 inhibitor (RR range, 4.32–71.29). There were no data to evaluate glibenclamide, colesevelam and miglitol for this endpoint.

**Table 3:** Results of Network Meta-Analysis and Traditional Meta-Analysis Comparing Antidiabetic Therapies’ Effect on Confirmed Hypoglycemia, Urinary and Genital Tract Infection.

	Confirmed Hypoglycemia			Urinary Tract Infection			Genital Tract Infection		
Comparison vs. Placebo	No. of Trials ^a^	Direct Estimate, RR (95%CI)	NMA Estimate, RR (95%CI)	No. of Trials ^a^	Direct Estimate, RR (95%CI)	NMA Estimate, RR (95%CI)	No. of Trials ^a^	Direct Estimate, RR (95%CI)	NMA Estimate, RR (95%CI)
**Acarbose**	1	0.51 (0.05, 5.47)	0.51 (0.04, 7.23)	0	—	—	0	—	—
**Alogliptin**	1	0.16 (0.03, 0.97)	0.16 (0.02, 1.4)	2	1.05 (0.51, 2.15)	1.06 (0.51,2.17)	0	—	—
**Alogliptin/pioglitazone**	0	—	—	1	0.80 (0.34, 1.89)	0.87 (0.41,1.85)	0	—	—
**Canagliflozin**	2	1.55 (0.43, 5.62)	0.91 (0.33, 2.51)	2	1.38 (0.18, 10.90)	1.25 (0.78,2)	2	5.85 (1.39, 24.65)	8.03 (2.44,26.39)
**Colesevelam**	0	—	—	0	—	—	0	—	—
**Dapagliflozin**	2	1.50 (0.54, 4.18)	0.97 (0.34, 2.76)	1	0.96 (0.48, 1.93)	1.28 (0.77,2.14)	1	2.16 (0.97, 4.79)	2.16 (0.97,4.82)
**Empagliflozin**	1	3.45 (0.43, 27.86)	0.51 (0.17, 1.49)	2	1.20 (0.63, 2.32)	0.86 (0.57,1.3)	2	11.70 (1.59, 86.34)	6.84 (1.92,24.37)
**Emapgliflozin/linagliptin**	0	—	0.38 (0.06, 2.34)	0	—	0.6 (0.31,1.19)	0	—	2.95 (0.66,13.28)
**Exenatide**	1	0.93 (0.35, 2.45)	1.82 (0.7, 4.76)	0	—	—	0	—	—
**Insulin glargine**	0	—	6.78(1.46, 31.4)	0	—	—	0	—	—
**Glibenclamide**	0	—	—	0	—	—	0	—	—
**Gliclazide**	0	—	10.02 (2.07, 48.56)	0	—	—	0	—	—
**Glimepiride**	2	2.19 (0.42,11.50)	4.0 (2.16, 7.41)	0	—	0.89 (0.59,1.33)	0	—	1.28 (0.36,4.53)
**Glipizide**	1	4.5 (1.01, 19.98)	11.67 (4.41, 30.87)	0	—	0.55 (0.29,1.04)	0	—	0.47 (0.17,1.33)
**Linagliptin**	2	0.30 (0.09, 0.97)	0.46 (0.18, 1.2)	1	0.77 (0.32, 1.85)	0.95 (0.6,1.5)	0	—	0.77 (0.07,8.18)
**Liraglutide**	1	0.88 (0.31, 2.51)	0.78 (0.31, 1.96)	0	—	—	0	—	—
**Lixisenatide**	1	3.48 (0.43, 28.03)	0.93 (0.22, 3.83)	0	—	—	0	—	—
**Miglitol**	0	—	—	0	—	—	0	—	—
**Nateglinide**	1	2.41 (0.28, 20.47)	7.19 (1.34, 38.65)	0	—	—	0	—	—
**Pioglitazone**	0	—	1.28(0.25, 6.48)	1	1.25 (0.5, 3.15)	1.4 (0.65,3.02)	0	—	—
**Repaglinide**	1	19.00 (1.16, 310.66)	18.92 (0.9, 398.91)	0	—	—	0	—	—
**Rosiglitazone**	2	1.35 (0.37, 4.90)	1.01 (0.26, 3.97)	0	—	—	0	—	—
**Saxagliptin**	2	0.68 (0.10, 4.61)	0.88 (0.26, 2.96)	2	1.37 (0.76, 2.46)	1.19 (0.75,1.89)	0	—	—
**Sitagliptin**	5	1.19 (0.31, 4.56)	1.3 (0.62, 2.72)	5	1.02 (0.51, 2.01)	0.96 (0.62,1.48)	3	2.27 (0.52, 9.90)	2.33 (0.62,8.8)
**Vildagliptin**	4	1.79 (0.43, 7.45)	0.75 (0.26, 2.14)	2	1.11 (0.06, 19.52)	0.9 (0.15,5.24)	0	—	—

CI = confidence interval; NMA = network meta-analysis; RR = relative risk

^a^ Number of trials with direct comparisons.

### Urinary and Genital Tract Infection

No treatment was associated with an increased risk of UTI when compared to placebo; however, there were no data to evaluate 13 (52%) of the agents for this endpoint (S7 Fig). NMA suggested canagliflozin and empagliflozin were associated with an increased risk of GTI when compared with placebo; with dapagliflozin (RR 2.16, 95% CI 0.97–4.82) trending towards an increased risk versus placebo. However, only 10 identified RCTs evaluating 8 of 25 agents reported GTI data (S8 Fig).

### Direct and Indirect Meta-Analysis Coherence

Results from all pair-wise traditional meta-analyses for each of the six endpoints are reported in S2–S7 Tables. When direct evidence stemming from traditional pair-wise meta-analysis (or a single RCT) was compared to the indirect evidence estimates, we found incoherence in eight of the 151 possible comparisons (2 for the HbA1c endpoint, 2 for body weight, and 4 for confirmed hypoglycemia).

### Sensitivity Analysis

Results from the sensitivity analysis are reported in S9–S14 Figs. The HbA1c endpoint found 91 of 124 comparisons that were still statistically significant at the more stringent 99% CI cutoff (resulting in only 33 comparisons that were changed). For the BW endpoint, only 24 comparisons became nonsignificant (from 219 to 195). For the change in SBP, originally 27 comparisons were significant; however using the stricter set of CIs, only about half (15) remained significant. For the confirmed hypoglycemia endpoint, 74 significant comparisons decreased to 39 after a stricter CI cutoff was applied. No comparisons were altered in the UTI endpoint after the application of a more stringent cutoff and only 4 changed (originally 12) for the GTI endpoint.

## Discussion

When HbA1c goals are not met or maintained with lifestyle modifications and metformin monotherapy, the ADA/EASD guidelines recommend patients initiate an additional antidiabetic agent from 1 of 5 classes (SUs, TZDs, DPP-4 inhibitors, GLP-1 analogs or basal insulin) but do not endorse specific agents in the overall Type 2 DM population or specific patient subtypes [2]. These guidelines were published before SGLT2 inhibitors were commercially available. Our NMA is the first to assess the comparative efficacy and safety of adjunctive therapy with agents in the 10 antidiabetic classes, including the new SGLT2 inhibitors, combination therapies and colesevelam (which is mentioned in current guidelines), when added to optimized but inadequate metformin therapy.

Our HbA1c results showed both statistical differences and clinical superiority between antidiabetic therapies. All therapies significantly reduced HbA1c, but to differing degrees when compared to placebo. Combination therapies (empagliflozin/linagliptin and alogliptin/pioglitazone) and insulin glargine were statistically and clinically superior in reducing HbA1c compared to a majority of other antidiabetic agents. As a class, the SGLT2 inhibitors were similar in efficacy to other non-insulin monotherapies recommended by the ADA as add-ons to metformin, which warrants an update to clinical practice guidelines to include them as a treatment option. Since the FDA uses a 0.3–0.4% noninferiority margin to assess clinical comparative efficacy, colesevelam, nateglinide and miglitol were not clinically superior to placebo (although they did statistically significantly decrease HbA1c) making them less attractive as adjunctive therapies [7].

A therapy’s efficacy, or ability to lower HbA1c, must be weighed against a therapy’s propensity to cause hypoglycemia. In fact, guidelines emphasize the importance of preventing even mild hypoglycemia [2]. Insulin glargine and the SUs caused significantly higher rates of confirmed hypoglycemia when compared to SGLT2 inhibitors, DPP-4 inhibitors and placebo making them less attractive options for patients with Type 2 DM. Of note, we required studies to report the more rigorous definition of “confirmed” hypoglycemia to be included in our meta-analysis. While this endpoint likely carries greater validity than other hypoglycemia definitions, it is also associated with lower event rates making it more difficult to detect differences between therapies.

The majority of patients with Type 2 DM are overweight which may have notable impacts on insulin resistance, glycemic control and cardiovascular risk [2, 73]. SUs, insulin glargine, TZDs, repaglinide and alogliptin/pioglitazone were associated with weight gain compared to placebo. SGLT2 inhibitors, GLP-1 analogs, empagliflozin/linagliptin and miglitol resulted in weight loss, while all other medications were weight neutral compared to placebo. There is no universal threshold for clinically significant weight loss, however reductions greater than 2.3 kg (5 pounds) have been operationally defined [8,72] and associated with improved comorbid outcomes (e.g. improved serum lipids and SBP) [73]. In our analysis, no agent was found to be clinically superior to placebo. However, SGLT2 inhibitors were superior, both statistically and clinically, to SUs, TZDs and insulin glargine for weight loss.

SGLT2 inhibitors, empagliflozin/linagliptin, liraglutide and sitagliptin were associated with a significant decrease in SBP compared with placebo. No antidiabetic therapy met our *a priori* definition of clinical superiority (lower bound of the 95%CI depicting a decrease in SBP of at least 5 mmHg [9]) over other active agents or placebo. The SBP lowering effect of SGLT2 inhibitors observed in our meta-analysis are consistent with a recently published meta-analysis by Baker and colleagues [74] which reported significant reductions in SBP with SGLT2 inhibitors when compared to placebo (WMD -3.8 mmHg, 95%CI -4.4 to -3.2) and active comparators (WMD -4.2 mmHg, 95%CI -4.9 to -3.5).

No agents in any therapy class were found to increase the risk of UTI when compared to placebo. SGLT2 inhibitors were associated with an increased risk of GTI but there was limited reporting of this outcome in RCTs, and future studies are warranted to confirm these findings.

Our NMA has several strengths worth noting. We used stringent inclusion criteria for defining metformin use to assure we were truly assessing therapy in addition to stable metformin therapy. Our NMA is the most comprehensive to date. We evaluated individual agents as compared to classes to assess whether there were within class differences in effect and included almost double the number of trials and therapy comparisons than in previously published NMAs [5, 75]. For comparisons previously assessed in other meta-analyses, our results were generally consistent [5, 75–77]. However, even here we included a substantial amount of additional data on DPP-4 inhibitors and GLP-1 analogs, which had limited data to assess their efficacy/safety in previous meta-analyses. We included SGLT2 inhibitors in our NMA. NMAs focusing on SGLT2 inhibitors have been published, but did not include all potential antidiabetic therapies in their network, thus suppressing potential indirect data [78]. Methods papers suggest not including all possible and relevant linked therapies can negatively impact conclusions of a NMA. We assessed outcomes like UTI and GTI not included in previous meta-analyses which augments the safety data available for interclass comparisons.

Our NMA has several limitations. We saw little evidence of incoherence in our network (as evidenced by finding only 8 out of 151 possible direct versus indirect evidence comparisons statistically significant); however, incoherence cannot be completely ruled out. The eight statistically significant findings resulting from 151 statistical tests are consistent with 7–8 findings that would be expected due to chance alone. We only included English-language RCTs, but due to the vast number of trials included in our meta-analysis, a limited number of trials missed for this reason would likely have minimal impact on our results. We found sparse reporting of certain endpoints (UTI and GTI) which makes it difficult to draw concrete conclusions (potential for type 2 error). At the same time, our NMA cross- compared 25 therapies plus placebo on 6 different endpoints; thus multiple hypothesis testing can lead to erred conclusions of statistical differences between therapies when they do not truly exist (type 1 error). However, we conducted a sensitivity analysis using 99% CIs as a more stringent cutoff for statistical significance and found the majority of comparisons to still be statistically significant. Other agent-specific endpoints (e.g. cardiovascular events, pancreatitis, renal dysfunction, heart failure) could not be assessed due to the limited data and short follow-up periods of the included trials. Lastly, publication bias is always a concern in a meta-analysis, but we minimized this concern with our systematic search strategy, backwards citation tracking and multi-pronged grey literature searches.

## Conclusions

In persons with Type 2 DM inadequately-controlled on stable metformin monotherapy, all agents analyzed in our NMA proved effective (lowered HbA1c vs. placebo) but with differing reductions in HbA1c. Evaluated agents differed in their effects on BW, SBP, hypoglycemia and GTI; however no medication increased the risk of UTI when compared to placebo. The newest class of antidiabetic agents, the SGLT2 inhibitors, was found to provide similar HbA1c efficacy to other non-insulin monotherapies (albeit not oral combination therapies) with the added benefits of weight loss, reduced SBP and a low risk of hypoglycemia; but at a cost of an increased risk of GTI. Combination therapies resulted in some of the largest reductions in HbA1c and may be appropriate for patients requiring profound (>1%) HbA1c reductions after failing optimized metformin.

## Supporting Information

S1 Checklist Prisma Checklist. (DOC)

S1 Appendix Medline Search Strategy. (PDF)

S2 Appendix Grading Parameters for the Cochrane Risk of Bias Tool. GTI = genital tract infection; HbA1c = glycated hemoglobin; IVRS = interactive voice response system; SGLT2 = sodium glucose co-transporter-2; SU = sulfonylurea; TZD = thiazolidinedione; UTI = urinary tract infection.
(PDF)

S1 Fig Guide for Determining Statistical Significance and Clinical Superiority. In this figure (x) is defined as the minimally important clinical difference between two treatments (or a treatment and placebo). Using the example of HbA1c reduction, line A displays an HbA1c difference between therapies that is not statistically significant because the 95% confidence interval (CI) cross the line of no effect; lines B and C both show drug A to be statistically significantly better at lowering HbA1c than drug B, but that clinical superiority cannot be claimed in either case because the lower bound of the 95% CI crosses the (dashed) line marking the minimally important clinical difference, (MICD) x (or in the case of HbA1c reduction, 0.3%); line D depicts a situation where drug A is both statistically significant and clinically superior in reducing HbA1c compared to drug B as evidenced by the lower bound of the 95%CI not crossing the line marking the MICD. In our network meta-analysis, the comparison of empagliflozin/linagliptin vs. dapagliflozin (change in HbA1c = -0.65, 95%CI -0.91, -0.39) would met these criteria for statistical significance and clinical superiority. MICD = minimally important clinical difference; RR = relative risk.
(PDF)

S2 Fig Risk of Bias Assessment of Randomized Controlled Trials. + = low risk of bias;? = unclear risk of bias;– = high risk of bias.
(PDF)

S3 Fig Network Meta-Analysis Results of the Effect of Antidiabetic Therapies on Change in HbA1c From Baseline. Therapies are reported in alphabetical order. HbA1c results are reported in WMD, % (95% CI). Results for changes in HbA1c on the top portion of the matrix represent changes in the row-defining treatment vs. those in the column-defining treatment (referent). For changes in HbA1c, negative values favor the first agent in alphabetical order. Statistically significant results are bolded. Clinically superior results are underlined. Sodium glucose co-transporter-2 (SGLT-2) inhibitors are highlighted. The results on the bottom portion of the matrix represent the reciprocal of the top portion. ACA = acarbose; ALO = alogliptin; ALO/PIO = alogliptin/pioglitazone; CANA = canagliflozin; COL = colesevelam; DAPA = dapagliflozin; EMPA = empagliflozin; EMPA/LINA = empagliflozin/linagliptin; EXEN = exenatide; GLAR = glargine; GLIB = glibenclamide; GLIC = gliclazide; GLIM = glimepiride; GLIP = glipizide; HbA1c = hemoglobin A1c; LINA = linagliptin; LIRA = liraglutide; LIX = lixisenatide; MIG = miglitol; NAT = nateglinide; PIO = pioglitazone; PLC = placebo; REP = repaglinide; ROSI = rosiglitazone; SAX = saxagliptin; SITA = sitagliptin; VILDA = vildagliptin.
(PDF)

S4 Fig Network Meta-Analysis Results of the Effect of Antidiabetic Therapies on Change in Body Weight From Baseline. Therapies are reported in alphabetical order. Results are reported in WMD, kg (95% CI). Results for changes in weight on the top portion of the matrix represent changes in the row-defining treatment vs. those in the column-defining treatment (referent). For changes in weight, negative values favor the first agent in alphabetical order. Statistically significant results are bolded. Clinically superior results are underlined. Sodium glucose co-transporter-2 (SGLT-2) inhibitors are highlighted. The results on the bottom portion of the matrix represent the reciprocal of the top portion. ACA = acarbose; ALO = alogliptin; ALO/PIO = alogliptin/pioglitazone; CANA = canagliflozin; COL = colesevelam; DAPA = dapagliflozin; EMPA = empagliflozin; EMPA/LINA = empagliflozin/linagliptin; EXEN = exenatide; GLAR = glargine; GLIB = glibenclamide; GLIC = gliclazide; GLIM = glimepiride; GLIP = glipizide; LINA = linagliptin; LIRA = liraglutide; LIX = lixisenatide; MIG = miglitol; NAT = nateglinide; PIO = pioglitazone; PLC = placebo; REP = repaglinide; ROSI = rosiglitazone; SAX = saxagliptin; SITA = sitagliptin; VILDA = vildagliptin.
(PDF)

S5 Fig Network Meta-Analysis Results of the Effect of Antidiabetic Therapies on Change in Systolic Blood Pressure From Baseline. Therapies are reported in alphabetical order. Results are reported in WMD, mmHg (95% CI). Results for changes in systolic blood pressure (SBP) on the top portion of the matrix represent changes in the row-defining treatment vs. those in the column-defining treatment (referent). For changes in SBP, negative values favor the first agent in alphabetical order. Statistically significant results are bolded. Clinically superior results are underlined. Sodium glucose co-transporter-2 (SGLT-2) inhibitors are highlighted. The results on the bottom portion of the matrix represent the reciprocal of the top portion. CANA = canagliflozin; DAPA = dapagliflozin; EMPA = empagliflozin; EMPA/LINA = empagliflozin/linagliptin; EXEN = exenatide; GLIM = glimepiride; GLIP = glipizide; LINA = linagliptin; LIRA = liraglutide; PIO = pioglitazone; PLC = placebo; SAX = saxagliptin; SITA = sitagliptin; VILDA = vildagliptin.
(PDF)

S6 Fig Network Meta-Analysis Results of the Effect of Antidiabetic Therapies on Risk of Confirmed Hypoglycemia. Therapies are reported in alphabetical order. Results for risk of confirmed hypoglycemia on the top portion of the matrix represent relative risks (RRs) of hypoglycemia in the row-defining treatment vs. those the column-defining treatment (referent). For confirmed hypoglycemia, RRs lower than 1 favor the first agent in alphabetical order. Statistically significant results are bolded. Sodium glucose co-transporter-2 (SGLT-2) inhibitors are highlighted. To obtain RRs for comparisons in the opposite direction, reciprocals should be taken or the lower portion of the matrix can be used. ACA = acarbose; ALO = alogliptin; CANA = canagliflozin; DAPA = dapagliflozin; EMPA = empagliflozin; EMPA/LINA = empagliflozin/linagliptin; EXEN = exenatide; GLAR = glargine; GLIC = gliclazide; GLIM = glimepiride; GLIP = glipizide; LINA = linagliptin; LIRA = liraglutide; LIX = lixisenatide; NAT = nateglinide; PIO = pioglitazone; PLC = placebo; REP = repaglinide; ROSI = rosiglitazone; SAX = saxagliptin; SITA = sitagliptin; VILDA = vildagliptin.
(PDF)

S7 Fig Network Meta-Analysis Results of the Effect of Antidiabetic Therapies on Risk of Urinary Tract Infections. Therapies are reported in alphabetical order. Results for risk of urinary tract infection (UTI) on the top portion of the matrix represent relative risks (RRs) of UTI in the row-defining treatment vs. those the column-defining treatment (referent). For UTI, RRs lower than 1 favor the first agent in alphabetical order. Statistically significant results are bolded. Sodium glucose co-transporter-2 (SGLT-2) inhibitors are highlighted. To obtain RRs for comparisons in the opposite direction, reciprocals should be taken or the lower portion of the matrix can be used. ALO/PIO = alogliptin/pioglitazone; ALO = alogliptin; CANA = canagliflozin; DAPA = dapagliflozin; EMPA = empagliflozin; EMPA/LINA = empagliflozin/linagliptin; GLIM = glimepiride; GLIP = glipizide; LINA = linagliptin; PIO = pioglitazone; PLC = placebo; SAX = saxagliptin; SITA = sitagliptin; VILDA = vildagliptin.
(PDF)

S8 Fig Network Meta-Analysis Results of the Effect of Antidiabetic Therapies on Risk of Genital Tract Infections. Therapies are reported in alphabetical order. Results for risk of genital tract infection (GTI) on the top portion of the matrix represent relative risks (RRs) of GTI in the row-defining treatment vs. those the column-defining treatment (referent). For GTI, RRs lower than 1 favor the first agent in alphabetical order. Statistically significant results are bolded. Sodium glucose co-transporter-2 (SGLT-2) inhibitors are highlighted. To obtain RRs for comparisons in the opposite direction, reciprocals should be taken or the lower portion of the matrix can be used. CANA = canagliflozin; DAPA = dapagliflozin; EMPA = empagliflozin; EMPA/LINA = empagliflozin/linagliptin; GLIM = glimepiride; GLIP = glipizide; LINA = linagliptin; PLC = placebo; SITA = sitagliptin.
(PDF)

S9 Fig Sensitivity Analysis Results of the Effect of Antidiabetic Therapies on Change in HbA1c From Baseline. Therapies are reported in alphabetical order. HbA1c results are reported in WMD, % (99% CI). Results for changes in HbA1c on the top portion of the matrix represent changes in the row-defining treatment vs. those in the column-defining treatment (referent). For changes in HbA1c, negative values favor the first agent in alphabetical order. Statistically significant results of the sensitivity analysis are colored grey. Sodium glucose co-transporter-2 (SGLT-2) inhibitors are highlighted. The results on the bottom portion of the matrix represent the reciprocal of the top portion. ACA = acarbose; ALO = alogliptin; ALO/PIO = alogliptin/pioglitazone; CANA = canagliflozin; COL = colesevelam; DAPA = dapagliflozin; EMPA = empagliflozin; EMPA/LINA = empagliflozin/linagliptin; EXEN = exenatide; GLAR = glargine; GLIB = glibenclamide; GLIC = gliclazide; GLIM = glimepiride; GLIP = glipizide; HbA1c = hemoglobin A1c; LINA = linagliptin; LIRA = liraglutide; LIX = lixisenatide; MIG = miglitol; NAT = nateglinide; PIO = pioglitazone; PLC = placebo; REP = repaglinide; ROSI = rosiglitazone; SAX = saxagliptin; SITA = sitagliptin; VILDA = vildagliptin.
(PDF)

S10 Fig Sensitivity Analysis Results of the Effect of Antidiabetic Therapies on Change in Body Weight From Baseline. Therapies are reported in alphabetical order. Results are reported in WMD, kg (95% CI). Results for changes in weight on the top portion of the matrix represent changes in the row-defining treatment vs. those in the column-defining treatment (referent). For changes in weight, negative values favor the first agent in alphabetical order. Statistically significant results of the sensitivity analysis are colored grey. Sodium glucose co-transporter-2 (SGLT-2) inhibitors are highlighted. The results on the bottom portion of the matrix represent the reciprocal of the top portion. ACA = acarbose; ALO = alogliptin; ALO/PIO = alogliptin/pioglitazone; CANA = canagliflozin; COL = colesevelam; DAPA = dapagliflozin; EMPA = empagliflozin; EMPA/LINA = empagliflozin/linagliptin; EXEN = exenatide; GLAR = glargine; GLIB = glibenclamide; GLIC = gliclazide; GLIM = glimepiride; GLIP = glipizide; LINA = linagliptin; LIRA = liraglutide; LIX = lixisenatide; MIG = miglitol; NAT = nateglinide; PIO = pioglitazone; PLC = placebo; REP = repaglinide; ROSI = rosiglitazone; SAX = saxagliptin; SITA = sitagliptin; VILDA = vildagliptin.
(PDF)

S11 Fig Sensitivity Analysis Results of the Effect of Antidiabetic Therapies on Change in Systolic Blood Pressure From Baseline. Therapies are reported in alphabetical order. Results are reported in WMD, mmHg (95% CI). Results for changes in systolic blood pressure (SBP) on the top portion of the matrix represent changes in the row-defining treatment vs. those in the column-defining treatment (referent). For changes in SBP, negative values favor the first agent in alphabetical order. Statistically significant results of the sensitivity analysis are colored grey. Sodium glucose co-transporter-2 (SGLT-2) inhibitors are highlighted. The results on the bottom portion of the matrix represent the reciprocal of the top portion. CANA = canagliflozin; DAPA = dapagliflozin; EMPA = empagliflozin; EMPA/LINA = empagliflozin/linagliptin; EXEN = exenatide; GLIM = glimepiride; GLIP = glipizide; LINA = linagliptin; LIRA = liraglutide; PIO = pioglitazone; PLC = placebo; SAX = saxagliptin; SITA = sitagliptin; VILDA = vildagliptin.
(PDF)

S12 Fig Sensitivity Analysis Results of the Effect of Antidiabetic Therapies on Risk of Confirmed Hypoglycemia. Therapies are reported in alphabetical order. Results for risk of confirmed hypoglycemia on the top portion of the matrix represent relative risks (RRs) of hypoglycemia in the row-defining treatment vs. those the column-defining treatment (referent). For confirmed hypoglycemia, RRs lower than 1 favor the first agent in alphabetical order. Statistically significant results of the sensitivity analysis are colored grey. Sodium glucose co-transporter-2 (SGLT-2) inhibitors are highlighted. To obtain RRs for comparisons in the opposite direction, reciprocals should be taken or the lower portion of the matrix can be used. ACA = acarbose; ALO = alogliptin; CANA = canagliflozin; DAPA = dapagliflozin; EMPA = empagliflozin; EMPA/LINA = empagliflozin/linagliptin; EXEN = exenatide; GLAR = glargine; GLIC = gliclazide; GLIM = glimepiride; GLIP = glipizide; LINA = linagliptin; LIRA = liraglutide; LIX = lixisenatide; NAT = nateglinide; PIO = pioglitazone; PLC = placebo; REP = repaglinide; ROSI = rosiglitazone; SAX = saxagliptin; SITA = sitagliptin; VILDA = vildagliptin.
(PDF)

S13 Fig Sensitivity Analysis Results of the Effect of Antidiabetic Therapies on Risk of Urinary Tract Infections. Therapies are reported in alphabetical order. Results for risk of urinary tract infection (UTI) on the top portion of the matrix represent relative risks (RRs) of UTI in the row-defining treatment vs. those the column-defining treatment (referent). For UTI, RRs lower than 1 favor the first agent in alphabetical order. Statistically significant results of the sensitivity analysis are colored grey. Sodium glucose co-transporter-2 (SGLT-2) inhibitors are highlighted. To obtain RRs for comparisons in the opposite direction, reciprocals should be taken or the lower portion of the matrix can be used. ALO/PIO = alogliptin/pioglitazone; ALO = alogliptin; CANA = canagliflozin; DAPA = dapagliflozin; EMPA = empagliflozin; EMPA/LINA = empagliflozin/linagliptin; GLIM = glimepiride; GLIP = glipizide; LINA = linagliptin; PIO = pioglitazone; PLC = placebo; SAX = saxagliptin; SITA = sitagliptin; VILDA = vildagliptin.
(PDF)

S14 Fig Sensitivity Analysis Results of the Effect of Antidiabetic Therapies on Risk of Genital Tract Infections. Therapies are reported in alphabetical order. Results for risk of genital tract infection (GTI) on the top portion of the matrix represent relative risks (RRs) of GTI in the row-defining treatment vs. those the column-defining treatment (referent). For GTI, RRs lower than 1 favor the first agent in alphabetical order. Statistically significant results of the sensitivity analysis are colored grey. Sodium glucose co-transporter-2 (SGLT-2) inhibitors are highlighted. To obtain RRs for comparisons in the opposite direction, reciprocals should be taken or the lower portion of the matrix can be used. CANA = canagliflozin; DAPA = dapagliflozin; EMPA = empagliflozin; EMPA/LINA = empagliflozin/linagliptin; GLIM = glimepiride; GLIP = glipizide; LINA = linagliptin; PLC = placebo; SITA = sitagliptin.
(PDF)

S1 Table Baseline Characteristics of Randomized Controlled Trials Evaluating Antidiabetic Therapies in Addition to Metformin in Adults with Type 2 Diabetes. BID = twice daily; BMI = body mass index; DM = diabetes mellitus; FPG = fasting plasma glucose; HbA1c = hemoglobin A1c; n = number of patients; NR = not reported; OAD = oral antidiabetic drug; SBP = systolic blood pressure; SU = sulfonylurea; SE = standard error; TID = three times a day; qAM = daily AM dosing; qPM = daily PM dosing; QW = weekly. ^a^Number of study participants evaluated for change in A1c from baseline (sample size may vary for other endpoints). ^b^Doses were given daily, unless otherwise specified. ^c^Data given in median (range).
(PDF)

S2 Table Results of Traditional Meta-Analysis Comparing Antidiabetic Therapies Effect on HbA1c. ACA = acarbose; ALO = alogliptin; ALO/PIO = alogliptin/pioglitazone; CANA = canagliflozin; COL = colesevelam; DAPA = dapagliflozin; EMPA = empagliflozin; EMPA/LINA = empagliflozin/linagliptin; EXEN = exenatide; GLAR = insulin glargine; GLIB = glibenclamide; GLIC = gliclazide; GLIM = glimepiride; GLIP = glipizide; LINA = linagliptin; LIRA = liraglutide; LIX = lixisenatide; MIG = miglitol; NAT = nateglinide; PIO = pioglitizone; PLC = placebo; REP = repaglinide; ROSI = rosiglitizone; SAX = saxagliptin; SITA = sitagliptin; VILDA = vildagliptin. ^a^I^2^>50%.
(PDF)

S3 Table Results of Traditional Meta-Analysis Comparing Antidiabetic Therapies Effect on Body Weight. ACA = acarbose; ALO = alogliptin; ALO/PIO = alogliptin/pioglitazone; CANA = canagliflozin; COL = colesevelam; DAPA = dapagliflozin; EMPA = empagliflozin; EMPA/LINA = empagliflozin/linagliptin; EXEN = exenatide; GLAR = insulin glargine; GLIB = glibenclamide; GLIC = gliclazide; GLIM = glimepiride; GLIP = glipizide; LINA = linagliptin; LIRA = liraglutide; LIX = lixisenatide; MIG = miglitol; NAT = nateglinide; PIO = pioglitizone; PLC = placebo; REP = repaglinide; ROSI = rosiglitizone; SAX = saxagliptin; SITA = sitagliptin; VILDA = vildagliptin. ^a^I^2^>50%.
(PDF)

S4 Table Results of Traditional Meta-Analysis Comparing Antidiabetic Therapies Effect on Systolic Blood Pressure. CANA = canagliflozin; DAPA = dapagliflozin; EMPA = empagliflozin; EMPA/LINA = empagliflozin/linagliptin; EXEN = exenatide; GLAR = insulin glargine; GLIM = glimepiride; GLIP = glipizide; LINA = linagliptin; LIRA = liraglutide; PIO = pioglitizone; PLC = placebo; SAX = saxagliptin; SBP = systolic blood pressure; SITA = sitagliptin; VILDA = vildagliptin. ^a^Egger’s p-value <0.05
(PDF)

S5 Table Results of Traditional Meta-Analysis Comparing Antidiabetic Therapies Effect on Experiencing Confirmed Hypoglycemia. ACA = acarbose; ALO = alogliptin; CANA = canagliflozin; DAPA = dapagliflozin; EMPA = empagliflozin; EMPA/LINA = empagliflozin/linagliptin; EXEN = exenatide; GLAR = insulin glargine; GLIC = gliclazide; GLIM = glimepiride; GLIP = glipizide; LINA = linagliptin; LIRA = liraglutide; LIX = lixisenatide; NAT = nateglinide; PIO = pioglitizone; PLC = placebo; REP = repaglinide; ROSI = rosiglitizone; SAX = saxagliptin; SITA = sitagliptin; VILDA = vildagliptin.
(PDF)

S6 Table Results of Traditional Meta-Analysis Comparing Antidiabetic Therapies Effect on Experiencing Urinary Tract Infections. ALO = alogliptin; ALO/PIO = alogliptin/pioglitazone; CANA = canagliflozin; DAPA = dapagliflozin; EMPA = empagliflozin; EMPA/LINA = empagliflozin/linagliptin; GLIM = glimepiride; GLIP = glipizide; LINA = linagliptin; PIO = pioglitizone; PLC = placebo; SAX = saxagliptin; SITA = sitagliptin; VILDA = vildagliptin.
(PDF)

S7 Table Results of Traditional Meta-Analysis Comparing Antidiabetic Therapies Effect on Experiencing Genital Tract Infections. CANA = canagliflozin; DAPA = dapagliflozin; EMPA = empagliflozin; EMPA/LINA = empagliflozin/linagliptin; GLIM = glimepiride; GLIP = glipizide; LINA = linagliptin; PLC = placebo; SITA = sitagliptin.
(PDF)
