# Supplementary material for: Comparative Efficacy and Safety of Antidiabetic Drug Regimens Added to Metformin Monotherapy in Patients with Type 2 Diabetes: A Network Meta-Analysis
Source: PLoS One. 2015 Apr 28;10(4):e0125879. doi: 10.1371/journal.pone.0125879 (PMC4412636; doi:10.1371/journal.pone.0125879)
Supplement: S1 Appendix — (PDF) [file pone.0125879.s002.pdf]

Appendix S1. Medline Search Strategy

1. Metformin.mp or Metformin/
2. Type 2 diabetes mellitus.mp or Diabetes Mellitus, Type 2/
3. T2D.mp
4. Noninsulin dependent diabetes.mp
5. NIDDM.mp
6. Type 2 DM.mp
7. Glycosylated hemoglobin.mp or Hemoglobin A, Glycosylated/
8. Glycated hemoglobin.mp
9. Hemoglobin a1c.mp
10. Hba1c.mp
11. A1c.mp
12. OR/2-11
13. 1 AND 12*

*Limited to randomized controlled trials
