# Supplementary material for: Comparative Efficacy and Safety of Antidiabetic Drug Regimens Added to Metformin Monotherapy in Patients with Type 2 Diabetes: A Network Meta-Analysis
Source: PLoS One. 2015 Apr 28;10(4):e0125879. doi: 10.1371/journal.pone.0125879 (PMC4412636; doi:10.1371/journal.pone.0125879)
Supplement: S2 Appendix — GTI = genital tract infection; HbA1c = glycated hemoglobin; IVRS = interactive voice response system; SGLT2 = sodium glucose co-transporter-2; SU = sulfonylurea; TZD = thiazolidinedione; UTI = urinary tract infection. (PDF) [file pone.0125879.s003.pdf]

Appendix S2. Grading Parameters for the Cochrane Risk of Bias Tool

Risk of Bias Domain	Assessment Criteria	
Random Sequence Generation	Low Risk	Participants had an equal opportunity to be placed in each arm of the study. Investigators described a random component of sequence generation (e.g. computer random number generation, shuffling cards or envelopes).
	High Risk	Participants were pre-determined to be placed in a particular arm of the study. Investigators described a non-random component in sequence generation (e.g. odd or even date of birth, date of admission, judgment of the clinician or participant).
	Unclear Risk	There was no mention of the sequence generation process. This included studies that only stated randomization was performed without any further information about the sequence generation process.
Allocation Concealment	Low Risk	Participants and investigators enrolling participants could not foresee assignment due to the use of proper allocation concealment methods (e.g. central allocation including telephone, web-based, and pharmacy-controlled randomization, sequentially numbered, opaque, sealed envelopes or IVRS).
	High Risk	Participants or investigators enrolling participants could foresee assignments into the arms of the study due to improper allocation concealment methods (e.g. use of an open random allocation schedule, alternation or rotation, or assignment envelopes without appropriate safeguards).
	Unclear Risk	There was no mention or insufficient information to permit judgment of 'low' or 'high' risk.
Blinding of Participants and Personnel	Low Risk	The study indicated that the participants and personnel were blinded throughout the study. The study had to mention 'double-blinding' and also indicate who was blinded and the method of blinding (e.g., matching placebo, 'double-dummy' design).
	High Risk	No or incomplete complete blinding of both participants and personnel.
	Unclear Risk	There was insufficient information to determine whether both participants and personnel were sufficiently blinded.
Blinding of Outcome Assessment	Low Risk	The study specifically indicated the outcome assessor was blinded.
	High Risk	The study indicated the outcome assessor knew which arm of the study the participant was allocated.
	Unclear Risk	There was insufficient evidence to determine whether the outcome assessor was blinded.
Incomplete Outcome Data	Low Risk	The study was considered low risk if there was no missing outcome data or if the following criteria were met:<br>• The study filled the gaps in data (missing data) with imputation (e.g., last observation carried forward).<br>• The study utilized an intention to treat (included all randomized patients) or modified intention to treat analysis (included all randomized patients who received one dose and/or had one follow up visit) methodology;<br>• The study did not have more than 20% of participants dropout of the trial after randomization;<br>• The study did not have more than a 10% differential in dropout rate between study arms.
	High Risk	The above-mentioned criteria were not met.
	Unclear Risk	Insufficient reporting of attrition and/or exclusions to allow for judgment of 'low' or 'high' risk assessment.

Selective Reporting	Low Risk	The study reported all endpoints discussed in the methods section and the study included all outcomes expected of a type 2 diabetes study and specific antidiabetic medication classes:<br>• All studies reported HbA1c changes and confirmed hypoglycemia;<br>• SGLT2 inhibitor studies reported the incidences of UTIs and GTIs;<br>• TZD, meglitinide and basal insulin studies reported weight change.
	High Risk	The study did NOT report all endpoints discussed in the methods section and/or did NOT include all outcomes to be expected of the disease state and medication classes discussed above.
	Unclear Risk	There is insufficient information to assess 'low' or 'high' risk
Other Bias	Low Risk	Participants were stable on maximal or near-maximal metformin (≥1500mg/day) and at least half-maximal dose of SU for at least 12 weeks prior to the start of the study. Study was published in a peer-reviewed journal.
	High Risk	Participants were either not on stable, maximal or near-maximal metformin and at least half-maximal dose of SU, received it for less than 12 weeks prior to randomization, and/or the study was not published in a peer-reviewed journal.
	Unclear Risk	There was insufficient information to assess 'low' or 'high' risk.
