# Supplementary figures and images for: Comparative Efficacy and Safety of Antidiabetic Drug Regimens Added to Metformin Monotherapy in Patients with Type 2 Diabetes: A Network Meta-Analysis
Source: PLoS One. 2015 Apr 28;10(4):e0125879. doi: 10.1371/journal.pone.0125879 (PMC4412636; doi:10.1371/journal.pone.0125879)

Figure S1. Guide for Determining Statistical Significance and Clinical Superiority

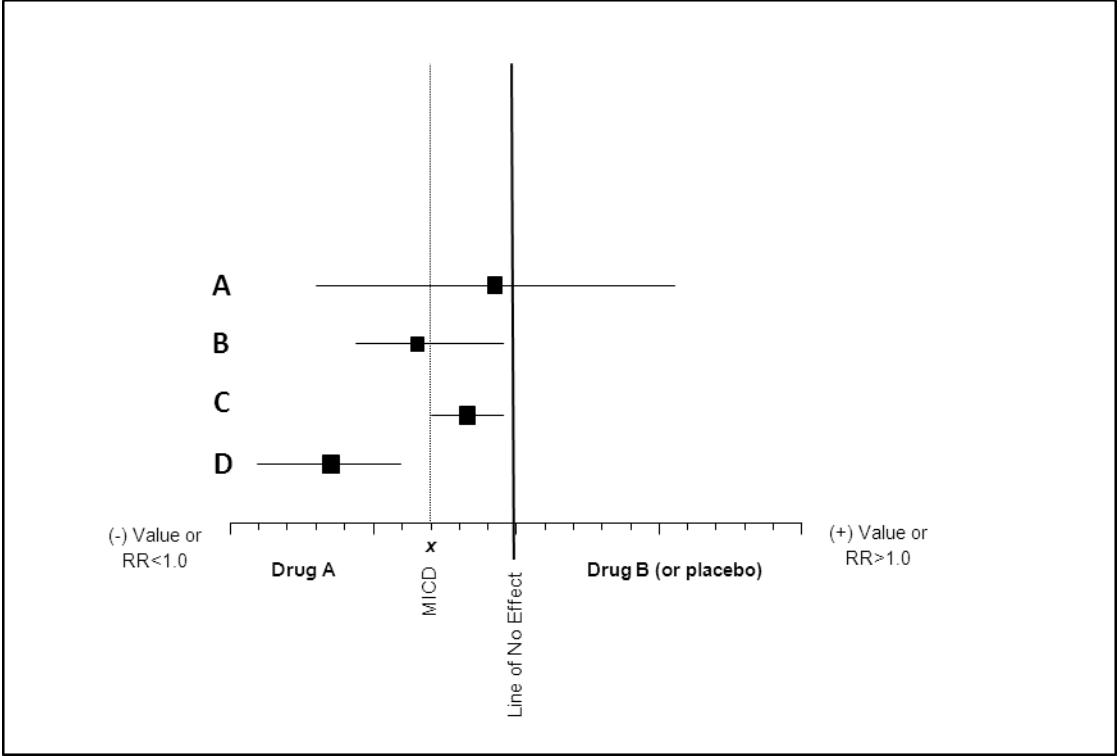

Supplement: S1 Fig — In this figure (x) is defined as the minimally important clinical difference between two treatments (or a treatment and placebo). Using the example of HbA1c reduction, line A displays an HbA1c difference between therapies that is not statistically significant because the 95% confidence interval (CI) cross the line of no effect; lines B and C both show drug A to be statistically significantly better at lowering HbA1c than drug B, but that clinical superiority cannot be claimed in either case because the lower bound of the 95% CI crosses the (dashed) line marking the minimally important clinical difference, (MICD) x (or in the case of HbA1c reduction, 0.3%); line D depicts a situation where drug A is both statistically significant and clinically superior in reducing HbA1c compared to drug B as evidenced by the lower bound of the 95%CI not crossing the line marking the MICD. In our network meta-analysis, the comparison of empagliflozin/linagliptin vs. dapagliflozin (change in HbA1c = -0.65, 95%CI -0.91, -0.39) would met these criteria for statistical significance and clinical superiority. MICD = minimally important clinical difference; RR = relative risk. (PDF) [file pone.0125879.s004.pdf]
