# Supplementary material for: Comparative Efficacy and Safety of Antidiabetic Drug Regimens Added to Metformin Monotherapy in Patients with Type 2 Diabetes: A Network Meta-Analysis
Source: PLoS One. 2015 Apr 28;10(4):e0125879. doi: 10.1371/journal.pone.0125879 (PMC4412636; doi:10.1371/journal.pone.0125879)
Supplement: S2 Fig — + = low risk of bias;? = unclear risk of bias;– = high risk of bias. (PDF) [file pone.0125879.s005.pdf]

Figure S2. Risk of Bias Assessment of Randomized Controlled Trials

	Random sequence generation	Allocation concealment	Blinding of participants and personnel	Blinding of outcomes assessment	Incomplete outcome data	Selective reporting	Other bias
Defronzo 2014	?	?	?	?	+	+	-
Bolli 2014	?	?	+	?	?	+	+
Derosa 2014	+	+	+	?	-	-	?
Haring 2014	+	+	?	?	+	+	+
Nauck 2014	?	?	+	?	+	-	-
Ridderstrale 2014	+	+	+	?	-	+	+
White 2014	+	+	+	?	+	+	-
Charbonnel 2013	+	+	-	?	?	+	+
Chawla 2013	+	?	?	?	-	-	-
Cefalu 2013	+	+	+	+	+	+	-
Derosa 2013	+	+	+	?	?	+	+
Lavalle-Gonzalez 2013	+	+	+	+	+	+	-
Rosenstock 2013	+	+	+	?	+	+	-
Rosenstock 2013b	?	+	-	?	+	+	+
Aschner 2012	+	+	-	?	?	+	-
Bergenstal 2012	+	+	?	+	-	-	+
DeFronzo 2012	?	?	+	?	-	-	-
Derosa 2012	?	+	+	?	+	+	+
Derosa 2012b	?	+	+	?	+	-	+
Gallwitz 2012	+	+	-	?	-	+	?
Gallwitz 2012b	+	+	+	+	-	+	-
Ljunggren 2012	+	+	+	+	+	+	+
Pan 2012	?	?	?	?	+	+	-
Rizzo 2012	?	?	-	+	+	-	-
Rosenstock 2012	?	?	?	?	+	+	+
Ross 2012	+	+	+	+	+	+	+
Arechavaleta 2011	+	+	+	?	+	+	+
Nauck 2011	+	+	+	?	-	+	-
Pfützner 2011	?	?	?	?	+	+	+
Taskinen 2011	?	?	?	?	+	+	+
Wang 2011	+	-	-	?	?	-	-
Yang 2011	+	?	+	?	+	+	-
Bailey 2010	+	+	+	+	+	+	-
Filozof 2010	?	?	+	?	+	-	-
Goke 2010	+	+	+	?	-	+	-
Pratley 2010	+	+	-	+	-	+	+
Rigby 2010	?	?	-	?	-	-	-
Scheen 2010	?	?	+	?	+	+	-
DeFronzo 2009	+	+	+	?	-	+	-
Ferrannini 2009	?	?	+	?	-	+	-
Goodman 2009	?	?	?	?	+	+	+
Nauck 2009	+	+	?	?	-	+	+
Nauck 2009b	+	+	+	?	-	+	+
Hamann 2008	+	+	+	?	-	+	+
Khanolkar 2008	?	?	-	?	+	-	-
Raz 2008	+	?	?	?	+	+	-
Scott 2008	?	?	?	?	+	+	-

Bosi 2007	?	?	?	?	+	+	-
Nauck 2007	?	?	?	?	+	+	-
Ristic 2006	+	+	+	?	+	+	-
DeFronzo 2005	?	?	+	?	+	+	+
Feinglos 2005	?	?	?	?	+	+	+
Matthews 2005	?	?	+	?	+	+	+
Ahren 2004	?	?	?	?	+	+	+
Gomez-Perez 2002	?	?	?	?	-	+	-
Marre 2002	+	+	+	?	+	+	-
Charpentier 2001	+	+	+	?	+	+	-
Van 2001	+	?	+	?	-	+	+
Halimi 2000	?	?	?	?	+	-	-
Fonseca 2000	+	?	+	+	+	+	-
Moses 1999	?	?	?	?	+	+	+
Rosenstock 1998	?	?	?	?	-	+	+
