# Supplementary material for: Comparative Efficacy and Safety of Antidiabetic Drug Regimens Added to Metformin Monotherapy in Patients with Type 2 Diabetes: A Network Meta-Analysis
Source: PLoS One. 2015 Apr 28;10(4):e0125879. doi: 10.1371/journal.pone.0125879 (PMC4412636; doi:10.1371/journal.pone.0125879)
Supplement: S3 Fig — Therapies are reported in alphabetical order. HbA1c results are reported in WMD, % (95% CI). Results for changes in HbA1c on the top portion of the matrix represent changes in the row-defining treatment vs. those in the column-defining treatment (referent). For changes in HbA1c, negative values favor the first agent in alphabetical order. Statistically significant results are bolded. Clinically superior results are underlined. Sodium glucose co-transporter-2 (SGLT-2) inhibitors are highlighted. The results on the bottom portion of the matrix represent the reciprocal of the top portion. ACA = acarbose; ALO = alogliptin; ALO/PIO = alogliptin/pioglitazone; CANA = canagliflozin; COL = colesevelam; DAPA = dapagliflozin; EMPA = empagliflozin; EMPA/LINA = empagliflozin/linagliptin; EXEN = exenatide; GLAR = glargine; GLIB = glibenclamide; GLIC = gliclazide; GLIM = glimepiride; GLIP = glipizide; HbA1c = hemoglobin A1c; LINA = linagliptin; LIRA = liraglutide; LIX = lixisenatide; MIG = miglitol; NAT = nateglinide; PIO = pioglitazone; PLC = placebo; REP = repaglinide; ROSI = rosiglitazone; SAX = saxagliptin; SITA = sitagliptin; VILDA = vildagliptin. (PDF) [file pone.0125879.s006.pdf]

## Figure S3. Network Meta-Analysis Results of the Effect of Antidiabetic Therapies on Change in HbA1c From Baseline

| | | | | | | | | | | | | | | | | | | | | | | | | | |
|---|---|---|---|---|---|---|---|---|---|---|---|---|---|---|---|---|---|---|---|---|---|---|---|---|---|
| ACA | -0.21 (-0.57,0.15) | **0.45 (0.08, 0.82)** | -0.07 (-0.4,0.26) | -0.29 (-0.76,0.18) | -0.31 (-0.65, 0.03) | -0.1 (-0.43, 0.23) | 0.34 (-0.03, 0.72) | 0.01 (-0.32, 0.34) | **0.45 (0.04, 0.85)** | 0.5 (-0.15, 1.15) | -0.08 (-0.42, 0.26) | -0.06 (-0.38, 0.26) | -0.23 (-0.57, 0.1) | -0.14 (-0.47, 0.19) | 0.07 (-0.27, 0.41) | -0.22 (-0.59, 0.14) | -0.36 (-0.81, 0.09) | -0.3 (-0.66, 0.06) | -0.1 (-0.43, 0.24) | **-0.79 (-1.09, -0.48)** | 0.29 (-0.44, 1.03) | -0.03 (-0.38, 0.31) | -0.28 (-0.61, 0.05) | -0.14 (-0.46, 0.17) | -0.16 (-0.48, 0.16) |
| 0.21 (-0.15,0.57) | ALO | **0.66 (0.44, 0.89)** | 0.15 (-0.08,0.37) | -0.08 (-0.48,0.32) | -0.09 (-0.33, 0.15) | 0.11 (-0.11, 0.34) | **0.56 (0.27, 0.84)** | 0.23 (0, 0.45) | **0.66 (0.34, 0.98)** | 0.71 (-0.03, 1.46) | 0.13 (-0.1, 0.36) | 0.16 (-0.05, 0.36) | -0.02 (-0.25, 0.21) | 0.07 (-0.16, 0.3) | **0.29 (0.05, 0.52)** | -0.01 (-0.28, 0.26) | -0.14 (-0.52, 0.24) | -0.09 (-0.36, 0.18) | 0.12 (-0.08, 0.32) | **-0.57 (-0.76,-0.38)** | 0.51 (-0.19, 1.21) | 0.18 (-0.06, 0.42) | -0.07 (-0.29, 0.16) | 0.07 (-0.13, 0.27) | 0.05 (-0.16, 0.26) |
| -0.45 (-0.82,-0.08) | -0.66 (-0.89,-0.44) | ALO/PIO | **-0.52 (-0.76,-0.28)** | **-0.74 (-1.15,-0.33)** | **-0.76 (-1.01, -0.5)** | **-0.55 (-0.79, -0.31)** | -0.11 (-0.4, 0.19) | **-0.44 (-0.68,-0.19)** | -0.01 (-0.34, 0.33) | 0.05 (-0.7, 0.8) | **-0.53 (-0.78, -0.29)** | **-0.51 (-0.73, -0.29)** | **-0.69 (-0.93, -0.44)** | **-0.59 (-0.84, -0.35)** | **-0.38 (-0.63,-0.13)** | **-0.67 (-0.96,-0.39)** | **-0.81 (-1.2,-0.42)** | **-0.75 (-1.03,-0.47)** | **-0.55 (-0.75,-0.34)** | **-1.24 (-1.45,-1.02)** | -0.16 (-0.86, 0.55) | **-0.49 (-0.74, -0.23)** | **-0.73 (-0.97,-0.49)** | **-0.6 (-0.81,-0.38)** | **-0.61 (-0.84,-0.38)** |
| 0.07 (-0.26,0.4) | -0.15 (-0.37,0.08) | 0.52 (0.28,0.76) | CANA | -0.22 (-0.59,0.15) | **-0.24 (-0.43, -0.05)** | -0.03 (-0.2, 0.13) | **0.41 (0.17, 0.65)** | 0.08 (-0.09, 0.25) | **0.51 (0.23, 0.79)** | 0.57 (-0.17, 1.3) | -0.02 (-0.2, 0.17) | 0.01 (-0.12, 0.14) | -0.17 (-0.34, 0.01) | -0.08 (-0.25, 0.09) | 0.14 (-0.04, 0.32) | -0.16 (-0.39, 0.07) | -0.29 (-0.64, 0.06) | **-0.24 (-0.46, -0.01)** | -0.03 (-0.21, 0.15) | **-0.72 (-0.85, -0.59)** | 0.36 (-0.32, 1.04) | 0.03 (-0.16, 0.22) | **-0.21 (-0.38,-0.05)** | -0.08 (-0.2, 0.05) | -0.09 (-0.24, 0.06) |
| 0.29 (-0.18,0.76) | 0.08 (-0.32,0.48) | 0.74 (0.33,1.15) | 0.22 (-0.15,0.59) | COL | -0.02 (-0.4, 0.36) | 0.19 (-0.18, 0.56) | **0.63 (0.22, 1.05)** | 0.3 (-0.07, 0.68) | **0.74 (0.3, 1.17)** | 0.79 (-0.01, 1.59) | 0.21 (-0.16, 0.57) | 0.23 (-0.13, 0.59) | 0.05 (-0.32, 0.43) | 0.15 (-0.23, 0.52) | 0.36 (-0.01, 0.74) | 0.07 (-0.34, 0.47) | -0.07 (-0.55, 0.42) | -0.01 (-0.41, 0.38) | 0.19 (-0.18, 0.57) | **-0.5 (-0.85,-0.14)** | 0.58 (-0.18, 1.34) | 0.25 (-0.1, 0.61) | 0.01 (-0.36, 0.38) | 0.15 (-0.21, 0.5) | 0.13 (-0.23, 0.49) |
| 0.31 (-0.03,0.65) | 0.09 (-0.15,0.33) | 0.76 (0.5,1.01) | 0.24 (0.05,0.43) | 0.02 (-0.36,0.4) | DAPA | **0.21 (0.02, 0.39)** | **0.65 (0.39, 0.91)** | **0.32 (0.13, 0.51)** | **0.75 (0.46, 1.05)** | **0.81 (0.07, 1.54)** | **0.22 (0.02, 0.43)** | **0.25 (0.08, 0.42)** | 0.07 (-0.09, 0.23) | 0.16 (-0.03, 0.36) | **0.38 (0.18, 0.58)** | 0.08 (-0.16, 0.33) | -0.05 (-0.41, 0.31) | 0 (-0.24, 0.24) | **0.21 (0.01, 0.41)** | **-0.48 (-0.62,-0.33)** | 0.6 (-0.09, 1.29) | **0.27 (0.07,0.48)** | 0.03 (-0.15, 0.2) | **0.16 (0.01, 0.32)** | 0.15 (-0.03, 0.32) |
| 0.1 (-0.23,0.43) | -0.11 (-0.34,0.11) | 0.55 (0.31,0.79) | 0.03 (-0.13,0.2) | -0.19 (-0.56,0.18) | -0.21 (-0.39,-0.02) | EMPA | **0.44 (0.24, 0.64)** | 0.11 (-0.06, 0.28) | **0.54 (0.26, 0.83)** | 0.6 (-0.13, 1.33) | 0.02 (-0.17, 0.2) | 0.04 (-0.08, 0.16) | -0.14 (-0.31, 0.04) | -0.04 (-0.19, 0.1) | 0.17 (-0.01, 0.35) | -0.12 (-0.35, 0.1) | -0.26 (-0.61, 0.09) | -0.2 (-0.43, 0.02) | 0.0 (-0.17, 0.18) | **-0.69 (-0.81,-0.57)** | 0.39 (-0.29, 1.07) | 0.06 (-0.12, 0.25) | **-0.18 (-0.34,-0.02)** | -0.05 (-0.17, 0.08) | -0.06 (-0.21, 0.08) |
| -0.34 (-0.72,0.03) | -0.56 (-0.84,-0.27) | 0.11 (-0.19,0.4) | -0.41 (-0.65,-0.17) | -0.63 (-1.05,-0.22) | -0.65 (-0.91,-0.39) | -0.44 (-0.64,-0.24) | EMPA/LINA | **-0.33 (-0.58,-0.09)** | 0.1 (-0.23, 0.44) | 0.16 (-0.6, 0.91) | **-0.43 (-0.68,-0.17)** | **-0.4 (-0.62,-0.19)** | **-0.58 (-0.83,-0.33)** | **-0.49 (-0.69,-0.28)** | **-0.27 (-0.52,-0.02)** | **-0.57 (-0.86,-0.28)** | **-0.7 (-1.09,-0.31)** | **-0.65 (-0.93,-0.36)** | **-0.44 (-0.69,-0.19)** | **-1.13 (-1.34,-0.92)** | -0.05 (-0.76, 0.65) | **-0.38 (-0.64, -0.12)** | **-0.62 (-0.86, -0.38)** | **-0.49 (-0.71, -0.27)** | **-0.5 (-0.73,-0.28)** |
| -0.01 (-0.34,0.32) | -0.23 (-0.45,0) | 0.44 (0.19,0.68) | -0.08 (-0.25,0.09) | -0.3 (-0.68,0.07) | -0.32 (-0.51,-0.13) | -0.11 (-0.28,0.06) | 0.33 (0.09,0.58) | EXEN | **0.43 (0.14, 0.72)** | 0.49 (-0.24, 1.22) | -0.1 (-0.29, 0.09) | -0.07 (-0.21, 0.07) | **-0.25 (-0.43,-0.07)** | -0.15 (-0.33, 0.02) | 0.06 (-0.13, 0.25) | **-0.24 (-0.42,-0.05)** | **-0.37 (-0.72,-0.02)** | **-0.32 (-0.54,-0.09)** | -0.11 (-0.29, 0.07) | **-0.8 (-0.92,-0.67)** | 0.28 (-0.4, 0.96) | -0.05 (-0.24, 0.15) | **-0.29 (-0.46,-0.12)** | **-0.16 (-0.3,-0.02)** | **-0.17 (-0.33,-0.02)** |
| -0.45 (-0.85,-0.04) | -0.66 (-0.98,-0.34) | 0.01 (-0.33,0.34) | -0.51 (-0.79,-0.23) | -0.74 (-1.17,-0.3) | -0.75 (-1.05,-0.46) | -0.54 (-0.83,-0.26) | -0.1 (-0.44,0.23) | -0.43 (-0.72,-0.14) | GLAR | 0.05 (-0.71, 0.82) | **-0.53 (-0.82,-0.23)** | **-0.5 (-0.77,-0.23)** | **-0.68 (-0.97,-0.39)** | **-0.59 (-0.88,-0.3)** | **-0.37 (-0.66,-0.08)** | **-0.67 (-1, -0.34)** | **-0.8 (-1.22,-0.38)** | **-0.75 (-1.07,-0.43)** | **-0.54 (-0.83,-0.25)** | **-1.23 (-1.49,-0.97)** | -0.15 (-0.87, 0.57) | **-0.48 (-0.77,-0.19)** | **-0.72 (-1, -0.44)** | **-0.59 (-0.84,-0.34)** | **-0.61 (-0.88,-0.33)** |
| -0.5 (-1.15,0.15) | -0.71 (-1.46,0.03) | -0.05 (-0.8,0.7) | -0.57 (-1.3,0.17) | -0.79 (-1.59,0.01) | -0.81 (-1.54,-0.07) | -0.6 (-1.33,0.13) | -0.16 (-0.91,0.6) | -0.49 (-1.22,0.24) | -0.05 (-0.82,0.71) | GLIB | -0.58 (-1.32, 0.15) | -0.56 (-1.28, 0.17) | -0.73 (-1.47, 0) | -0.64 (-1.37, 0.09) | -0.43 (-1.16, 0.31) | -0.72 (-1.47, 0.02) | **-0.86 (-1.65,-0.06)** | **-0.8 (-1.55,-0.06)** | -0.6 (-1.33, 0.14) | **-1.29 (-2.01,-0.56)** | -0.21 (-1.19, 0.78) | -0.53 (-1.27, 0.2) | **-0.78 (-1.51,-0.05)** | -0.64 (-1.37, 0.08) | -0.66 (-1.39, 0.07) |
| 0.08 (-0.26,0.42) | -0.13 (-0.36,0.1) | 0.53 (0.29,0.78) | 0.02 (-0.17,0.2) | -0.21 (-0.57,0.16) | -0.22 (-0.43,-0.02) | -0.02 (-0.2,0.17) | 0.43 (0.17,0.68) | 0.1 (-0.09,0.29) | 0.53 (0.23,0.82) | 0.58 (-0.15,1.32) | GLIC | 0.03 (-0.13, 0.19) | -0.15 (-0.35, 0.04) | -0.06 (-0.25, 0.13) | 0.16 (-0.04, 0.36) | -0.14 (-0.38, 0.1) | -0.27 (-0.63, 0.09) | **-0.22 (-0.42,-0.02)** | -0.01 (-0.18, 0.15) | **-0.7 (-0.85,-0.56)** | 0.38 (-0.31, 1.06) | 0.05 (-0.11, 0.21) | **-0.19 (-0.38,-0.01)** | -0.06 (-0.21, 0.09) | -0.08 (-0.23, 0.08) |
| 0.06 (-0.26,0.38) | -0.16 (-0.36,0.05) | 0.51 (0.29,0.73) | -0.01 (-0.14,0.12) | -0.23 (-0.59,0.13) | -0.25 (-0.42,-0.08) | -0.04 (-0.16,0.08) | 0.4 (0.19,0.62) | 0.07 (-0.07,0.21) | 0.5 (0.23,0.77) | 0.56 (-0.17,1.28) | -0.03 (-0.19,0.13) | GLIM | **-0.18 (-0.33,-0.02)** | -0.08 (-0.21, 0.04) | 0.13 (-0.02, 0.28) | -0.17 (-0.37, 0.04) | -0.3 (-0.64, 0.04) | **-0.25 (-0.45,-0.04)** | -0.04 (-0.18, 0.11) | **-0.73 (-0.82,-0.64)** | 0.35 (-0.33, 1.03) | 0.02 (-0.14, 0.19) | **-0.22 (-0.36,-0.08)** | -0.09 (-0.18, 0.01) | -0.1 (-0.21, 0) |
| 0.23 (-0.1,0.57) | 0.02 (-0.21,0.25) | 0.69 (0.44,0.93) | 0.17 (-0.01,0.34) | -0.05 (-0.43,0.32) | -0.07 (-0.23,0.09) | 0.14 (-0.04,0.31) | 0.58 (0.33,0.83) | 0.25 (0.07,0.43) | 0.68 (0.39,0.97) | 0.73 (0,1.47) | 0.15 (-0.04,0.35) | 0.18 (0.02,0.33) | GLIP | 0.09 (-0.09, 0.27) | **0.31 (0.12, 0.5)** | 0.01 (-0.22, 0.25) | -0.12 (-0.48, 0.23) | -0.07 (-0.3, 0.16) | 0.14 (-0.05, 0.32) | **-0.55 (-0.68,-0.42)** | 0.53 (-0.16, 1.21) | 0.2 (0, 0.4) | -0.04 (-0.19, 0.1) | 0.09 (-0.04, 0.22) | 0.07 (-0.09, 0.24) |
| 0.14 (-0.19,0.47) | -0.07 (-0.3,0.16) | 0.59 (0.35,0.84) | 0.08 (-0.09,0.25) | -0.15 (-0.52,0.23) | -0.16 (-0.36,0.03) | 0.04 (-0.1,0.19) | 0.49 (0.28,0.69) | 0.15 (-0.02,0.33) | 0.59 (0.3,0.88) | 0.64 (-0.09,1.37) | 0.06 (-0.13,0.25) | 0.08 (-0.04,0.21) | -0.09 (-0.27,0.09) | LINA | **0.22 (0.03, 0.4)** | -0.08 (-0.31, 0.15) | -0.21 (-0.57, 0.14) | -0.16 (-0.39, 0.07) | 0.05 (-0.13, 0.23) | **-0.64 (-0.77,-0.52)** | 0.44 (-0.25, 1.12) | 0.11 (-0.09, 0.3) | -0.14 (-0.31, 0.03) | 0 (-0.14, 0.14) | -0.02 (-0.17, 0.13) |
| -0.07 (-0.41,0.27) | -0.29 (-0.52,-0.05) | 0.38 (0.13,0.63) | -0.14 (-0.32,0.04) | -0.36 (-0.74,0.01) | -0.38 (-0.58,-0.18) | -0.17 (-0.35,0.01) | 0.27 (0.02,0.52) | -0.06 (-0.25,0.13) | 0.37 (0.08,0.66) | 0.43 (-0.31,1.16) | -0.16 (-0.36,0.04) | -0.13 (-0.28,0.02) | -0.31 (-0.5,-0.12) | -0.22 (-0.4,-0.03) | LIRA | **-0.3 (-0.54,-0.05)** | **-0.43 (-0.79,-0.07)** | **-0.38 (-0.61,-0.14)** | -0.17 (-0.36, 0.02) | **-0.86 (-1,-0.71)** | 0.22 (-0.47, 0.91) | -0.11 (-0.31, 0.09) | **-0.35 (-0.53,-0.17)** | **-0.22 (-0.36, -0.08)** | **-0.23 (-0.4, -0.07)** |
| 0.22 (-0.14,0.59) | 0.01 (-0.26,0.28) | 0.67 (0.39,0.96) | 0.16 (-0.07,0.39) | -0.07 (-0.47,0.34) | -0.08 (-0.33,0.16) | 0.12 (-0.1,0.35) | 0.57 (0.28,0.86) | 0.24 (0.05,0.42) | 0.67 (0.34,1) | 0.72 (-0.02,1.47) | 0.14 (-0.1,0.38) | 0.17 (-0.04,0.37) | -0.01 (-0.25,0.22) | 0.08 (-0.15,0.31) | 0.3 (0.05,0.54) | LIX | -0.13 (-0.52, 0.25) | -0.08 (-0.35, 0.19) | 0.13 (-0.11, 0.37) | **-0.56 (-0.76,-0.37)** | 0.52 (-0.18, 1.22) | 0.19 (-0.06, 0.43) | -0.06 (-0.28, 0.17) | 0.08 (-0.13, 0.29) | 0.06 (-0.15, 0.28) |
| 0.36 (-0.09,0.81) | 0.14 (-0.24,0.52) | 0.81 (0.42,1.2) | 0.29 (-0.06,0.64) | 0.07 (-0.42,0.55) | 0.05 (-0.31,0.41) | 0.26 (-0.09,0.61) | 0.7 (0.31,1.09) | 0.37 (0.02,0.72) | 0.8 (0.38,1.22) | 0.86 (0.06,1.65) | 0.27 (-0.09,0.63) | 0.3 (-0.04,0.64) | 0.12 (-0.23,0.48) | 0.21 (-0.14,0.57) | 0.43 (0.07,0.79) | 0.13 (-0.25,0.52) | MIG | 0.05 (-0.33, 0.43) | 0.26 (-0.1, 0.62) | **-0.43 (-0.76,-0.1)** | 0.65 (-0.1, 1.4) | 0.32 (-0.04, 0.68) | 0.08 (-0.27, 0.42) | 0.21 (-0.12, 0.55) | 0.2 (-0.15, 0.54) |
| 0.3 (-0.06,0.66) | 0.09 (-0.18,0.36) | 0.75 (0.47,1.03) | 0.24 (0.01,0.46) | 0.01 (-0.38,0.41) | 0 (-0.24,0.24) | 0.2 (-0.02,0.43) | 0.65 (0.36,0.93) | 0.32 (0.09,0.54) | 0.75 (0.43,1.07) | 0.8 (0.06,1.55) | 0.22 (0.02,0.42) | 0.25 (0.04,0.45) | 0.07 (-0.16,0.3) | 0.16 (-0.07,0.39) | 0.38 (0.14,0.61) | 0.08 (-0.19,0.35) | -0.05 (-0.43,0.33) | NAT | 0.21 (-0.02, 0.43) | **-0.48 (-0.67,-0.29)** | 0.6 (-0.1, 1.3) | **0.27 (0.04, 0.49)** | 0.02 (-0.2, 0.25) | 0.16 (-0.04, 0.36) | 0.14 (-0.07, 0.35) |
| 0.1 (-0.24,0.43) | -0.12 (-0.32,0.08) | 0.55 (0.34,0.75) | 0.03 (-0.15,0.21) | -0.19 (-0.57,0.18) | -0.21 (-0.41,-0.01) | 0 (-0.18,0.17) | 0.44 (0.19,0.69) | 0.11 (-0.07,0.29) | 0.54 (0.25,0.83) | 0.6 (-0.14,1.33) | 0.01 (-0.15,0.18) | 0.04 (-0.11,0.18) | -0.14 (-0.32,0.05) | -0.05 (-0.23,0.13) | 0.17 (-0.02,0.36) | -0.13 (-0.37,0.11) | -0.26 (-0.62,0.1) | -0.21 (-0.43,0.02) | PIO | **-0.69 (-0.83,-0.55)** | 0.39 (-0.3, 1.08) | 0.06 (-0.13, 0.25) | **-0.18 (-0.36,-0.01)** | -0.05 (-0.19, 0.09) | -0.06 (-0.22, 0.09) |
| 0.79 (0.48,1.09) | 0.57 (0.38,0.76) | 1.24 (1.02,1.45) | 0.72 (0.59,0.85) | 0.5 (0.14,0.85) | 0.48 (0.33,0.62) | 0.69 (0.57,0.81) | 1.13 (0.92,1.34) | 0.8 (0.67,0.92) | 1.23 (0.97,1.49) | 1.29 (0.56,2.01) | 0.7 (0.56,0.85) | 0.73 (0.64,0.82) | 0.55 (0.42,0.68) | 0.64 (0.52,0.77) | 0.86 (0.71,1) | 0.56 (0.37,0.76) | 0.43 (0.1,0.76) | 0.48 (0.29,0.67) | 0.69 (0.55,0.83) | PLC | **1.08 (0.41, 1.75)** | **0.75 (0.6, 0.9)** | **0.51 (0.39, 0.62)** | **0.64 (0.57, 0.71)** | **0.63 (0.53, 0.72)** |
| -0.29 (-1.03,0.44) | -0.51 (-1.21,0.19) | 0.16 (-0.55,0.86) | -0.36 (-1.04,0.32) | -0.58 (-1.34,0.18) | -0.6 (-1.29,0.09) | -0.39 (-1.07,0.29) | 0.05 (-0.65,0.76) | -0.28 (-0.96,0.4) | 0.15 (-0.57,0.87) | 0.21 (-0.78,1.19) | -0.38 (-1.06,0.31) | -0.35 (-1.03,0.33) | -0.53 (-1.21,0.16) | -0.44 (-1.12,0.25) | -0.22 (-0.91,0.47) | -0.52 (-1.22,0.18) | -0.65 (-1.4,0.1) | -0.6 (-1.3,0.1) | -0.39 (-1.08,0.3) | -1.08 (-1.75,-0.41) | REP | -0.33 (-1.02, 0.36) | -0.57 (-1.25, 0.11) | -0.44 (-1.11, 0.24) | -0.45 (-1.13, 0.22) |
| 0.03 (-0.31,0.38) | -0.18 (-0.42,0.06) | 0.49 (0.23,0.74) | -0.03 (-0.22,0.16) | -0.25 (-0.61,0.1) | -0.27 (-0.48,-0.07) | -0.06 (-0.25,0.12) | 0.38 (0.12,0.64) | 0.05 (-0.15,0.24) | 0.48 (0.19,0.77) | 0.53 (-0.2,1.27) | -0.05 (-0.21,0.11) | -0.02 (-0.19,0.14) | -0.2 (-0.4,0) | -0.11 (-0.3,0.09) | 0.11 (-0.09,0.31) | -0.19 (-0.43,0.06) | -0.32 (-0.68,0.04) | -0.27 (-0.49,-0.04) | -0.06 (-0.25,0.13) | -0.75 (-0.9,-0.6) | 0.33 (-0.36,1.02) | ROSI | **-0.24 (-0.43,-0.06)** | -0.11 (-0.26, 0.04) | -0.13 (-0.29, 0.04) |
| 0.28 (-0.05,0.61) | 0.07 (-0.16,0.29) | 0.73 (0.49,0.97) | 0.21 (0.05,0.38) | -0.01 (-0.38,0.36) | -0.03 (-0.2,0.15) | 0.18 (0.02,0.34) | 0.62 (0.38,0.86) | 0.29 (0.12,0.46) | 0.72 (0.44,1) | 0.78 (0.05,1.51) | 0.19 (0.01,0.38) | 0.22 (0.08,0.36) | 0.04 (-0.1,0.19) | 0.14 (-0.03,0.31) | 0.35 (0.17,0.53) | 0.06 (-0.17,0.28) | -0.08 (-0.42,0.27) | -0.02 (-0.25,0.2) | 0.18 (0.01,0.36) | -0.51 (-0.62,-0.39) | 0.57 (-0.11,1.25) | 0.24 (0.06,0.43) | SAX | **0.13 (0.01,0.25)** | 0.12 (-0.03, 0.27) |
| 0.14 (-0.17,0.46) | -0.07 (-0.27,0.13) | 0.6 (0.38,0.81) | 0.08 (-0.05,0.2) | -0.15 (-0.5,0.21) | -0.16 (-0.32,-0.01) | 0.05 (-0.08,0.17) | 0.49 (0.27,0.71) | 0.16 (0.02,0.3) | 0.59 (0.34,0.84) | 0.64 (-0.08,1.37) | 0.06 (-0.09,0.21) | 0.09 (-0.01,0.18) | -0.09 (-0.22,0.04) | 0 (-0.14,0.14) | 0.22 (0.08,0.36) | -0.08 (-0.29,0.13) | -0.21 (-0.55,0.12) | -0.16 (-0.36,0.04) | 0.05 (-0.09,0.19) | -0.64 (-0.71,-0.57) | 0.44 (-0.24,1.11) | 0.11 (-0.04,0.26) | -0.13 (-0.25,-0.01) | SITA | -0.02 (-0.13,0.1) |
| 0.16 (-0.16,0.48) | -0.05 (-0.26,0.16) | 0.61 (0.38,0.84) | 0.09 (-0.06,0.24) | -0.13 (-0.49,0.23) | -0.15 (-0.32,0.03) | 0.06 (-0.08,0.21) | 0.5 (0.28,0.73) | 0.17 (0.02,0.33) | 0.61 (0.33,0.88) | 0.66 (-0.07,1.39) | 0.08 (-0.08,0.23) | 0.1 (0,0.21) | -0.07 (-0.24,0.09) | 0.02 (-0.13,0.17) | 0.23 (0.07,0.4) | -0.06 (-0.28,0.15) | -0.2 (-0.54,0.15) | -0.14 (-0.35,0.07) | 0.06 (-0.09,0.22) | -0.63 (-0.72,-0.53) | 0.45 (-0.22,1.13) | 0.13 (-0.04,0.29) | -0.12 (-0.27,0.03) | 0.02 (-0.1,0.13) | VILDA |
