# Supplementary material for: Comparative Efficacy and Safety of Antidiabetic Drug Regimens Added to Metformin Monotherapy in Patients with Type 2 Diabetes: A Network Meta-Analysis
Source: PLoS One. 2015 Apr 28;10(4):e0125879. doi: 10.1371/journal.pone.0125879 (PMC4412636; doi:10.1371/journal.pone.0125879)
Supplement: S4 Fig — Therapies are reported in alphabetical order. Results are reported in WMD, kg (95% CI). Results for changes in weight on the top portion of the matrix represent changes in the row-defining treatment vs. those in the column-defining treatment (referent). For changes in weight, negative values favor the first agent in alphabetical order. Statistically significant results are bolded. Clinically superior results are underlined. Sodium glucose co-transporter-2 (SGLT-2) inhibitors are highlighted. The results on the bottom portion of the matrix represent the reciprocal of the top portion. ACA = acarbose; ALO = alogliptin; ALO/PIO = alogliptin/pioglitazone; CANA = canagliflozin; COL = colesevelam; DAPA = dapagliflozin; EMPA = empagliflozin; EMPA/LINA = empagliflozin/linagliptin; EXEN = exenatide; GLAR = glargine; GLIB = glibenclamide; GLIC = gliclazide; GLIM = glimepiride; GLIP = glipizide; LINA = linagliptin; LIRA = liraglutide; LIX = lixisenatide; MIG = miglitol; NAT = nateglinide; PIO = pioglitazone; PLC = placebo; REP = repaglinide; ROSI = rosiglitazone; SAX = saxagliptin; SITA = sitagliptin; VILDA = vildagliptin. (PDF) [file pone.0125879.s007.pdf]

Figure S4. Network Meta-Analysis Results of the Effect of Antidiabetic Therapies on Change in Body Weight From Baseline

ACA	-2.49 (-3.83,-1.15)	-0.19 (-1.42, 1.03)	2.05 (0.9, 3.21)	-0.99 (-2.45, 0.46)	2.07 (0.87, 3.28)	1.97 (0.6, 3.34)	1.98 (0.84, 3.11)	2.16 (0.79, 3.53)	-2.3 (-3.7, -0.9)	-1.29 (-2.61, 0.03)	-2.29 (-3.39,-1.18)	-2.54 (-3.73,-1.35)	-1.83 (-3.23,-0.43)	-0.06 (-1.26, 1.14)	1.5 (0.33, 2.67)	1.05 (-0.33, 2.43)	1.7 (0.12, 3.28)	-0.7 (-2.02, 0.62)	-2.16 (-3.45, -0.87)	-0.1 (-1.15, 0.95)	-3.37 (-5.23,-1.51)	-2.25 (-3.42,-1.09)	-0.16 (-1.33, 1.0)	-0.31 (-1.41, 0.78)	-0.14 (-1.26, 0.99)
2.49 (1.15,3.83)	ALO/PIO	2.3 (1.48, 3.11)	4.54 (3.59, 5.5)	1.5 (0.19, 2.8)	4.56 (3.54, 5.59)	4.46 (3.26, 5.67)	4.46 (3.53, 5.4)	4.65 (3.43, 5.87)	0.19 (-1.75, 2.13)	1.2 (0.05, 2.35)	0.2 (-0.67, 1.08)	-0.05 (-1.05, 0.96)	0.66 (-0.59, 1.9)	2.43 (1.43, 3.43)	3.99 (3.02, 4.96)	3.54 (2.32, 4.77)	4.19 (2.75, 5.63)	1.79 (0.63, 2.95)	0.33 (-0.46, 1.12)	2.39 (1.55, 3.23)	-0.88 (-2.63, 0.87)	0.24 (-0.74, 1.21)	2.33 (1.35, 3.3)	2.18 (1.29, 3.06)	2.35 (1.43, 3.27)
0.19 (-1.03,1.42)	-2.3 (-3.11,-1.48)	ALO	2.25 (1.46, 3.03)	-0.8 (-1.98, 0.38)	2.27 (1.4, 3.14)	2.17 (1.09, 3.24)	2.17 (1.41, 2.93)	2.35 (1.27, 3.44)	-2.11 (-3.96,-0.25)	-1.1 (-2.11,-0.08)	-2.1 (-2.79,-1.4)	-2.34 (-3.18,-1.51)	-1.64 (-2.76,-0.52)	0.13 (-0.71, 0.98)	1.69 (0.89, 2.5)	1.25 (0.15, 2.34)	1.89 (0.56, 3.23)	-0.51 (-1.53, 0.51)	-1.97 (-2.75,-1.19)	0.09 (-0.53, 0.72)	-3.18 (-4.84,-1.52)	-2.06 (-2.86,-1.26)	0.03 (-0.77, 0.84)	-0.12 (-0.82, 0.58)	0.06 (-0.69, 0.8)
-2.05 (-3.21,-0.9)	-4.54 (-5.5,-3.59)	-2.25 (-3.03,-1.46)	CANA	-3.05 (-4.12,-1.97)	0.02 (-0.74, 0.78)	-0.08 (-1.05, 0.89)	-0.08 (-0.69, 0.53)	0.11 (-0.9, 1.12)	-4.35 (-6.16,-2.54)	-3.34 (-4.26,-2.43)	-4.34 (-4.84,-3.84)	-4.59 (-5.31,-3.87)	-3.89 (-4.89,-2.88)	-2.11 (-2.82,-1.4)	-0.55 (-1.2, 0.09)	-1.0 (-2.02, 0.02)	-0.35 (-1.62, 0.92)	-2.75 (-3.69,-1.82)	-4.21 (-5.08,-3.34)	-2.15 (-2.63,-1.67)	-5.42 (-7.03,-3.81)	-4.31 (-4.97,-3.64)	-2.21 (-2.91,-1.52)	-2.37 (-2.85,-1.88)	-2.19 (-2.79,-1.59)
0.99 (-0.46,2.45)	-1.5 (-2.8,-0.19)	0.8 (-0.38,1.98)	3.05 (1.97,4.12)	COL	3.07 (1.9, 4.23)	2.97 (1.64, 4.29)	2.97 (1.89, 4.05)	3.15 (1.81, 4.49)	-1.31 (-3.32, 0.71)	-0.3 (-1.54, 0.94)	-1.3 (-2.33,-0.26)	-1.54 (-2.67, -0.41)	-0.84 (-2.15, 0.47)	0.93 (-0.21, 2.08)	2.49 (1.41, 3.58)	2.05 (0.7, 3.39)	2.69 (1.15, 4.24)	0.29 (-0.99, 1.58)	-1.17 (-2.41, 0.08)	0.89 (-0.11, 1.9)	-2.38 (-4.21, -0.54)	-1.26 (-2.29, -0.24)	0.83 (-0.29, 1.95)	0.68 (-0.3, 1.66)	0.86 (-0.21, 1.92)
-2.07 (-3.28,-0.87)	-4.56 (-5.59,-3.54)	-2.27 (-3.14,-1.4)	-0.02 (-0.78,0.74)	-3.07 (-4.23,-1.9)	DAPA	-0.1 (-1.17, 0.96)	-0.1 (-0.84, 0.64)	0.09 (-0.98, 1.16)	-4.37 (-6.22,-2.53)	-3.37 (-4.36,-2.37)	-4.36 (-5.05,-3.67)	-4.61 (-5.24,-3.98)	-3.91 (-5.0,-2.81)	-2.13 (-2.96,-1.3)	-0.57 (-1.36, 0.21)	-1.02 (-2.1, 0.06)	-0.37 (-1.69, 0.95)	-2.77 (-3.78,-1.77)	-4.24 (-5.2,-3.27)	-2.17 (-2.78,-1.57)	-5.44 (-7.09,-3.79)	-4.33 (-5.11,-3.55)	-2.24 (-2.96,-1.51)	-2.39 (-3.05,-1.73)	-2.21 (-2.93,-1.49)
-1.97 (-3.34,-0.6)	-4.46 (-5.67,-3.26)	-2.17 (-3.24,-1.09)	0.08 (-0.89,1.05)	-2.97 (-4.29,-1.64)	0.1 (-0.96,1.17)	EMPA/LINA	0.0 (-0.82, 0.82)	0.19 (-1.06, 1.44)	-4.27 (-6.23, -2.32)	-3.26 (-4.44,-2.09)	-4.26 (-5.14,-3.38)	-4.51 (-5.55,-3.47)	-3.8 (-5.07,-2.54)	-2.03 (-2.89,-1.17)	-0.47 (-1.46, 0.52)	-0.92 (-2.18, 0.34)	-0.27 (-1.74, 1.2)	-2.67 (-3.86,-1.48)	-4.13 (-5.27,-3.0)	-2.07 (-2.95,-1.19)	-5.34 (-7.11, -3.57)	-4.23 (-5.23,-3.22)	-2.13 (-3.15,-1.12)	-2.28 (-3.19,-1.38)	-2.11 (-3.06,-1.16)
-1.98 (-3.11,-0.84)	-4.46 (-5.4,-3.53)	-2.17 (-2.93,-1.41)	0.08 (-0.53,0.69)	-2.97 (-4.05,-1.89)	0.1 (-0.64,0.84)	0 (-0.82,0.82)	EMPA	0.19 (-0.81,1.18)	-4.28 (-6.08,-2.47)	-3.27 (-4.16,-2.37)	-4.26 (-4.72,-3.81)	-4.51 (-5.21,-3.81)	-3.81 (-4.81,-2.8)	-2.03 (-2.64,-1.43)	-0.47 (-1.11, 0.16)	-0.92 (-1.92, 0.08)	-0.28 (-1.53, 0.98)	-2.68 (-3.59,-1.76)	-4.14 (-4.98,-3.29)	-2.08 (-2.52,-1.63)	-5.35 (-6.94,-3.75)	-4.23 (-4.88,-3.58)	-2.14 (-2.81,-1.47)	-2.29 (-2.78,-1.8)	-2.11 (-2.68,-1.54)
-2.16 (-3.53,-0.79)	-4.65 (-5.87,-3.43)	-2.35 (-3.44,-1.27)	-0.11 (-1.12,0.9)	-3.15 (-4.49,-1.81)	-0.09 (-1.16,0.98)	-0.19 (-1.44,1.06)	-0.19 (-1.18,0.81)	EXEN	-4.46 (-6.42, -2.5)	-3.45 (-4.65, -2.26)	-4.45 (-5.4, -3.5)	-4.7 (-5.75,-3.65)	-3.99 (-5.28,-2.71)	-2.22 (-3.28,-1.16)	-0.66 (-1.69, 0.37)	-1.11 (-1.9, -0.31)	-0.46 (-1.93, 1.01)	-2.86 (-4.06,-1.66)	-4.32 (-5.48,-3.16)	-2.26 (-3.15,-1.37)	-5.53 (-7.3, -3.76)	-4.41 (-5.43, -3.4)	-2.32 (-3.34, -1.3)	-2.47 (-3.41, -1.53)	-2.3 (-3.27, -1.32)
2.3 (0.9, 3.7)	-0.19 (-2.13,1.75)	2.11 (0.25,3.96)	4.35 (2.54,6.16)	1.31 (-0.71,3.32)	4.37 (2.53,6.22)	4.27 (2.32,6.23)	4.28 (2.47,6.08)	4.46 (2.5,6.42)	GLIB	1.01 (-0.91, 2.93)	0.01 (-1.77, 1.79)	-0.24 (-2.07, 1.6)	0.47 (-1.51, 2.45)	2.24 (0.4, 4.08)	3.8 (1.98, 5.62)	3.35 (1.39, 5.32)	4.0 (1.9, 6.1)	1.6 (-0.32, 3.52)	0.14 (-1.76, 2.04)	2.2 (0.45, 3.95)	-1.07 (-3.4, 1.26)	0.05 (-1.77, 1.86)	2.14 (0.32, 3.96)	1.99 (0.21, 3.76)	2.16 (0.37, 3.96)
1.29 (-0.03,2.61)	-1.2 (-2.35,-0.05)	1.1 (0.08,2.11)	3.34 (2.43,4.26)	0.3 (-0.94,1.54)	3.37 (2.37,4.36)	3.26 (2.09,4.44)	3.27 (2.37,4.16)	3.45 (2.26,4.65)	-1.01 (-2.93,0.91)	GLIC	-1.0 (-1.84,-0.16)	-1.25 (-2.21,-0.28)	-0.54 (-1.75, 0.67)	1.23 (0.26, 2.2)	2.79 (1.86, 3.72)	2.35 (1.14, 3.55)	2.99 (1.57, 4.41)	0.59 (-0.54, 1.72)	-0.87 (-1.96, 0.22)	1.19 (0.39, 1.99)	-2.08 (-3.81, -0.35)	-0.96 (-1.75, -0.17)	1.13 (0.19, 2.07)	0.98 (0.15, 1.81)	1.15 (0.37, 1.94)
2.29 (1.18,3.39)	-0.2 (-1.08,0.67)	2.1 (1.4,2.79)	4.34 (3.84,4.84)	1.3 (0.26,2.33)	4.36 (3.67,5.05)	4.26 (3.38,5.14)	4.26 (3.81,4.72)	4.45 (3.5,5.4)	-0.01 (-1.79,1.77)	1 (0.16,1.84)	GLIM	-0.25 (-0.89, 0.39)	0.46 (-0.5, 1.41)	2.23 (1.68, 2.78)	3.79 (3.26, 4.32)	3.34 (2.38, 4.3)	3.99 (2.76, 5.22)	1.59 (0.71, 2.46)	0.13 (-0.64, 0.9)	2.19 (1.84, 2.54)	-1.08 (-2.66, 0.49)	0.03 (-0.55, 0.62)	2.13 (1.52, 2.74)	1.98 (1.59, 2.36)	2.15 (1.7, 2.61)
2.54 (1.35,3.73)	0.05 (-0.96,1.05)	2.34 (1.51,3.18)	4.59 (3.87,5.31)	1.54 (0.41,2.67)	4.61 (3.98,5.24)	4.51 (3.47,5.55)	4.51 (3.81,5.21)	4.7 (3.65,5.75)	0.24 (-1.6,2.07)	1.25 (0.28,2.21)	0.25 (-0.39,0.89)	GLIP	0.71 (-0.35, 1.76)	2.48 (1.68, 3.27)	4.04 (3.3, 4.78)	3.59 (2.54, 4.65)	4.24 (2.94, 5.54)	1.84 (0.86, 2.81)	0.38 (-0.56, 1.31)	2.44 (1.88, 3.0)	-0.83 (-2.47, 0.8)	0.28 (-0.45, 1.02)	2.38 (1.78, 2.98)	2.23 (1.63, 2.82)	2.4 (1.72, 3.08)
1.83 (0.43,3.23)	-0.66 (-1.9,0.59)	1.64 (0.52,2.76)	3.89 (2.88,4.89)	0.84 (-0.47,2.15)	3.91 (2.81,5)	3.8 (2.54,5.07)	3.81 (2.8,4.81)	3.99 (2.71,5.28)	-0.47 (-2.45,1.51)	0.54 (-0.67,1.75)	-0.46 (-1.41,0.5)	-0.71 (-1.76,0.35)	GLAR	1.77 (0.7, 2.85)	3.33 (2.33, 4.34)	2.89 (1.59, 4.18)	3.53 (2.03, 5.03)	1.13 (-0.1, 2.36)	-0.33 (-1.51, 0.86)	1.73 (0.8, 2.66)	-1.54 (-3.33, 0.26)	-0.42 (-1.44, 0.6)	1.67 (0.62, 2.72)	1.52 (0.64, 2.4)	1.69 (0.69, 2.7)
0.06 (-1.14,1.26)	-2.43 (-3.43,-1.43)	-0.13 (-0.98,0.71)	2.11 (1.4,2.82)	-0.93 (-2.08,0.21)	2.13 (1.3,2.96)	2.03 (1.17,2.89)	2.03 (1.43,2.64)	2.22 (1.16,3.28)	-2.24 (-4.08,-0.4)	-1.23 (-2.2,-0.26)	-2.23 (-2.78,-1.68)	-2.48 (-3.27,-1.68)	-1.77 (-2.85,-0.7)	LINA	1.56 (0.83, 2.29)	1.11 (0.05, 2.18)	1.76 (0.45, 3.07)	-0.64 (-1.63, 0.35)	-2.1 (-3.02, -1.19)	-0.04 (-0.62, 0.54)	-3.31 (-4.95, -1.67)	-2.2 (-2.95,-1.44)	-0.1 (-0.87, 0.66)	-0.25 (-0.87, 0.37)	-0.08 (-0.75, 0.59)
-1.5 (-2.67,-0.33)	-3.99 (-4.96,-3.02)	-1.69 (-2.5,-0.89)	0.55 (-0.09,1.2)	-2.49 (-3.58,-1.41)	0.57 (-0.21,1.36)	0.47 (-0.52,1.46)	0.47 (-0.16,1.11)	0.66 (-0.37,1.69)	-3.8 (-5.62,-1.98)	-2.79 (-3.72,-1.86)	-3.79 (-4.32,-3.26)	-4.04 (-4.78,-3.3)	-3.33 (-4.34,-2.33)	-1.56 (-2.29,-0.83)	LIRA	-0.45 (-1.48,0.59)	0.2 (-1.09,1.48)	-2.2 (-3.16, -1.25)	-3.66 (-4.55, -2.77)	-1.6 (-2.12, -1.08)	-4.87 (-6.49, -3.25)	-3.75 (-4.44, -3.07)	-1.66 (-2.38, -0.95)	-1.81 (-2.31,-1.32)	-1.64 (-2.27, -1.01)
-1.05 (-2.43,0.33)	-3.54 (-4.77,-2.32)	-1.25 (-2.34,-0.15)	1 (-0.02,2.02)	-2.05 (-3.39,-0.7)	1.02 (-0.06,2.1)	0.92 (-0.34,2.18)	0.92 (-0.08,1.92)	1.11 (0.31,1.9)	-3.35 (-5.32,-1.39)	-2.35 (-3.55,-1.14)	-3.34 (-4.3,-2.38)	-3.59 (-4.65,-2.54)	-2.89 (-4.18,-1.59)	-1.11 (-2.18,-0.05)	0.45 (-0.59,1.48)	LIX	0.65 (-0.83, 2.12)	-1.75 (-2.96, -0.55)	-3.22 (-4.38,-2.05)	-1.15 (-2.05,-0.26)	-4.42 (-6.2, -2.65)	-3.31 (-4.34,-2.28)	-1.22 (-2.24,-0.19)	-1.37 (-2.32,-0.42)	-1.19 (-2.18,-0.21)
-1.7 (-3.28,-0.12)	-4.19 (-5.63,-2.75)	-1.89 (-3.23,-0.56)	0.35 (-0.92,1.62)	-2.69 (-4.24,-1.15)	0.37 (-0.95,1.69)	0.27 (-1.2,1.74)	0.28 (-0.98,1.53)	0.46 (-1.01,1.93)	-4 (-6.1,-1.9)	-2.99 (-4.41,-1.57)	-3.99 (-5.22,-2.76)	-4.24 (-5.54,-2.94)	-3.53 (-5.03,-2.03)	-1.76 (-3.07,-0.45)	-0.2 (-1.48,1.09)	-0.65 (-2.12,0.83)	MIG	-2.4 (-3.82,-0.98)	-3.86 (-5.26,-2.47)	-1.8 (-2.98,-0.62)	-5.07 (-7, -3.14)	-3.95 (-5.23,-2.67)	-1.86 (-3.14,-0.58)	-2.01 (-3.23,-0.79)	-1.84 (-3.08,-0.59)
0.7 (-0.62,2.02)	-1.79 (-2.95,-0.63)	0.51 (-0.51,1.53)	2.75 (1.82,3.69)	-0.29 (-1.58,0.99)	2.77 (1.77,3.78)	2.67 (1.48,3.86)	2.68 (1.76,3.59)	2.86 (1.66,4.06)	-1.6 (-3.52,0.32)	-0.59 (-1.72,0.54)	-1.59 (-2.46,-0.71)	-1.84 (-2.81,-0.86)	-1.13 (-2.36,0.1)	0.64 (-0.35,1.63)	2.2 (1.25,3.16)	1.75 (0.55,2.96)	2.4 (0.98,3.82)	NAT	-1.46 (-2.56,-0.36)	0.6 (-0.2, 1.4)	-2.67 (-4.4,-0.94)	-1.55 (-2.5, -0.61)	0.54 (-0.41, 1.49)	0.39 (-0.47, 1.25)	0.56 (-0.34, 1.46)
2.16 (0.87,3.45)	-0.33 (-1.12,0.46)	1.97 (1.19,2.75)	4.21 (3.34,5.08)	1.17 (-0.08,2.41)	4.24 (3.27,5.2)	4.13 (3,5.27)	4.14 (3.29,4.98)	4.32 (3.16,5.48)	-0.14 (-2.04,1.76)	0.87 (-0.22,1.96)	-0.13 (-0.9,0.64)	-0.38 (-1.31,0.56)	0.33 (-0.86,1.51)	2.1 (1.19,3.02)	3.66 (2.77,4.55)	3.22 (2.05,4.38)	3.86 (2.47,5.26)	1.46 (0.36,2.56)	PIO	2.06 (1.31, 2.81)	-1.21 (-2.92, 0.5)	-0.09 (-0.99, 0.8)	2.0 (1.09, 2.9)	1.85 (1.05, 2.65)	2.02 (1.19, 2.86)
0.1 (-0.95,1.15)	-2.39 (-3.23,-1.55)	-0.09 (-0.72,0.53)	2.15 (1.67,2.63)	-0.89 (-1.9,0.11)	2.17 (1.57,2.78)	2.07 (1.19,2.95)	2.08 (1.63,2.52)	2.26 (1.37,3.15)	-2.2 (-3.95,-0.45)	-1.19 (-1.99,-0.39)	-2.19 (-2.54,-1.84)	-2.44 (-3,-1.88)	-1.73 (-2.66,-0.8)	0.04 (-0.54,0.62)	1.6 (1.08,2.12)	1.15 (0.26,2.05)	1.8 (0.62,2.98)	-0.6 (-1.4,0.2)	-2.06 (-2.81,-1.31)	PLC	-3.27 (-4.81,-1.73)	-2.15 (-2.66, -1.65)	-0.06 (-0.57, 0.45)	-0.21 (-0.53, 0.1)	-0.04 (-0.44, 0.37)
3.37 (1.51,5.23)	0.88 (-0.87,2.63)	3.18 (1.52,4.84)	5.42 (3.81,7.03)	2.38 (0.54,4.21)	5.44 (3.79,7.09)	5.34 (3.57,7.11)	5.35 (3.75,6.94)	5.53 (3.76,7.3)	1.07 (-1.26,3.4)	2.08 (0.35,3.81)	1.08 (-0.49,2.66)	0.83 (-0.8,2.47)	1.54 (-0.26,3.33)	3.31 (1.67,4.95)	4.87 (3.25,6.49)	4.42 (2.65,6.2)	5.07 (3.14,7)	2.67 (0.94,4.4)	1.21 (-0.5,2.92)	3.27 (1.73,4.81)	REP	1.12 (-0.5, 2.73)	3.21 (1.59, 4.83)	3.06 (1.49, 4.63)	3.23 (1.64, 4.82)
2.25 (1.09,3.42)	-0.24 (-1.21,0.74)	2.06 (1.26,2.86)	4.31 (3.64,4.97)	1.26 (0.24,2.29)	4.33 (3.55,5.11)	4.23 (3.22,5.23)	4.23 (3.58,4.88)	4.41 (3.4,5.43)	-0.05 (-1.86,1.77)	0.96 (0.17,1.75)	-0.03 (-0.62,0.55)	-0.28 (-1.02,0.45)	0.42 (-0.6,1.44)	2.2 (1.44,2.95)	3.75 (3.07,4.44)	3.31 (2.28,4.34)	3.95 (2.67,5.23)	1.55 (0.61,2.5)	0.09 (-0.8,0.99)	2.15 (1.65,2.66)	-1.12 (-2.73,0.5)	ROSI	2.09 (1.38, 2.8)	1.94 (1.41, 2.47)	2.12 (1.51, 2.73)
0.16 (-1,1.33)	-2.33 (-3.3,-1.35)	-0.03 (-0.84,0.77)	2.21 (1.52,2.91)	-0.83 (-1.95,0.29)	2.24 (1.51,2.96)	2.13 (1.12,3.15)	2.14 (1.47,2.81)	2.32 (1.3,3.34)	-2.14 (-3.96,-0.32)	-1.13 (-2.07,-0.19)	-2.13 (-2.74,-1.52)	-2.38 (-2.98,-1.78)	-1.67 (-2.72,-0.62)	0.1 (-0.66,0.87)	1.66 (0.95,2.38)	1.22 (0.19,2.24)	1.86 (0.58,3.14)	-0.54 (-1.49,0.41)	-2 (-2.9,-1.09)	0.06 (-0.45,0.57)	-3.21 (-4.83,-1.59)	-2.09 (-2.8,-1.38)	SAXA	-0.15 (-0.73, 0.43)	0.02 (-0.62, 0.67)
0.31 (-0.78,1.41)	-2.18 (-3.06,-1.29)	0.12 (-0.58,0.82)	2.37 (1.88,2.85)	-0.68 (-1.66,0.3)	2.39 (1.73,3.05)	2.28 (1.38,3.19)	2.29 (1.8,2.78)	2.47 (1.53,3.41)	-1.99 (-3.76,-0.21)	-0.98 (-1.81,-0.15)	-1.98 (-2.36,-1.59)	-2.23 (-2.82,-1.63)	-1.52 (-2.4,-0.64)	0.25 (-0.37,0.87)	1.81 (1.32,2.31)	1.37 (0.42,2.32)	2.01 (0.79,3.23)	-0.39 (-1.25,0.47)	-1.85 (-2.65,-1.05)	0.21 (-0.1,0.53)	-3.06 (-4.63,-1.49)	-1.94 (-2.47,-1.41)	0.15 (-0.43,0.73)	SITA	0.17 (-0.31, 0.66)
0.14 (-0.99,1.26)	-2.35 (-3.27,-1.43)	-0.06 (-0.8,0.69)	2.19 (1.59,2.79)	-0.86 (-1.92,0.21)	2.21 (1.49,2.93)	2.11 (1.16,3.06)	2.11 (1.54,2.68)	2.3 (1.32,3.27)	-2.16 (-3.96,-0.37)	-1.15 (-1.94,-0.37)	-2.15 (-2.61,-1.7)	-2.4 (-3.08,-1.72)	-1.69 (-2.7,-0.69)	0.08 (-0.59,0.75)	1.64 (1.01,2.27)	1.19 (0.21,2.18)	1.84 (0.59,3.08)	-0.56 (-1.46,0.34)	-2.02 (-2.86,-1.19)	0.04 (-0.37,0.44)	-3.23 (-4.82,-1.64)	-2.12 (-2.73,-1.51)	-0.02 (-0.67,0.62)	-0.17 (-0.66,0.31)	VILDA
