# Supplementary material for: Comparative Efficacy and Safety of Antidiabetic Drug Regimens Added to Metformin Monotherapy in Patients with Type 2 Diabetes: A Network Meta-Analysis
Source: PLoS One. 2015 Apr 28;10(4):e0125879. doi: 10.1371/journal.pone.0125879 (PMC4412636; doi:10.1371/journal.pone.0125879)
Supplement: S5 Fig — Therapies are reported in alphabetical order. Results are reported in WMD, mmHg (95% CI). Results for changes in systolic blood pressure (SBP) on the top portion of the matrix represent changes in the row-defining treatment vs. those in the column-defining treatment (referent). For changes in SBP, negative values favor the first agent in alphabetical order. Statistically significant results are bolded. Clinically superior results are underlined. Sodium glucose co-transporter-2 (SGLT-2) inhibitors are highlighted. The results on the bottom portion of the matrix represent the reciprocal of the top portion. CANA = canagliflozin; DAPA = dapagliflozin; EMPA = empagliflozin; EMPA/LINA = empagliflozin/linagliptin; EXEN = exenatide; GLIM = glimepiride; GLIP = glipizide; LINA = linagliptin; LIRA = liraglutide; PIO = pioglitazone; PLC = placebo; SAX = saxagliptin; SITA = sitagliptin; VILDA = vildagliptin. (PDF) [file pone.0125879.s008.pdf]

Figure S5. Network Meta-Analysis Results of the Effect of Antidiabetic Therapies on Change in Systolic Blood Pressure From Baseline

**CANA**	0.36 (-3.49, 4.21)	1.29 (-1.86, 4.44)	1.0 (-0.98, 2.98)	-1.3 (-4.33, 1.73)	**-4.4 (-6.06, -2.75)**	**-4.64 (-9.15, -0.13)**	-2.56 (-5.96, 0.84)	-1.1 (-3.15, 0.95)	-1.4 (-5.47, 2.66)	**-4.14 (-5.8, -2.48)**	**-4.78 (-8.01, -1.56)**	**-2.26 (-3.87, -0.65)**	-0.26 (-7.19, 6.67)
-0.36 (-4.21,3.49)	**DAPA**	0.93 (-3.63, 5.5)	0.64 (-3.21, 4.49)	-1.66 (-6.29, 2.96)	**-4.76 (-8.63, -0.9)**	**-5.0 (-7.35, -2.65)**	-2.92 (-7.66, 1.82)	-1.46 (-5.47, 2.55)	-1.76 (-7.12, 3.6)	**-4.5 (-7.97,- 1.03)**	**-5.14 (-9.58, -0.7)**	-2.62 (-6.4, 1.16)	-0.62 (-8.35, 7.11)
-1.29 (-4.44,1.86)	-0.93 (-5.5,3.63)	**EMPA/LINA**	-0.29 (-2.74, 2.16)	-2.59 (-6.49, 1.3)	**-5.69 (-8.65, -2.73)**	**-5.93 (-11.07, -0.8)**	**-3.85 (-6.61, -1.09)**	-2.39 (-5.69, 0.9)	-2.69 (-7.44, 2.05)	**-5.43 (-8.39, -2.47)**	**-6.08 (-10.13, -2.02)**	**-3.55 (-6.65, -0.46)**	-1.55 (-8.97, 5.86)
-1 (-2.98,0.98)	-0.64 (-4.49,3.21)	0.29 (-2.16,2.74)	**EMPA**	-2.3 (-5.34, 0.73)	**-5.4 (-7.06, -3.74)**	**-5.64 (-10.15, -1.13)**	**-3.56 (-6.32, -0.8)**	-2.1 (-4.3, 0.09)	-2.4 (-6.47, 1.66)	**-5.14 (-6.8, -3.48)**	**-5.79 (-9.01, -2.56)**	**-3.26 (-5.15, -1.37)**	-1.26 (-8.26, 5.74)
1.3 (-1.73,4.33)	1.66 (-2.96,6.29)	2.59 (-1.3,6.49)	2.3 (-0.73,5.34)	**EXEN**	**-3.1 (-5.64, -0.56)**	-3.34 (-8.53, 1.85)	-1.26 (-5.36, 2.85)	0.2 (-2.95, 3.35)	-0.1 (-4.6, 4.4)	-2.84 (-5.89, 0.22)	-3.48 (-7.6, 0.64)	-0.96 (-4.03, 2.11)	1.04 (-6.36, 8.45)
4.4 (2.75,6.06)	4.76 (0.9,8.63)	5.69 (2.73,8.65)	5.4 (3.74,7.06)	3.1 (0.56,5.64)	**GLIM**	-0.24 (-4.76, 4.29)	1.84 (-1.38, 5.07)	**3.3 (1.44, 5.16)**	3.0 (-0.71, 6.71)	0.26 (-1.44, 1.96)	-0.38 (-3.63, 2.87)	**2.14 (0.41, 3.87)**	4.14 (-2.82, 11.1)
4.64 (0.13,9.15)	5 (2.65,7.35)	5.93 (0.8,11.07)	5.64 (1.13,10.15)	3.34 (-1.85,8.53)	0.24 (-4.29,4.76)	**GLIP**	2.08 (-3.21, 7.37)	3.54 (-1.11, 8.19)	3.24 (-2.62, 9.09)	0.5 (-3.69, 4.69)	-0.14 (-5.17, 4.88)	2.38 (-2.07, 6.83)	4.38 (-3.7, 12.46)
2.56 (-0.84,5.96)	2.92 (-1.82,7.66)	3.85 (1.09,6.61)	3.56 (0.8,6.32)	1.26 (-2.85,5.36)	-1.84 (-5.07,1.38)	-2.08 (-7.37,3.21)	**LINA**	1.46 (-2.08, 4.99)	1.16 (-3.76, 6.07)	-1.58 (-4.81, 1.64)	-2.23 (-6.48, 2.02)	0.3 (-3.05, 3.65)	2.3 (-5.23, 9.82)
1.1 (-0.95,3.15)	1.46 (-2.55,5.47)	2.39 (-0.9,5.69)	2.1 (-0.09,4.3)	-0.2 (-3.35,2.95)	-3.3 (-5.16,-1.44)	-3.54 (-8.19,1.11)	-1.46 (-4.99,2.08)	**LIRA**	-0.3 (-4.46, 3.85)	**-3.04 (-5.05, -1.03)**	**-3.68 (-7.1,-0.26)**	-1.16 (-2.78, 0.47)	0.84 (-6.09, 7.77)
1.4 (-2.66,5.47)	1.76 (-3.6,7.12)	2.69 (-2.05,7.44)	2.4 (-1.66,6.47)	0.1 (-4.4,4.6)	-3 (-6.71,0.71)	-3.24 (-9.09,2.62)	-1.16 (-6.07,3.76)	0.3 (-3.85,4.46)	**PIO**	-2.74 (-6.82, 1.35)	-3.38 (-8.32, 1.55)	-0.86 (-4.96, 3.24)	1.14 (-6.75, 9.03)
4.14 (2.48,5.8)	4.5 (1.03,7.97)	5.43 (2.47,8.39)	5.14 (3.48,6.8)	2.84 (-0.22,5.89)	-0.26 (-1.96,1.44)	-0.5 (-4.69,3.69)	1.58 (-1.64,4.81)	3.04 (1.03,5.05)	2.74 (-1.35,6.82)	**PLC**	-0.64 (-3.41, 2.13)	**1.88 (0.38, 3.38)**	3.88 (-3.02, 10.79)
4.78 (1.56,8.01)	5.14 (0.7,9.58)	6.08 (2.02,10.13)	5.79 (2.56,9.01)	3.48 (-0.64,7.6)	0.38 (-2.87,3.63)	0.14 (-4.88,5.17)	2.23 (-2.02,6.48)	3.68 (0.26,7.1)	3.38 (-1.55,8.32)	0.64 (-2.13,3.41)	**SAX**	2.52 (-0.63, 5.67)	4.52 (-2.92, 11.96)
2.26 (0.65,3.87)	2.62 (-1.16,6.4)	3.55 (0.46,6.65)	3.26 (1.37,5.15)	0.96 (-2.11,4.03)	-2.14 (-3.87,-0.41)	-2.38 (-6.83,2.07)	-0.3 (-3.65,3.05)	1.16 (-0.47,2.78)	0.86 (-3.24,4.96)	-1.88 (-3.38,-0.38)	-2.52 (-5.67,0.63)	**SITA**	2.0 (-4.74, 8.74)
0.26 (-6.67,7.19)	0.62 (-7.11,8.35)	1.55 (-5.86,8.97)	1.26 (-5.74,8.26)	-1.04 (-8.45,6.36)	-4.14 (-11.1,2.82)	-4.38 (-12.46,3.7)	-2.3 (-9.82,5.23)	-0.84 (-7.77,6.09)	-1.14 (-9.03,6.75)	-3.88 (-10.79,3.02)	-4.52 (-11.96,2.92)	-2 (-8.74,4.74)	**VILDA**
