# Supplementary material for: Comparative Efficacy and Safety of Antidiabetic Drug Regimens Added to Metformin Monotherapy in Patients with Type 2 Diabetes: A Network Meta-Analysis
Source: PLoS One. 2015 Apr 28;10(4):e0125879. doi: 10.1371/journal.pone.0125879 (PMC4412636; doi:10.1371/journal.pone.0125879)
Supplement: S6 Fig — Therapies are reported in alphabetical order. Results for risk of confirmed hypoglycemia on the top portion of the matrix represent relative risks (RRs) of hypoglycemia in the row-defining treatment vs. those the column-defining treatment (referent). For confirmed hypoglycemia, RRs lower than 1 favor the first agent in alphabetical order. Statistically significant results are bolded. Sodium glucose co-transporter-2 (SGLT-2) inhibitors are highlighted. To obtain RRs for comparisons in the opposite direction, reciprocals should be taken or the lower portion of the matrix can be used. ACA = acarbose; ALO = alogliptin; CANA = canagliflozin; DAPA = dapagliflozin; EMPA = empagliflozin; EMPA/LINA = empagliflozin/linagliptin; EXEN = exenatide; GLAR = glargine; GLIC = gliclazide; GLIM = glimepiride; GLIP = glipizide; LINA = linagliptin; LIRA = liraglutide; LIX = lixisenatide; NAT = nateglinide; PIO = pioglitazone; PLC = placebo; REP = repaglinide; ROSI = rosiglitazone; SAX = saxagliptin; SITA = sitagliptin; VILDA = vildagliptin. (PDF) [file pone.0125879.s009.pdf]

Figure S6 . Network Meta-Analysis Results of the Effect of Antidiabetic Therapies on Risk of Confirmed Hypoglycemia

**ACA**	3.1 (0.1, 94.46)	0.56 (0.03, 9.59)	0.52 (0.03, 9.13)	1.0 (0.06, 17.49)	1.34 (0.05, 33.48)	0.28 (0.02, 4.7)	0.07 (0, 1.61)	0.05 (0, 1.11)	0.13 (0.01, 1.94)	**0.04 (0, 0.74)**	1.1 (0.07, 18.46)	0.65 (0.04, 10.9)	0.55 (0.03, 11.14)	0.07 (0, 1.64)	0.4 (0.02, 8.92)	0.51 (0.04, 7.23)	0.03 (0, 1.53)	0.5 (0.03, 9.99)	0.58 (0.03, 10.69)	0.39 (0.02, 6.14)	0.68 (0.04, 11.85)
0.32 (0.01,9.86)	**ALO**	0.18 (0.02, 1.94)	0.17 (0.02, 1.85)	0.32 (0.03, 3.55)	0.43 (0.03, 7.22)	**0.09 (0.01, 0.95)**	**0.02 (0, 0.34)**	**0.02 (0, 0.24)**	**0.04 (0, 0.38)**	**0.01 (0, 0.15)**	0.35 (0.03, 3.72)	0.21 (0.02, 2.19)	0.18 (0.01, 2.32)	**0.02 (0, 0.35)**	0.13 (0.01, 1.89)	0.16 (0.02, 1.4)	**0.01 (0, 0.36)**	0.16 (0.01, 2.08)	0.19 (0.02, 2.19)	0.13 (0.01, 1.22)	0.22 (0.02, 2.4)
1.79 (0.1,30.88)	5.55 (0.52,59.88)	**CANA**	0.94 (0.23, 3.92)	1.79 (0.46, 6.91)	2.4 (0.32, 17.76)	0.5 (0.14, 1.83)	**0.13 (0.02, 0.76)**	**0.09 (0.01, 0.56)**	**0.23 (0.09, 0.59)**	**0.08 (0.02, 0.29)**	1.97 (0.55, 7.08)	1.17 (0.34, 4.02)	0.98 (0.18, 5.31)	**0.13 (0.02, 0.87)**	0.71 (0.11, 4.44)	0.91 (0.33, 2.51)	0.05 (0,1.2)	0.9 (0.17, 4.82)	1.03 (0.23, 4.62)	0.7 (0.24, 2.07)	1.22 (0.31, 4.74)
1.91 (0.11,33.23)	5.91 (0.54,64.58)	1.06 (0.26,4.43)	**DAPA**	1.9 (0.44, 8.29)	2.55 (0.32, 20.47)	0.53 (0.13, 2.16)	**0.14 (0.02, 0.85)**	**0.1 (0.01, 0.63)**	**0.24 (0.07, 0.79)**	**0.08 (0.03, 0.25)**	2.09 (0.52, 8.45)	1.25 (0.33, 4.71)	1.04 (0.18, 6.04)	**0.13 (0.02, 0.97)**	0.76 (0.11, 5.13)	0.97 (0.34, 2.76)	0.05 (0, 1.28)	0.96 (0.17, 5.32)	1.1 (0.24, 5.0)	0.74 (0.23, 2.4)	1.3 (0.3, 5.62)
1 (0.06, 17.65)	3.11 (0.28,34.36)	0.56 (0.14,2.17)	0.53 (0.12,2.3)	**EMPA**	1.34 (0.26, 6.97)	0.28 (0.07, 1.06)	**0.08 (0.01, 0.45)**	**0.05 (0.01, 0.32)**	**0.13 (0.05, 0.35)**	**0.04 (0.01, 0.17)**	1.1 (0.34, 3.57)	0.66 (0.18, 2.39)	0.55 (0.1, 3.06)	**0.07 (0.01, 0.5)**	0.4 (0.06, 2.55)	0.51 (0.17, 1.49)	**0.03 (0, 0.68)**	0.5 (0.09,2.8)	0.58 (0.12, 2.73)	0.39 (0.12, 1.26)	0.68 (0.17, 2.75)
0.75 (0.03,18.76)	2.32 (0.14,38.75)	0.42 (0.06,3.09)	0.39 (0.05,3.15)	0.75 (0.14,3.87)	**EMPA/LINA**	0.21 (0.03, 1.52)	**0.06 (0.01, 0.57)**	**0.04 (0, 0.4)**	**0.09 (0.02, 0.57)**	**0.03 (0, 0.25)**	0.82 (0.14, 4.85)	0.49 (0.07, 3.48)	0.41 (0.04, 3.94)	**0.05 (0, 0.61)**	0.3 (0.03, 3.18)	0.38 (0.06, 2.34)	**0.02 (0, 0.7)**	0.38 (0.04, 3.6)	0.43 (0.05, 3.67)	0.29 (0.04, 1.92)	0.51 (0.07, 3.87)
3.59 (0.21,60.67)	11.12 (1.06,117.21)	2 (0.55,7.34)	1.88 (0.46,7.67)	3.58 (0.94,13.6)	4.8 (0.66,35.1)	**EXEN**	0.27 (0.05, 1.55)	0.18 (0.03, 1.1)	0.46 (0.18, 1.17)	**0.16 (0.04, 0.59)**	**3.94 (1.12, 13.83)**	2.35 (0.68, 8.13)	1.97 (0.56, 6.91)	0.25 (0.04, 1.7)	1.42 (0.23, 8.74)	1.82 (0.7, 4.76)	0.1 (0, 2.35)	1.81 (0.35, 9.43)	2.07 (0.46, 9.3)	1.4 (0.46 ,4.3)	2.44 (0.64, 9.27)
13.37 (0.62,287.65)	41.4 (2.95,580.44)	7.45 (1.32,42)	7.01 (1.18,41.72)	13.31 (2.24,79.08)	17.86 (1.76,181.19)	3.72 (0.65,21.46)	**GLAR**	0.68 (0.08, 5.85)	1.69 (0.36,7.87)	0.58 (0.11, 3.01)	**14.67 (2.58,83.37)**	**8.74 (1.83, 41.81)**	7.32 (0.94, 57.03)	0.94 (0.1, 8.89)	5.3 (0.6, 46.59)	**6.78 (1.46,31.4)**	0.36 (0.01, 10.87)	6.72 (0.89, 50.49)	**7.7 (1.32, 44.79)**	**5.21 (1.36, 19.98)**	**9.09 (1.5, 54.9)**
19.78 (0.9,435.16)	61.23 (4.25,881.28)	11.03 (1.78,68.28)	10.37 (1.58,68.12)	19.69 (3.09,125.54)	26.42 (2.48,281.9)	5.51 (0.91,33.4)	1.48 (0.17,12.8)	**GLIC**	2.51 (0.5, 12.47)	0.86 (0.14,5.33)	**21.7 (3.61, 130.4)**	**12.92 (2.19, 76.35)**	**10.83 (1.33, 87.89)**	1.39 (0.42, 4.59)	**7.84 (1.97, 31.2)**	**10.02 (2.07, 48.56)**	0.53 (0.02,16.41)	**9.94 (1.84, 53.73)**	**11.39 (1.61, 80.78)**	**7.7 (1.42, 41.64)**	**13.44 (2.11, 85.42)**
7.89 (0.52,120.83)	24.43 (2.61,228.68)	4.4 (1.68,11.49)	4.14 (1.27,13.44)	7.86 (2.89,21.39)	10.54 (1.77,62.88)	2.2 (0.86,5.62)	0.59 (0.13,2.74)	0.4 (0.08,1.98)	**GLIM**	**0.34 (0.12, 0.99)**	**8.66 (3.49, 21.49)**	**5.16 (2.1, 12.66)**	**4.32 (1.02, 18.31)**	0.56 (0.1, 3.12)	3.13 (0.64, 15.39)	**4 (2.16, 7.41)**	0.21 (0.01, 4.74)	3.97 (0.92,17.08)	**4.54 (1.28, 16.12)**	**3.07 (1.46, 6.45)**	**5.36 (1.94, 14.82)**
23.03 (1.36,390.38)	71.29 (6.73,754.73)	12.84 (3.4,48.45)	12.07 (4.06,35.89)	22.93 (5.74,91.58)	30.77 (4.06,232.89)	6.41 (1.7,24.14)	1.72 (0.33,8.92)	1.16 (0.19,7.23)	2.92 (1.01,8.42)	**GLIP**	**25.27 (6.8, 93.85)**	**15.05 (4.63, 48.89)**	**12.61 (2.31, 68.78)**	1.62 (0.24, 11.1)	**9.12 (1.43, 58.27)**	**11.67 (4.41, 30.87)**	0.62 (0.03, 15.14)	**11.57 (2.21, 60.49)**	**13.26 (3.45, 51.0)**	**8.97 (3.48,23.1)**	**15.65 (3.9, 62.82)**
0.91 (0.05,15.34)	2.82 (0.27,29.61)	0.51 (0.14,1.83)	0.48 (0.12,1.93)	0.91 (0.28,2.94)	1.22 (0.21,7.19)	0.25 (0.07,0.89)	0.07 (0.01,0.39)	0.05 (0.01,0.28)	0.12 (0.05,0.29)	0.04 (0.01,0.15)	**LINA**	0.6 (0.18, 2.02)	0.5 (0.1,2.61)	**0.06 (0.01, 0.43)**	0.36 (0.06, 2.19)	0.46 (0.18, 1.2)	**0.02 (0,0.6)**	0.46 (0.09, 2.37)	0.52 (0.12, 2.33)	0.35 (0.12, 1.07)	0.62 (0.17, 2.31)
1.53 (0.09,25.53)	4.74 (0.46,49.21)	0.85 (0.25,2.93)	0.8 (0.21,3.03)	1.52 (0.42,5.54)	2.04 (0.29,14.56)	0.43 (0.12,1.48)	0.11 (0.02,0.55)	0.08 (0.01,0.46)	0.19 (0.08,0.48)	0.07 (0.02,0.22)	1.68 (0.49,5.7)	**LIRA**	0.84 (0.16,4.33)	**0.11 (0.02, 0.71)**	0.61 (0.1, 3.63)	0.78 (0.31, 1.96)	**0.04 (0, 0.99)**	0.77 (0.15, 3.86)	0.88 (0.23, 3.4)	0.6 (0.27, 1.33)	1.04 (0.28, 3.84)
1.83 (0.09,37.19)	5.66 (0.43,74.36)	1.02 (0.19,5.51)	0.96 (0.17,5.54)	1.82 (0.33,10.14)	2.44 (0.25,23.47)	0.51 (0.14,1.79)	0.14 (0.02,1.06)	0.09 (0.01,0.75)	0.23 (0.05,0.98)	0.08 (0.01,0.43)	2 (0.38,10.47)	1.19 (0.23,6.17)	**LIX**	0.13 (0.01, 1.14)	0.72 (0.09, 5.97)	0.93 (0.22, 3.83)	0.05 (0, 1.41)	0.92 (0.13, 6.52)	1.05 (0.17, 6.62)	0.71 (0.15, 3.36)	1.24 (0.22, 6.89)
14.19 (0.61,329.56)	43.93 (2.87,673.1)	7.91 (1.15,54.23)	7.44 (1.03,53.49)	14.13 (2,99.62)	18.96 (1.64,218.91)	3.95 (0.59,26.51)	1.06 (0.11,10.01)	0.72 (0.22,2.36)	1.8 (0.32,10.09)	0.62 (0.09,4.21)	15.57 (2.34,103.63)	9.27 (1.41,60.77)	7.77 (0.88,68.66)	**NAT**	**5.62 (1.01, 31.26)**	**7.19 (1.34, 38.65)**	0.38 (0.01, 12.36)	**7.13 (1.09, 46.49)**	**8.17 (1.05, 63.47)**	5.52 (0.92, 33.3)	**9.64 (1.37, 67.67)**
2.52 (0.11,56.81)	7.81 (0.53,115.47)	1.41 (0.23,8.79)	1.32 (0.19,8.98)	2.51 (0.39,16.11)	3.37 (0.31,36.17)	0.7 (0.11,4.31)	0.19 (0.02,1.66)	0.13 (0.03,0.51)	0.32 (0.06,1.57)	0.11 (0.02,0.7)	2.77 (0.46,16.81)	1.65 (0.28,9.88)	1.38 (0.17,11.4)	0.18 (0.03, 0.99)	**PIO**	1.28 (0.25, 6.48)	0.07 (0, 2.14)	1.27 (0.19, 8.27)	1.45 (0.2, 10.56)	0.98 (0.18, 5.42)	1.72 (0.27, 11.01)
1.97 (0.14,28.16)	6.11 (0.71,52.43)	1.1 (0.4,3.04)	1.03 (0.36,2.95)	1.97 (0.67,5.75)	2.64 (0.43,16.28)	0.55 (0.21,1.44)	0.15 (0.03,0.68)	0.1 (0.02,0.48)	0.25 (0.13,0.46)	0.09 (0.03,0.23)	2.17 (0.84,5.61)	1.29 (0.51,3.25)	1.08 (0.26,4.47)	0.14 (0.03, 0.75)	0.78 (0.15,3.96)	**PLC**	0.05 (0, 1.11)	0.99 (0.25, 3.9)	1.14 (0.34, 3.82)	0.77 (0.37, 1.61)	1.34 (0.47, 3.84)
37.34 (0.65,2131.44)	115.58 (2.77,4818.97)	20.81 (0.84,517.7)	19.57 (0.78,491.77)	37.17 (1.47,941.54)	49.88 (1.43,1737.91)	10.39 (0.42,254.14)	2.79 (0.09,84.73)	1.89 (0.06,58.46)	4.73 (0.21,106.14)	1.62 (0.07,39.78)	40.96 (1.68,998.87)	24.39 (1.01,590.23)	20.44 (0.71,590.23)	2.63 (0.08, 85.58)	14.79 (0.47, 467.58)	18.92 (0.9, 398.91)	**REP**	18.76 (0.66,530.73)	21.49 (0.81, 571.92)	14.54 (0.63, 334.73)	**25.37 (1.01,638.33)**
1.99 (0.1,39.57)	6.16 (0.48,78.8)	1.11 (0.21,5.93)	1.04 (0.19,5.79)	1.98 (0.36,10.98)	2.66 (0.28,25.43)	0.55 (0.11,2.89)	0.15 (0.02,1.12)	0.1 (0.02,0.54)	0.25 (0.06,1.09)	0.09 (0.02,0.45)	2.18 (0.42,11.31)	1.3 (0.26,6.52)	1.09 (0.15,7.73)	0.14 (0.02, 0.91)	0.79 (0.12,5.14)	1.01 (0.26, 3.97)	0.05 (0,1.51)	**ROSI**	1.15 (0.19, 6.93)	0.77 (0.17, 3.48)	1.35 (0.25, 7.44)
1.74 (0.09,32.26)	5.38 (0.46,63.47)	0.97 (0.22,4.33)	0.91 (0.2,4.15)	1.73 (0.37,8.15)	2.32 (0.27,19.76)	0.48 (0.11,2.17)	0.13 (0.02,0.76)	0.09 (0.01,0.62)	0.22 (0.06,0.78)	0.08 (0.02,0.29)	1.91 (0.43,8.45)	1.13 (0.29,4.38)	0.95 (0.15,5.99)	0.12 (0.02, 0.95)	0.69 (0.09,5)	0.88 (0.26, 2.96)	0.05 (0,1.24)	0.87 (0.14,5.28)	**SAX**	0.68 (0.22, 2.11)	1.18 (0.25, 5.61)
2.57 (0.16,40.5)	7.95 (0.82,77.14)	1.43 (0.48,4.24)	1.35 (0.42,4.35)	2.56 (0.79,8.23)	3.43 (0.52,22.63)	0.71 (0.23,2.2)	0.19 (0.05,0.74)	0.13 (0.02,0.7)	0.33 (0.16,0.68)	0.11 (0.04,0.29)	2.82 (0.94,8.46)	1.68 (0.75,3.74)	1.41 (0.3,6.63)	0.18 (0.03,1.09)	1.02 (0.18,5.62)	1.3 (0.62, 2.72)	0.07 (0,1.58)	1.29 (0.29,5.8)	1.48 (0.47,4.61)	**SITA**	1.75 (0.53, 5.76)
1.47 (0.08,25.66)	4.56 (0.42,49.89)	0.82 (0.21,3.19)	0.77 (0.18,3.34)	1.47 (0.36,5.9)	1.97 (0.26,14.96)	0.41 (0.11,1.56)	0.11 (0.02,0.66)	0.07 (0.01,0.47)	0.19 (0.07,0.52)	0.06 (0.02,0.26)	1.61 (0.43,6.03)	0.96 (0.26,3.55)	0.81 (0.15,4.47)	0.1 (0.01, 0.73)	0.58 (0.09,3.74)	0.75 (0.26, 2.14)	0.04 (0,0.99)	0.74 (0.13,4.07)	0.85 (0.18,4.03)	0.57 (0.17,1.89)	**VILDA**
