# Supplementary material for: Comparative Efficacy and Safety of Antidiabetic Drug Regimens Added to Metformin Monotherapy in Patients with Type 2 Diabetes: A Network Meta-Analysis
Source: PLoS One. 2015 Apr 28;10(4):e0125879. doi: 10.1371/journal.pone.0125879 (PMC4412636; doi:10.1371/journal.pone.0125879)
Supplement: S7 Fig — Therapies are reported in alphabetical order. Results for risk of urinary tract infection (UTI) on the top portion of the matrix represent relative risks (RRs) of UTI in the row-defining treatment vs. those the column-defining treatment (referent). For UTI, RRs lower than 1 favor the first agent in alphabetical order. Statistically significant results are bolded. Sodium glucose co-transporter-2 (SGLT-2) inhibitors are highlighted. To obtain RRs for comparisons in the opposite direction, reciprocals should be taken or the lower portion of the matrix can be used. ALO/PIO = alogliptin/pioglitazone; ALO = alogliptin; CANA = canagliflozin; DAPA = dapagliflozin; EMPA = empagliflozin; EMPA/LINA = empagliflozin/linagliptin; GLIM = glimepiride; GLIP = glipizide; LINA = linagliptin; PIO = pioglitazone; PLC = placebo; SAX = saxagliptin; SITA = sitagliptin; VILDA = vildagliptin. (PDF) [file pone.0125879.s010.pdf]

Figure S7. Network Meta-Analysis Results of the Effect of Antidiabetic Therapies on Risk of Urinary Tract Infections

**ALO/PIO**	0.83 (0.44,1.58)	0.7 (0.29,1.7)	0.68 (0.28,1.69)	1.45 (0.53,3.98)	1.02 (0.43,2.4)	0.99 (0.42,2.32)	1.6 (0.6,4.29)	0.92 (0.38,2.23)	0.62 (0.37,1.06)	0.87 (0.41,1.85)	0.74 (0.3,1.78)	0.91 (0.38,2.17)	0.97 (0.14,6.63)
1.21 (0.63,2.3)	**ALO**	0.85 (0.36,2)	0.82 (0.34,1.99)	1.75 (0.65,4.69)	1.23 (0.54,2.82)	1.19 (0.52,2.72)	1.93 (0.74,5.06)	1.11 (0.47,2.62)	0.75 (0.39,1.47)	1.06 (0.51,2.17)	0.89 (0.38,2.09)	1.1 (0.48,2.56)	1.18 (0.17,7.91)
1.43 (0.59,3.46)	1.18 (0.5,2.79)	**CANA**	0.97 (0.54,1.76)	**2.07 (1.03,4.15)**	1.46 (0.93,2.27)	1.41 (0.94,2.11)	**2.29 (1.17,4.48)**	1.32 (0.81,2.15)	0.89 (0.36,2.2)	1.25 (0.78,2)	1.05 (0.58,1.89)	1.31 (0.81,2.11)	1.39 (0.22,8.65)
1.47 (0.59,3.63)	1.21 (0.5,2.94)	1.03 (0.57,1.86)	**DAPA**	**2.12 (1.01,4.47)**	1.5 (0.89,2.5)	1.45 (0.89,2.34)	**2.35 (1.07,5.13)**	1.35 (0.78,2.36)	0.92 (0.36,2.31)	1.28 (0.77,2.14)	1.08 (0.55,2.11)	1.34 (0.72,2.5)	1.43 (0.23,8.98)
0.69 (0.25,1.9)	0.57 (0.21,1.54)	0.48 (0.24,0.97)	0.47 (0.22,0.99)	**EMPA/LINA**	0.71 (0.4,1.24)	0.68 (0.38,1.22)	1.11 (0.46,2.67)	0.64 (0.36,1.14)	0.43 (0.15,1.21)	0.6 (0.31,1.19)	0.51 (0.23,1.13)	0.63 (0.3,1.33)	0.67 (0.1,4.47)
0.98 (0.42,2.31)	0.81 (0.35,1.86)	0.69 (0.44,1.07)	0.67 (0.4,1.12)	1.42 (0.8,2.5)	**EMPA**	0.97 (0.77,1.22)	1.57 (0.78,3.15)	0.91 (0.64,1.28)	0.61 (0.26,1.47)	0.86 (0.57,1.3)	0.72 (0.4,1.29)	0.9 (0.54,1.5)	0.96 (0.16,5.86)
1.01 (0.43,2.38)	0.84 (0.37,1.92)	0.71 (0.47,1.06)	0.69 (0.43,1.12)	1.47 (0.82,2.63)	1.03 (0.82,1.3)	**GLIM**	1.62 (0.82,3.23)	0.94 (0.69,1.28)	0.63 (0.26,1.51)	0.89 (0.59,1.33)	0.75 (0.42,1.33)	0.93 (0.56,1.53)	0.99 (0.16,6.05)
0.62 (0.23,1.67)	0.52 (0.2,1.36)	0.44 (0.22,0.86)	0.43 (0.2,0.93)	0.9 (0.37,2.18)	0.64 (0.32,1.28)	0.62 (0.31,1.23)	**GLIP**	0.58 (0.28,1.2)	0.39 (0.14,1.06)	0.55 (0.29,1.04)	**0.46 (0.24,0.89)**	**0.57 (0.36,0.91)**	0.61 (0.09,3.98)
1.08 (0.45,2.61)	0.9 (0.38,2.11)	0.76 (0.46,1.24)	0.74 (0.42,1.28)	1.57 (0.88,2.8)	1.1 (0.78,1.56)	1.07 (0.78,1.46)	1.73 (0.84,3.59)	**LINA**	0.68 (0.28,1.66)	0.95 (0.6,1.5)	0.8 (0.43,1.48)	0.99 (0.57,1.73)	1.05 (0.17,6.54)
1.6 (0.94,2.72)	1.33 (0.68,2.58)	1.12 (0.45,2.76)	1.09 (0.43,2.75)	2.32 (0.83,6.47)	1.63 (0.68,3.92)	1.58 (0.66,3.78)	2.56 (0.94,6.98)	1.48 (0.6,3.63)	**PIO**	1.4 (0.65,3.02)	1.18 (0.48,2.9)	1.46 (0.6,3.54)	1.56 (0.23,10.71)
1.14 (0.54,2.42)	0.95 (0.46,1.94)	0.8 (0.5,1.28)	0.78 (0.47,1.3)	1.65 (0.84,3.26)	1.17 (0.77,1.77)	1.13 (0.75,1.7)	1.83 (0.97,3.47)	1.06 (0.67,1.67)	0.71 (0.33,1.54)	**PLC**	0.84 (0.53,1.34)	1.05 (0.68,1.61)	1.11 (0.19,6.51)
1.36 (0.56,3.28)	1.13 (0.48,2.65)	0.95 (0.53,1.71)	0.93 (0.47,1.81)	1.96 (0.89,4.34)	1.39 (0.77,2.48)	1.34 (0.75,2.38)	2.17 (1.13,4.2)	1.25 (0.68,2.33)	0.85 (0.34,2.09)	1.19 (0.75,1.89)	**SAX**	1.24 (0.78,1.97)	1.32 (0.21,8.22)
1.09 (0.46,2.6)	0.91 (0.39,2.1)	0.77 (0.47,1.24)	0.75 (0.4,1.39)	1.58 (0.75,3.33)	1.12 (0.67,1.86)	1.08 (0.65,1.78)	1.75 (1.09,2.8)	1.01 (0.58,1.76)	0.68 (0.28,1.65)	0.96 (0.62,1.48)	0.81 (0.51,1.27)	**SITA**	1.07 (0.17,6.57)
1.03 (0.15,6.98)	0.85 (0.13,5.72)	0.72 (0.12,4.47)	0.7 (0.11,4.4)	1.48 (0.22,9.84)	1.05 (0.17,6.42)	1.01 (0.17,6.2)	1.64 (0.25,10.74)	0.95 (0.15,5.88)	0.64 (0.09,4.4)	0.9 (0.15,5.24)	0.76 (0.12,4.69)	0.94 (0.15,5.78)	**VILDA**
