# Supplementary material for: Comparative Efficacy and Safety of Antidiabetic Drug Regimens Added to Metformin Monotherapy in Patients with Type 2 Diabetes: A Network Meta-Analysis
Source: PLoS One. 2015 Apr 28;10(4):e0125879. doi: 10.1371/journal.pone.0125879 (PMC4412636; doi:10.1371/journal.pone.0125879)
Supplement: S8 Fig — Therapies are reported in alphabetical order. Results for risk of genital tract infection (GTI) on the top portion of the matrix represent relative risks (RRs) of GTI in the row-defining treatment vs. those the column-defining treatment (referent). For GTI, RRs lower than 1 favor the first agent in alphabetical order. Statistically significant results are bolded. Sodium glucose co-transporter-2 (SGLT-2) inhibitors are highlighted. To obtain RRs for comparisons in the opposite direction, reciprocals should be taken or the lower portion of the matrix can be used. CANA = canagliflozin; DAPA = dapagliflozin; EMPA = empagliflozin; EMPA/LINA = empagliflozin/linagliptin; GLIM = glimepiride; GLIP = glipizide; LINA = linagliptin; PLC = placebo; SITA = sitagliptin. (PDF) [file pone.0125879.s011.pdf]

Figure S8. Network Meta-Analysis Results of the Effect of Antidiabetic Therapies on Risk of Genital Tract Infections

**CANA**	3.72<br>(0.88, 15.62)	2.72<br>(0.9, 8.17)	1.17<br>(0.55, 2.49)	**6.25<br>(3.28, 11.91)**	**16.99<br>(3.52, 82.08)**	**10.49<br>(1.24, 88.76)**	**8.03<br>(2.44, 26.39)**	**3.44<br>(1.61, 7.35)**
0.27<br>(0.06,1.13)	**DAPA**	0.73<br>(0.13, 4.02)	0.32<br>(0.07, 1.42)	1.68<br>(0.38, 7.51)	**4.57<br>(2.39, 8.73)**	2.82<br>(0.23, 34.43)	2.16<br>(0.97, 4.82)	0.93<br>(0.2, 4.37)
0.37<br>(0.12,1.11)	1.37<br>(0.25,7.52)	**EMPA/LINA**	0.43<br>(0.19, 0.96)	2.3<br>(0.9, 5.89)	**6.25<br>(1.01, 38.7)**	3.86<br>(0.48, 30.8)	2.95<br>(0.66, 13.28)	1.27<br>(0.37, 4.36)
0.85<br>(0.4,1.81)	3.17<br>(0.7,14.24)	2.32<br>(1.04,5.17)	**EMPA**	**5.33<br>(3.27, 8.67)**	**14.48<br>(2.82, 74.39)**	**8.94<br>(1.21, 65.97)**	**6.84<br>(1.92,2 4.37)**	**2.93<br>(1.15, 7.5)**
0.16<br>(0.08,0.3)	0.59<br>(0.13,2.65)	0.43<br>(0.17,1.11)	0.19<br>(0.12,0.31)	**GLIM**	2.72<br>(0.53, 13.86)	1.68<br>(0.21, 13.13)	1.28<br>(0.36, 4.53)	0.55<br>(0.22, 1.36)
0.06<br>(0.01,0.28)	0.22<br>(0.11,0.42)	0.16<br>(0.03,0.99)	0.07<br>(0.01,0.35)	0.37<br>(0.07,1.88)	**GLIP**	0.62<br>(0.05, 8.18)	0.47<br>(0.17, 1.33)	0.2<br>(0.04, 1.09)
0.1<br>(0.01,0.81)	0.35<br>(0.03,4.32)	0.26<br>(0.03,2.07)	0.11<br>(0.02,0.83)	0.6<br>(0.08,4.67)	1.62<br>(0.12,21.46)	**LINA**	0.77<br>(0.07, 8.18)	0.33<br>(0.04, 2.99)
0.12<br>(0.04,0.41)	0.46<br>(0.21,1.03)	0.34<br>(0.08,1.52)	0.15<br>(0.04,0.52)	0.78<br>(0.22,2.75)	2.12<br>(0.75,5.94)	1.31<br>(0.12,13.96)	**PLC**	0.43<br>(0.11, 1.62)
0.29<br>(0.14,0.62)	1.08<br>(0.23,5.1)	0.79<br>(0.23,2.72)	0.34<br>(0.13,0.87)	1.82<br>(0.73,4.5)	4.94<br>(0.92,26.53)	3.05<br>(0.33,27.76)	2.33<br>(0.62,8.8)	**SITA**
