# Supplementary material for: Comparative Efficacy and Safety of Antidiabetic Drug Regimens Added to Metformin Monotherapy in Patients with Type 2 Diabetes: A Network Meta-Analysis
Source: PLoS One. 2015 Apr 28;10(4):e0125879. doi: 10.1371/journal.pone.0125879 (PMC4412636; doi:10.1371/journal.pone.0125879)
Supplement: S9 Fig — Therapies are reported in alphabetical order. HbA1c results are reported in WMD, % (99% CI). Results for changes in HbA1c on the top portion of the matrix represent changes in the row-defining treatment vs. those in the column-defining treatment (referent). For changes in HbA1c, negative values favor the first agent in alphabetical order. Statistically significant results of the sensitivity analysis are colored grey. Sodium glucose co-transporter-2 (SGLT-2) inhibitors are highlighted. The results on the bottom portion of the matrix represent the reciprocal of the top portion. ACA = acarbose; ALO = alogliptin; ALO/PIO = alogliptin/pioglitazone; CANA = canagliflozin; COL = colesevelam; DAPA = dapagliflozin; EMPA = empagliflozin; EMPA/LINA = empagliflozin/linagliptin; EXEN = exenatide; GLAR = glargine; GLIB = glibenclamide; GLIC = gliclazide; GLIM = glimepiride; GLIP = glipizide; HbA1c = hemoglobin A1c; LINA = linagliptin; LIRA = liraglutide; LIX = lixisenatide; MIG = miglitol; NAT = nateglinide; PIO = pioglitazone; PLC = placebo; REP = repaglinide; ROSI = rosiglitazone; SAX = saxagliptin; SITA = sitagliptin; VILDA = vildagliptin. (PDF) [file pone.0125879.s012.pdf]

Figure S9. Sensitivity Analysis Results of the Effect of Antidiabetic Therapies on Change in HbA1c From Baseline

**ACA**	-0.21 (-0.69,0.26)	0.45 (-0.04,0.94)	-0.07 (-0.5,0.37)	-0.29 (-0.91,0.33)	-0.31 (-0.75,0.14)	-0.1 (-0.53,0.33)	0.34 (-0.15,0.84)	0.01 (-0.42,0.45)	0.45 (-0.08,0.98)	0.5 (-0.36,1.36)	-0.08 (-0.53,0.36)	-0.06 (-0.48,0.36)	-0.23 (-0.67,0.2)	-0.14 (-0.58,0.29)	0.07 (-0.37,0.52)	-0.22 (-0.7,0.26)	-0.36 (-0.95,0.23)	-0.3 (-0.78,0.17)	-0.1 (-0.54,0.35)	-0.79 (-1.19,-0.38)	0.29 (-0.68,1.26)	-0.03 (-0.48,0.41)	-0.28 (-0.71,0.15)	-0.14 (-0.56,0.27)	-0.16 (-0.58,0.26)
0.21 (-0.26,0.69)	**ALO**	0.66 (0.37,0.96)	0.15 (-0.15,0.44)	-0.08 (-0.6,0.45)	-0.09 (-0.41,0.22)	0.11 (-0.18,0.41)	0.56 (0.18,0.93)	0.23 (-0.07,0.53)	0.66 (0.24,1.08)	0.71 (-0.27,1.69)	0.13 (-0.17,0.43)	0.16 (-0.12,0.43)	-0.02 (-0.33,0.28)	0.07 (-0.23,0.37)	0.29 (-0.02,0.6)	-0.01 (-0.37,0.35)	-0.14 (-0.64,0.36)	-0.09 (-0.44,0.26)	0.12 (-0.15,0.38)	-0.57 (-0.82,-0.32)	0.51 (-0.41,1.42)	0.18 (-0.13,0.49)	-0.07 (-0.36,0.23)	0.07 (-0.19,0.33)	0.05 (-0.23,0.33)
-0.45 (-0.94,0.04)	-0.66 (-0.96,-0.37)	**ALO/PIO**	-0.52 (-0.84,-0.2)	-0.74 (-1.28,-0.2)	-0.76 (-1.09,-0.42)	-0.55 (-0.86,-0.23)	-0.11 (-0.5,0.29)	-0.44 (-0.76,-0.12)	-0.01 (-0.44,0.43)	0.05 (-0.94,1.04)	-0.53 (-0.85,-0.21)	-0.51 (-0.8,-0.22)	-0.69 (-1.01,-0.36)	-0.59 (-0.91,-0.27)	-0.38 (-0.71,-0.05)	-0.67 (-1.05,-0.3)	-0.81 (-1.32,-0.29)	-0.75 (-1.12,-0.39)	-0.55 (-0.82,-0.28)	-1.24 (-1.52,-0.96)	-0.16 (-1.08,0.77)	-0.49 (-0.82,-0.15)	-0.73 (-1.04,-0.42)	-0.6 (-0.88,-0.31)	-0.61 (-0.91,-0.31)
0.07 (-0.37,0.5)	-0.15 (-0.44,0.15)	0.52 (0.2,0.84)	**CANA**	-0.22 (-0.71,0.26)	-0.24 (-0.49,0.01)	-0.03 (-0.25,0.18)	0.41 (0.09,0.73)	0.08 (-0.15,0.31)	0.51 (0.14,0.88)	0.57 (-0.4,1.53)	-0.02 (-0.26,0.23)	0.01 (-0.16,0.18)	-0.17 (-0.4,0.06)	-0.08 (-0.3,0.15)	0.14 (-0.1,0.38)	-0.16 (-0.46,0.15)	-0.29 (-0.75,0.17)	-0.24 (-0.53,0.06)	-0.03 (-0.26,0.2)	-0.72 (-0.88,-0.55)	0.36 (-0.54,1.26)	0.03 (-0.22,0.28)	-0.21 (-0.43,0)	-0.08 (-0.24,0.09)	-0.09 (-0.29,0.1)
0.29 (-0.33,0.91)	0.08 (-0.45,0.6)	0.74 (0.2,1.28)	0.22 (-0.26,0.71)	**COL**	-0.02 (-0.52,0.48)	0.19 (-0.3,0.68)	0.63 (0.09,1.18)	0.3 (-0.19,0.79)	0.74 (0.17,1.3)	0.79 (-0.27,1.85)	0.21 (-0.28,0.69)	0.23 (-0.24,0.71)	0.05 (-0.44,0.55)	0.15 (-0.34,0.64)	0.36 (-0.13,0.86)	0.07 (-0.47,0.6)	-0.07 (-0.7,0.57)	-0.01 (-0.54,0.51)	0.19 (-0.3,0.68)	-0.5 (-0.96,-0.03)	0.58 (-0.41,1.58)	0.25 (-0.21,0.72)	0.01 (-0.47,0.5)	0.15 (-0.32,0.61)	0.13 (-0.35,0.61)
0.31 (-0.14,0.75)	0.09 (-0.22,0.41)	0.76 (0.42,1.09)	0.24 (-0.01,0.49)	0.02 (-0.48,0.52)	**DAPA**	0.21 (-0.04,0.45)	0.65 (0.31,0.99)	0.32 (0.07,0.57)	0.75 (0.36,1.14)	0.81 (-0.16,1.77)	0.22 (-0.05,0.49)	0.25 (0.03,0.47)	0.07 (-0.13,0.28)	0.16 (-0.09,0.42)	0.38 (0.11,0.65)	0.08 (-0.24,0.4)	-0.05 (-0.52,0.42)	0 (-0.31,0.32)	0.21 (-0.05,0.47)	-0.48 (-0.67,-0.29)	0.6 (-0.3,1.5)	0.27 (0,0.54)	0.03 (-0.2,0.26)	0.16 (-0.04,0.37)	0.15 (-0.08,0.37)
0.1 (-0.33,0.53)	-0.11 (-0.41,0.18)	0.55 (0.23,0.86)	0.03 (-0.18,0.25)	-0.19 (-0.68,0.3)	-0.21 (-0.45,0.04)	**EMPA**	0.44 (0.18,0.71)	0.11 (-0.11,0.33)	0.54 (0.17,0.92)	0.6 (-0.36,1.56)	0.02 (-0.23,0.26)	0.04 (-0.12,0.2)	-0.14 (-0.37,0.09)	-0.04 (-0.23,0.14)	0.17 (-0.06,0.41)	-0.12 (-0.42,0.17)	-0.26 (-0.72,0.2)	-0.2 (-0.5,0.09)	0 (-0.23,0.23)	-0.69 (-0.84,-0.53)	0.39 (-0.5,1.29)	0.06 (-0.18,0.31)	-0.18 (-0.39,0.03)	-0.05 (-0.21,0.12)	-0.06 (-0.25,0.13)
-0.34 (-0.84,0.15)	-0.56 (-0.93,-0.18)	0.11 (-0.29,0.5)	-0.41 (-0.73,-0.09)	-0.63 (-1.18,-0.09)	-0.65 (-0.99,-0.31)	-0.44 (-0.71,-0.18)	**EMPA/LINA**	-0.33 (-0.65,-0.01)	0.1 (-0.34,0.54)	0.16 (-0.83,1.14)	-0.43 (-0.76,-0.09)	-0.4 (-0.69,-0.12)	-0.58 (-0.91,-0.25)	-0.49 (-0.76,-0.22)	-0.27 (-0.6,0.06)	-0.57 (-0.95,-0.19)	-0.7 (-1.22,-0.19)	-0.65 (-1.02,-0.27)	-0.44 (-0.77,-0.11)	-1.13 (-1.41,-0.85)	-0.05 (-0.98,0.88)	-0.38 (-0.72,-0.04)	-0.62 (-0.94,-0.31)	-0.49 (-0.78,-0.2)	-0.5 (-0.81,-0.2)
-0.01 (-0.45,0.42)	-0.23 (-0.53,0.07)	0.44 (0.12,0.76)	-0.08 (-0.31,0.15)	-0.3 (-0.79,0.19)	-0.32 (-0.57,-0.07)	-0.11 (-0.33,0.11)	0.33 (0.01,0.65)	**EXEN**	0.43 (0.05,0.81)	0.49 (-0.47,1.45)	-0.1 (-0.35,0.15)	-0.07 (-0.25,0.11)	-0.25 (-0.49,-0.01)	-0.15 (-0.38,0.07)	0.06 (-0.19,0.31)	-0.24 (-0.48,0.01)	-0.37 (-0.83,0.09)	-0.32 (-0.62,-0.02)	-0.11 (-0.35,0.13)	-0.8 (-0.96,-0.63)	0.28 (-0.62,1.18)	-0.05 (-0.3,0.21)	-0.29 (-0.51,-0.07)	-0.16 (-0.34,0.03)	-0.17 (-0.37,0.03)
-0.45 (-0.98,0.08)	-0.66 (-1.08,-0.24)	0.01 (-0.43,0.44)	-0.51 (-0.88,-0.14)	-0.74 (-1.3,-0.17)	-0.75 (-1.14,-0.36)	-0.54 (-0.92,-0.17)	-0.1 (-0.54,0.34)	-0.43 (-0.81,-0.05)	**GLAR**	0.05 (-0.95,1.06)	-0.53 (-0.92,-0.14)	-0.5 (-0.86,-0.15)	-0.68 (-1.06,-0.3)	-0.59 (-0.97,-0.21)	-0.37 (-0.75,0.01)	-0.67 (-1.1,-0.24)	-0.8 (-1.35,-0.25)	-0.75 (-1.17,-0.32)	-0.54 (-0.92,-0.16)	-1.23 (-1.58,-0.89)	-0.15 (-1.1,0.8)	-0.48 (-0.87,-0.09)	-0.72 (-1.09,-0.36)	-0.59 (-0.92,-0.26)	-0.61 (-0.97,-0.24)
-0.5 (-1.36,0.36)	-0.71 (-1.69,0.27)	-0.05 (-1.04,0.94)	-0.57 (-1.53,0.4)	-0.79 (-1.85,0.27)	-0.81 (-1.77,0.16)	-0.6 (-1.56,0.36)	-0.16 (-1.14,0.83)	-0.49 (-1.45,0.47)	-0.05 (-1.06,0.95)	**GLIB**	-0.58 (-1.55,0.38)	-0.56 (-1.51,0.4)	-0.73 (-1.7,0.23)	-0.64 (-1.6,0.32)	-0.43 (-1.39,0.54)	-0.72 (-1.71,0.26)	-0.86 (-1.9,0.19)	-0.8 (-1.78,0.18)	-0.6 (-1.56,0.37)	-1.29 (-2.23,-0.34)	-0.21 (-1.5,1.09)	-0.53 (-1.5,0.43)	-0.78 (-1.74,0.18)	-0.64 (-1.6,0.31)	-0.66 (-1.62,0.3)
0.08 (-0.36,0.53)	-0.13 (-0.43,0.17)	0.53 (0.21,0.85)	0.02 (-0.23,0.26)	-0.21 (-0.69,0.28)	-0.22 (-0.49,0.05)	-0.02 (-0.26,0.23)	0.43 (0.09,0.76)	0.1 (-0.15,0.35)	0.53 (0.14,0.92)	0.58 (-0.38,1.55)	**GLIC**	0.03 (-0.18,0.24)	-0.15 (-0.41,0.1)	-0.06 (-0.31,0.19)	0.16 (-0.1,0.42)	-0.14 (-0.46,0.18)	-0.27 (-0.74,0.2)	-0.22 (-0.48,0.04)	-0.01 (-0.23,0.21)	-0.7 (-0.9,-0.51)	0.38 (-0.53,1.28)	0.05 (-0.16,0.25)	-0.19 (-0.43,0.05)	-0.06 (-0.26,0.14)	-0.08 (-0.28,0.13)
0.06 (-0.36,0.48)	-0.16 (-0.43,0.12)	0.51 (0.22,0.8)	-0.01 (-0.18,0.16)	-0.23 (-0.71,0.24)	-0.25 (-0.47,-0.03)	-0.04 (-0.2,0.12)	0.4 (0.12,0.69)	0.07 (-0.11,0.25)	0.5 (0.15,0.86)	0.56 (-0.4,1.51)	-0.03 (-0.24,0.18)	**GLIM**	-0.18 (-0.38,0.03)	-0.08 (-0.25,0.09)	0.13 (-0.07,0.33)	-0.17 (-0.44,0.11)	-0.3 (-0.75,0.15)	-0.25 (-0.52,0.03)	-0.04 (-0.23,0.15)	-0.73 (-0.85,-0.61)	0.35 (-0.54,1.24)	0.02 (-0.2,0.24)	-0.22 (-0.4,-0.04)	-0.09 (-0.21,0.04)	-0.1 (-0.24,0.04)
0.23 (-0.2,0.67)	0.02 (-0.28,0.33)	0.69 (0.36,1.01)	0.17 (-0.06,0.4)	-0.05 (-0.55,0.44)	-0.07 (-0.28,0.13)	0.14 (-0.09,0.37)	0.58 (0.25,0.91)	0.25 (0.01,0.49)	0.68 (0.3,1.06)	0.73 (-0.23,1.7)	0.15 (-0.1,0.41)	0.18 (-0.03,0.38)	**GLIP**	0.09 (-0.15,0.33)	0.31 (0.06,0.56)	0.01 (-0.3,0.32)	-0.12 (-0.59,0.34)	-0.07 (-0.37,0.24)	0.14 (-0.11,0.38)	-0.55 (-0.73,-0.38)	0.53 (-0.37,1.43)	0.2 (-0.06,0.46)	-0.04 (-0.23,0.15)	0.09 (-0.09,0.27)	0.07 (-0.14,0.29)
0.14 (-0.29,0.58)	-0.07 (-0.37,0.23)	0.59 (0.27,0.91)	0.08 (-0.15,0.3)	-0.15 (-0.64,0.34)	-0.16 (-0.42,0.09)	0.04 (-0.14,0.23)	0.49 (0.22,0.76)	0.15 (-0.07,0.38)	0.59 (0.21,0.97)	0.64 (-0.32,1.6)	0.06 (-0.19,0.31)	0.08 (-0.09,0.25)	-0.09 (-0.33,0.15)	**LINA**	0.22 (-0.03,0.46)	-0.08 (-0.38,0.22)	-0.21 (-0.68,0.25)	-0.16 (-0.46,0.14)	0.05 (-0.19,0.28)	-0.64 (-0.81,-0.48)	0.44 (-0.46,1.33)	0.11 (-0.15,0.36)	-0.14 (-0.36,0.09)	0 (-0.19,0.18)	-0.02 (-0.22,0.18)
-0.07 (-0.52,0.37)	-0.29 (-0.6,0.02)	0.38 (0.05,0.71)	-0.14 (-0.38,0.1)	-0.36 (-0.86,0.13)	-0.38 (-0.65,-0.11)	-0.17 (-0.41,0.06)	0.27 (-0.06,0.6)	-0.06 (-0.31,0.19)	0.37 (-0.01,0.75)	0.43 (-0.54,1.39)	-0.16 (-0.42,0.1)	-0.13 (-0.33,0.07)	-0.31 (-0.56,-0.06)	-0.22 (-0.46,0.03)	**LIRA**	-0.3 (-0.61,0.02)	-0.43 (-0.9,0.04)	-0.38 (-0.69,-0.06)	-0.17 (-0.42,0.08)	-0.86 (-1.05,-0.67)	0.22 (-0.68,1.12)	-0.11 (-0.37,0.15)	-0.35 (-0.59,-0.12)	-0.22 (-0.4,-0.04)	-0.23 (-0.45,-0.01)
0.22 (-0.26,0.7)	0.01 (-0.35,0.37)	0.67 (0.3,1.05)	0.16 (-0.15,0.46)	-0.07 (-0.6,0.47)	-0.08 (-0.4,0.24)	0.12 (-0.17,0.42)	0.57 (0.19,0.95)	0.24 (-0.01,0.48)	0.67 (0.24,1.1)	0.72 (-0.26,1.71)	0.14 (-0.18,0.46)	0.17 (-0.11,0.44)	-0.01 (-0.32,0.3)	0.08 (-0.22,0.38)	0.3 (-0.02,0.61)	**LIX**	-0.13 (-0.64,0.37)	-0.08 (-0.44,0.28)	0.13 (-0.19,0.44)	-0.56 (-0.82,-0.3)	0.52 (-0.4,1.44)	0.19 (-0.13,0.51)	-0.06 (-0.35,0.24)	0.08 (-0.19,0.35)	0.06 (-0.22,0.35)
0.36 (-0.23,0.95)	0.14 (-0.36,0.64)	0.81 (0.29,1.32)	0.29 (-0.17,0.75)	0.07 (-0.57,0.7)	0.05 (-0.42,0.52)	0.26 (-0.2,0.72)	0.7 (0.19,1.22)	0.37 (-0.09,0.83)	0.8 (0.25,1.35)	0.86 (-0.19,1.9)	0.27 (-0.2,0.74)	0.3 (-0.15,0.75)	0.12 (-0.34,0.59)	0.21 (-0.25,0.68)	0.43 (-0.04,0.9)	0.13 (-0.37,0.64)	**MIG**	0.05 (-0.45,0.55)	0.26 (-0.21,0.73)	-0.43 (-0.86,0)	0.65 (-0.33,1.63)	0.32 (-0.15,0.8)	0.08 (-0.38,0.53)	0.21 (-0.23,0.65)	0.2 (-0.25,0.65)
0.3 (-0.17,0.78)	0.09 (-0.26,0.44)	0.75 (0.39,1.12)	0.24 (-0.06,0.53)	0.01 (-0.51,0.54)	0 (-0.32,0.31)	0.2 (-0.09,0.5)	0.65 (0.27,1.02)	0.32 (0.02,0.62)	0.75 (0.32,1.17)	0.8 (-0.18,1.78)	0.22 (-0.04,0.48)	0.25 (-0.03,0.52)	0.07 (-0.24,0.37)	0.16 (-0.14,0.46)	0.38 (0.06,0.69)	0.08 (-0.28,0.44)	-0.05 (-0.55,0.45)	**NAT**	0.21 (-0.09,0.5)	-0.48 (-0.74,-0.23)	0.6 (-0.32,1.51)	0.27 (-0.03,0.57)	0.02 (-0.27,0.32)	0.16 (-0.11,0.42)	0.14 (-0.13,0.42)
0.1 (-0.35,0.54)	-0.12 (-0.38,0.15)	0.55 (0.28,0.82)	0.03 (-0.2,0.26)	-0.19 (-0.68,0.3)	-0.21 (-0.47,0.05)	0 (-0.23,0.23)	0.44 (0.11,0.77)	0.11 (-0.13,0.35)	0.54 (0.16,0.92)	0.6 (-0.37,1.56)	0.01 (-0.21,0.23)	0.04 (-0.15,0.23)	-0.14 (-0.38,0.11)	-0.05 (-0.28,0.19)	0.17 (-0.08,0.42)	-0.13 (-0.44,0.19)	-0.26 (-0.73,0.21)	-0.21 (-0.5,0.09)	**PIO**	-0.69 (-0.87,-0.51)	0.39 (-0.51,1.29)	0.06 (-0.18,0.31)	-0.18 (-0.41,0.05)	-0.05 (-0.23,0.14)	-0.06 (-0.27,0.14)
0.79 (0.38,1.19)	0.57 (0.32,0.82)	1.24 (0.96,1.52)	0.72 (0.55,0.88)	0.5 (0.03,0.96)	0.48 (0.29,0.67)	0.69 (0.53,0.84)	1.13 (0.85,1.41)	0.8 (0.63,0.96)	1.23 (0.89,1.58)	1.29 (0.34,2.23)	0.7 (0.51,0.9)	0.73 (0.61,0.85)	0.55 (0.38,0.73)	0.64 (0.48,0.81)	0.86 (0.67,1.05)	0.56 (0.3,0.82)	0.43 (0,0.86)	0.48 (0.23,0.74)	0.69 (0.51,0.87)	**PLC**	1.08 (0.2,1.96)	0.75 (0.55,0.95)	0.51 (0.36,0.66)	0.64 (0.55,0.74)	0.63 (0.5,0.75)
-0.29 (-1.26,0.68)	-0.51 (-1.42,0.41)	0.16 (-0.77,1.08)	-0.36 (-1.26,0.54)	-0.58 (-1.58,0.41)	-0.6 (-1.5,0.3)	-0.39 (-1.29,0.5)	0.05 (-0.88,0.98)	-0.28 (-1.18,0.62)	0.15 (-0.8,1.1)	0.21 (-1.09,1.5)	-0.38 (-1.28,0.53)	-0.35 (-1.24,0.54)	-0.53 (-1.43,0.37)	-0.44 (-1.33,0.46)	-0.22 (-1.12,0.68)	-0.52 (-1.44,0.4)	-0.65 (-1.63,0.33)	-0.6 (-1.51,0.32)	-0.39 (-1.29,0.51)	-1.08 (-1.96,-0.2)	**REP**	-0.33 (-1.23,0.58)	-0.57 (-1.47,0.32)	-0.44 (-1.33,0.45)	-0.45 (-1.35,0.44)
0.03 (-0.41,0.48)	-0.18 (-0.49,0.13)	0.49 (0.15,0.82)	-0.03 (-0.28,0.22)	-0.25 (-0.72,0.21)	-0.27 (-0.54,0)	-0.06 (-0.31,0.18)	0.38 (0.04,0.72)	0.05 (-0.21,0.3)	0.48 (0.09,0.87)	0.53 (-0.43,1.5)	-0.05 (-0.25,0.16)	-0.02 (-0.24,0.2)	-0.2 (-0.46,0.06)	-0.11 (-0.36,0.15)	0.11 (-0.15,0.37)	-0.19 (-0.51,0.13)	-0.32 (-0.8,0.15)	-0.27 (-0.57,0.03)	-0.06 (-0.31,0.18)	-0.75 (-0.95,-0.55)	0.33 (-0.58,1.23)	**ROSI**	-0.24 (-0.49,0)	-0.11 (-0.31,0.09)	-0.13 (-0.35,0.09)
0.28 (-0.15,0.71)	0.07 (-0.23,0.36)	0.73 (0.42,1.04)	0.21 (0,0.43)	-0.01 (-0.5,0.47)	-0.03 (-0.26,0.2)	0.18 (-0.03,0.39)	0.62 (0.31,0.94)	0.29 (0.07,0.51)	0.72 (0.36,1.09)	0.78 (-0.18,1.74)	0.19 (-0.05,0.43)	0.22 (0.04,0.4)	0.04 (-0.15,0.23)	0.14 (-0.09,0.36)	0.35 (0.12,0.59)	0.06 (-0.24,0.35)	-0.08 (-0.53,0.38)	-0.02 (-0.32,0.27)	0.18 (-0.05,0.41)	-0.51 (-0.66,-0.36)	0.57 (-0.32,1.47)	0.24 (0,0.49)	**SAX**	0.13 (-0.02,0.29)	0.12 (-0.08,0.31)
0.14 (-0.27,0.56)	-0.07 (-0.33,0.19)	0.6 (0.31,0.88)	0.08 (-0.09,0.24)	-0.15 (-0.61,0.32)	-0.16 (-0.37,0.04)	0.05 (-0.12,0.21)	0.49 (0.2,0.78)	0.16 (-0.03,0.34)	0.59 (0.26,0.92)	0.64 (-0.31,1.6)	0.06 (-0.14,0.26)	0.09 (-0.04,0.21)	-0.09 (-0.27,0.09)	0 (-0.18,0.19)	0.22 (0.04,0.4)	-0.08 (-0.35,0.19)	-0.21 (-0.65,0.23)	-0.16 (-0.42,0.11)	0.05 (-0.14,0.23)	-0.64 (-0.74,-0.55)	0.44 (-0.45,1.33)	0.11 (-0.09,0.31)	-0.13 (-0.29,0.02)	**SITA**	-0.02 (-0.16,0.13)
0.16 (-0.26,0.58)	-0.05 (-0.33,0.23)	0.61 (0.31,0.91)	0.09 (-0.1,0.29)	-0.13 (-0.61,0.35)	-0.15 (-0.37,0.08)	0.06 (-0.13,0.25)	0.5 (0.2,0.81)	0.17 (-0.03,0.37)	0.61 (0.24,0.97)	0.66 (-0.3,1.62)	0.08 (-0.13,0.28)	0.1 (-0.04,0.24)	-0.07 (-0.29,0.14)	0.02 (-0.18,0.22)	0.23 (0.01,0.45)	-0.06 (-0.35,0.22)	-0.2 (-0.65,0.25)	-0.14 (-0.42,0.13)	0.06 (-0.14,0.27)	-0.63 (-0.75,-0.5)	0.45 (-0.44,1.35)	0.13 (-0.09,0.35)	-0.12 (-0.31,0.08)	0.02 (-0.13,0.16)	**VILDA**
