# Supplementary material for: Comparative Efficacy and Safety of Antidiabetic Drug Regimens Added to Metformin Monotherapy in Patients with Type 2 Diabetes: A Network Meta-Analysis
Source: PLoS One. 2015 Apr 28;10(4):e0125879. doi: 10.1371/journal.pone.0125879 (PMC4412636; doi:10.1371/journal.pone.0125879)
Supplement: S10 Fig — Therapies are reported in alphabetical order. Results are reported in WMD, kg (95% CI). Results for changes in weight on the top portion of the matrix represent changes in the row-defining treatment vs. those in the column-defining treatment (referent). For changes in weight, negative values favor the first agent in alphabetical order. Statistically significant results of the sensitivity analysis are colored grey. Sodium glucose co-transporter-2 (SGLT-2) inhibitors are highlighted. The results on the bottom portion of the matrix represent the reciprocal of the top portion. ACA = acarbose; ALO = alogliptin; ALO/PIO = alogliptin/pioglitazone; CANA = canagliflozin; COL = colesevelam; DAPA = dapagliflozin; EMPA = empagliflozin; EMPA/LINA = empagliflozin/linagliptin; EXEN = exenatide; GLAR = glargine; GLIB = glibenclamide; GLIC = gliclazide; GLIM = glimepiride; GLIP = glipizide; LINA = linagliptin; LIRA = liraglutide; LIX = lixisenatide; MIG = miglitol; NAT = nateglinide; PIO = pioglitazone; PLC = placebo; REP = repaglinide; ROSI = rosiglitazone; SAX = saxagliptin; SITA = sitagliptin; VILDA = vildagliptin. (PDF) [file pone.0125879.s013.pdf]

Figure S10. Sensitivity Analysis Results of the Effect of Antidiabetic Therapies on Change in Body Weight From Baseline

**ACA**	-2.49 (-4.25,-0.73)	-0.19 (-1.8,1.41)	2.05 (0.54,3.57)	-0.99 (-2.9,0.92)	2.07 (0.49,3.66)	1.97 (0.17,3.77)	1.98 (0.48,3.47)	2.16 (0.36,3.97)	-2.3 (-4.13,-0.47)	-1.29 (-3.03,0.44)	-2.29 (-3.74,-0.83)	-2.54 (-4.1,-0.98)	-1.83 (-3.68,0.01)	-0.06 (-1.63,1.52)	1.5 (-0.04,3.04)	1.05 (-0.76,2.87)	1.7 (-0.37,3.77)	-0.7 (-2.44,1.04)	-2.16 (-3.86,-0.47)	-0.1 (-1.48,1.28)	-3.37 (-5.81,-0.93)	-2.25 (-3.78,-0.73)	-0.16 (-1.69,1.37)	-0.31 (-1.75,1.13)	-0.14 (-1.62,1.34)
2.49 (0.73,4.25)	**ALO/PIO**	2.3 (1.23,3.37)	4.54 (3.29,5.79)	1.5 (-0.21,3.21)	4.56 (3.21,5.92)	4.46 (2.88,6.04)	4.46 (3.24,5.69)	4.65 (3.05,6.25)	0.19 (-2.35,2.73)	1.2 (-0.31,2.71)	0.2 (-0.95,1.35)	-0.05 (-1.37,1.27)	0.66 (-0.98,2.29)	2.43 (1.12,3.75)	3.99 (2.71,5.27)	3.54 (1.93,5.15)	4.19 (2.29,6.09)	1.79 (0.27,3.31)	0.33 (-0.71,1.36)	2.39 (1.29,3.49)	-0.88 (-3.18,1.42)	0.24 (-1.04,1.51)	2.33 (1.05,3.61)	2.18 (1.02,3.34)	2.35 (1.14,3.56)
0.19 (-1.41,1.8)	-2.3 (-3.37,-1.23)	**ALO**	2.25 (1.22,3.27)	-0.8 (-2.35,0.75)	2.27 (1.13,3.41)	2.17 (0.75,3.58)	2.17 (1.17,3.17)	2.35 (0.93,3.78)	-2.11 (-4.54,0.33)	-1.1 (-2.43,0.23)	-2.1 (-3.01,-1.18)	-2.34 (-3.45,-1.24)	-1.64 (-3.11,-0.17)	0.13 (-0.97,1.24)	1.69 (0.64,2.75)	1.25 (-0.19,2.68)	1.89 (0.14,3.64)	-0.51 (-1.85,0.83)	-1.97 (-2.99,-0.95)	0.09 (-0.73,0.92)	-3.18 (-5.36,-1)	-2.06 (-3.11,-1.01)	0.03 (-1.03,1.09)	-0.12 (-1.03,0.8)	0.06 (-0.92,1.03)
-2.05 (-3.57,-0.54)	-4.54 (-5.79,-3.29)	-2.25 (-3.27,-1.22)	**CANA**	-3.05 (-4.46,-1.63)	0.02 (-0.98,1.02)	-0.08 (-1.36,1.2)	-0.08 (-0.88,0.72)	0.11 (-1.22,1.43)	-4.35 (-6.73,-1.97)	-3.34 (-4.54,-2.14)	-4.34 (-5,-3.68)	-4.59 (-5.53,-3.65)	-3.89 (-5.2,-2.57)	-2.11 (-3.04,-1.18)	-0.55 (-1.4,0.3)	-1 (-2.33,0.34)	-0.35 (-2.02,1.32)	-2.75 (-3.98,-1.52)	-4.21 (-5.36,-3.07)	-2.15 (-2.78,-1.52)	-5.42 (-7.54,-3.31)	-4.31 (-5.18,-3.43)	-2.21 (-3.12,-1.31)	-2.37 (-3,-1.73)	-2.19 (-2.98,-1.4)
0.99 (-0.92,2.9)	-1.5 (-3.21,0.21)	0.8 (-0.75,2.35)	3.05 (1.63,4.46)	**COL**	3.07 (1.54,4.6)	2.97 (1.23,4.7)	2.97 (1.55,4.39)	3.15 (1.39,4.92)	-1.31 (-3.96,1.34)	-0.3 (-1.93,1.33)	-1.3 (-2.66,0.07)	-1.54 (-3.03,-0.06)	-0.84 (-2.56,0.88)	0.93 (-0.57,2.44)	2.49 (1.07,3.92)	2.05 (0.28,3.82)	2.69 (0.66,4.73)	0.29 (-1.4,1.98)	-1.17 (-2.81,0.47)	0.89 (-0.43,2.21)	-2.38 (-4.79,0.04)	-1.26 (-2.61,0.09)	0.83 (-0.64,2.3)	0.68 (-0.6,1.96)	0.86 (-0.55,2.26)
-2.07 (-3.66,-0.49)	-4.56 (-5.92,-3.21)	-2.27 (-3.41,-1.13)	-0.02 (-1.02,0.98)	-3.07 (-4.6,-1.54)	**DAPA**	-0.1 (-1.5,1.3)	-0.1 (-1.08,0.88)	0.09 (-1.32,1.49)	-4.37 (-6.8,-1.95)	-3.37 (-4.68,-2.05)	-4.36 (-5.27,-3.46)	-4.61 (-5.43,-3.79)	-3.91 (-5.35,-2.46)	-2.13 (-3.22,-1.04)	-0.57 (-1.6,0.46)	-1.02 (-2.44,0.4)	-0.37 (-2.11,1.36)	-2.77 (-4.09,-1.46)	-4.24 (-5.5,-2.97)	-2.17 (-2.96,-1.38)	-5.44 (-7.61,-3.28)	-4.33 (-5.35,-3.31)	-2.24 (-3.19,-1.28)	-2.39 (-3.25,-1.52)	-2.21 (-3.16,-1.26)
-1.97 (-3.77,-0.17)	-4.46 (-6.04,-2.88)	-2.17 (-3.58,-0.75)	0.08 (-1.2,1.36)	-2.97 (-4.7,-1.23)	0.1 (-1.3,1.5)	**EMPA/LINA**	0 (-1.07,1.08)	0.19 (-1.46,1.83)	-4.27 (-6.84,-1.7)	-3.26 (-4.81,-1.72)	-4.26 (-5.41,-3.11)	-4.51 (-5.87,-3.15)	-3.8 (-5.46,-2.15)	-2.03 (-3.16,-0.9)	-0.47 (-1.77,0.83)	-0.92 (-2.57,0.73)	-0.27 (-2.2,1.66)	-2.67 (-4.24,-1.11)	-4.13 (-5.63,-2.64)	-2.07 (-3.23,-0.91)	-5.34 (-7.67,-3.01)	-4.23 (-5.54,-2.91)	-2.13 (-3.47,-0.8)	-2.28 (-3.48,-1.09)	-2.11 (-3.36,-0.86)
-1.98 (-3.47,-0.48)	-4.46 (-5.69,-3.24)	-2.17 (-3.17,-1.17)	0.08 (-0.72,0.88)	-2.97 (-4.39,-1.55)	0.1 (-0.88,1.08)	0 (-1.08,1.07)	**EMPA**	0.19 (-1.12,1.49)	-4.28 (-6.64,-1.91)	-3.27 (-4.45,-2.09)	-4.26 (-4.86,-3.67)	-4.51 (-5.43,-3.59)	-3.81 (-5.13,-2.49)	-2.03 (-2.83,-1.24)	-0.47 (-1.31,0.36)	-0.92 (-2.24,0.39)	-0.28 (-1.93,1.38)	-2.68 (-3.88,-1.47)	-4.14 (-5.25,-3.02)	-2.08 (-2.66,-1.49)	-5.35 (-7.45,-3.24)	-4.23 (-5.09,-3.37)	-2.14 (-3.02,-1.26)	-2.29 (-2.93,-1.64)	-2.11 (-2.86,-1.36)
-2.16 (-3.97,-0.36)	-4.65 (-6.25,-3.05)	-2.35 (-3.78,-0.93)	-0.11 (-1.43,1.22)	-3.15 (-4.92,-1.39)	-0.09 (-1.49,1.32)	-0.19 (-1.83,1.46)	-0.19 (-1.49,1.12)	**EXEN**	-4.46 (-7.03,-1.89)	-3.45 (-5.02,-1.88)	-4.45 (-5.7,-3.2)	-4.7 (-6.07,-3.32)	-3.99 (-5.68,-2.3)	-2.22 (-3.61,-0.83)	-0.66 (-2.01,0.69)	-1.11 (-2.15,-0.06)	-0.46 (-2.4,1.47)	-2.86 (-4.43,-1.29)	-4.32 (-5.85,-2.79)	-2.26 (-3.43,-1.1)	-5.53 (-7.86,-3.2)	-4.41 (-5.75,-3.07)	-2.32 (-3.66,-0.98)	-2.47 (-3.71,-1.24)	-2.3 (-3.58,-1.02)
2.3 (0.47,4.13)	-0.19 (-2.73,2.35)	2.11 (-0.33,4.54)	4.35 (1.97,6.73)	1.31 (-1.34,3.96)	4.37 (1.95,6.8)	4.27 (1.7,6.84)	4.28 (1.91,6.64)	4.46 (1.89,7.03)	**GLIB**	1.01 (-1.52,3.53)	0.01 (-2.33,2.35)	-0.24 (-2.65,2.17)	0.47 (-2.13,3.07)	2.24 (-0.18,4.66)	3.8 (1.41,6.19)	3.35 (0.78,5.93)	4 (1.23,6.77)	1.6 (-0.92,4.12)	0.14 (-2.36,2.64)	2.2 (-0.09,4.49)	-1.07 (-4.13,1.99)	0.05 (-2.34,2.43)	2.14 (-0.25,4.53)	1.99 (-0.34,4.32)	2.16 (-0.19,4.52)
1.29 (-0.44,3.03)	-1.2 (-2.71,0.31)	1.1 (-0.23,2.43)	3.34 (2.14,4.54)	0.3 (-1.33,1.93)	3.37 (2.05,4.68)	3.26 (1.72,4.81)	3.27 (2.09,4.45)	3.45 (1.88,5.02)	-1.01 (-3.53,1.52)	**GLIC**	-1 (-2.1,0.11)	-1.25 (-2.52,0.03)	-0.54 (-2.13,1.05)	1.23 (-0.04,2.51)	2.79 (1.57,4.01)	2.35 (0.77,3.92)	2.99 (1.12,4.86)	0.59 (-0.9,2.08)	-0.87 (-2.3,0.56)	1.19 (0.14,2.24)	-2.08 (-4.35,0.2)	-0.96 (-2,0.08)	1.13 (-0.11,2.37)	0.98 (-0.11,2.07)	1.15 (0.13,2.18)
2.29 (0.83,3.74)	-0.2 (-1.35,0.95)	2.1 (1.18,3.01)	4.34 (3.68,5)	1.3 (-0.07,2.66)	4.36 (3.46,5.27)	4.26 (3.11,5.41)	4.26 (3.67,4.86)	4.45 (3.2,5.7)	-0.01 (-2.35,2.33)	1 (-0.11,2.1)	**GLIM**	-0.25 (-1.09,0.6)	0.46 (-0.8,1.72)	2.23 (1.5,2.96)	3.79 (3.09,4.49)	3.34 (2.08,4.61)	3.99 (2.38,5.6)	1.59 (0.44,2.74)	0.13 (-0.89,1.14)	2.19 (1.73,2.65)	-1.08 (-3.15,0.99)	0.03 (-0.73,0.8)	2.13 (1.33,2.93)	1.98 (1.47,2.49)	2.15 (1.55,2.75)
2.54 (0.98,4.1)	0.05 (-1.27,1.37)	2.34 (1.24,3.45)	4.59 (3.65,5.53)	1.54 (0.06,3.03)	4.61 (3.79,5.43)	4.51 (3.15,5.87)	4.51 (3.59,5.43)	4.7 (3.32,6.07)	0.24 (-2.17,2.65)	1.25 (-0.03,2.52)	0.25 (-0.6,1.09)	**GLIP**	0.71 (-0.69,2.1)	2.48 (1.43,3.52)	4.04 (3.07,5.01)	3.59 (2.2,4.98)	4.24 (2.53,5.95)	1.84 (0.55,3.12)	0.38 (-0.85,1.6)	2.44 (1.7,3.17)	-0.83 (-2.98,1.32)	0.28 (-0.68,1.25)	2.38 (1.59,3.16)	2.23 (1.44,3.01)	2.4 (1.5,3.3)
1.83 (-0.01,3.68)	-0.66 (-2.29,0.98)	1.64 (0.17,3.11)	3.89 (2.57,5.2)	0.84 (-0.88,2.56)	3.91 (2.46,5.35)	3.8 (2.15,5.46)	3.81 (2.49,5.13)	3.99 (2.3,5.68)	-0.47 (-3.07,2.13)	0.54 (-1.05,2.13)	-0.46 (-1.72,0.8)	-0.71 (-2.1,0.69)	**GLAR**	1.77 (0.36,3.19)	3.33 (2.01,4.65)	2.89 (1.19,4.58)	3.53 (1.56,5.5)	1.13 (-0.48,2.75)	-0.33 (-1.89,1.23)	1.73 (0.51,2.96)	-1.54 (-3.9,0.82)	-0.42 (-1.77,0.92)	1.67 (0.29,3.05)	1.52 (0.37,2.67)	1.69 (0.38,3.01)
0.06 (-1.52,1.63)	-2.43 (-3.75,-1.12)	-0.13 (-1.24,0.97)	2.11 (1.18,3.04)	-0.93 (-2.44,0.57)	2.13 (1.04,3.22)	2.03 (0.9,3.16)	2.03 (1.24,2.83)	2.22 (0.83,3.61)	-2.24 (-4.66,0.18)	-1.23 (-2.51,0.04)	-2.23 (-2.96,-1.5)	-2.48 (-3.52,-1.43)	-1.77 (-3.19,-0.36)	**LINA**	1.56 (0.6,2.52)	1.11 (-0.29,2.51)	1.76 (0.04,3.48)	-0.64 (-1.94,0.66)	-2.1 (-3.31,-0.9)	-0.04 (-0.8,0.72)	-3.31 (-5.47,-1.15)	-2.2 (-3.18,-1.21)	-0.1 (-1.11,0.9)	-0.25 (-1.07,0.56)	-0.08 (-0.96,0.8)
-1.5 (-3.04,0.04)	-3.99 (-5.27,-2.71)	-1.69 (-2.75,-0.64)	0.55 (-0.3,1.4)	-2.49 (-3.92,-1.07)	0.57 (-0.46,1.6)	0.47 (-0.83,1.77)	0.47 (-0.36,1.31)	0.66 (-0.69,2.01)	-3.8 (-6.19,-1.41)	-2.79 (-4.01,-1.57)	-3.79 (-4.49,-3.09)	-4.04 (-5.01,-3.07)	-3.33 (-4.65,-2.01)	-1.56 (-2.52,-0.6)	**LIRA**	-0.45 (-1.81,0.91)	0.2 (-1.49,1.89)	-2.2 (-3.46,-0.95)	-3.66 (-4.83,-2.49)	-1.6 (-2.28,-0.92)	-4.87 (-7,-2.74)	-3.75 (-4.66,-2.85)	-1.66 (-2.6,-0.72)	-1.81 (-2.46,-1.16)	-1.64 (-2.46,-0.81)
-1.05 (-2.87,0.76)	-3.54 (-5.15,-1.93)	-1.25 (-2.68,0.19)	1 (-0.34,2.33)	-2.05 (-3.82,-0.28)	1.02 (-0.4,2.44)	0.92 (-0.73,2.57)	0.92 (-0.39,2.24)	1.11 (0.06,2.15)	-3.35 (-5.93,-0.78)	-2.35 (-3.92,-0.77)	-3.34 (-4.61,-2.08)	-3.59 (-4.98,-2.2)	-2.89 (-4.58,-1.19)	-1.11 (-2.51,0.29)	0.45 (-0.91,1.81)	**LIX**	0.65 (-1.3,2.59)	-1.75 (-3.33,-0.17)	-3.22 (-4.75,-1.68)	-1.15 (-2.33,0.02)	-4.42 (-6.76,-2.09)	-3.31 (-4.66,-1.96)	-1.22 (-2.57,0.14)	-1.37 (-2.61,-0.12)	-1.19 (-2.48,0.1)
-1.7 (-3.77,0.37)	-4.19 (-6.09,-2.29)	-1.89 (-3.64,-0.14)	0.35 (-1.32,2.02)	-2.69 (-4.73,-0.66)	0.37 (-1.36,2.11)	0.27 (-1.66,2.2)	0.28 (-1.38,1.93)	0.46 (-1.47,2.4)	-4 (-6.77,-1.23)	-2.99 (-4.86,-1.12)	-3.99 (-5.6,-2.38)	-4.24 (-5.95,-2.53)	-3.53 (-5.5,-1.56)	-1.76 (-3.48,-0.04)	-0.2 (-1.89,1.49)	-0.65 (-2.59,1.3)	**MIG**	-2.4 (-4.27,-0.53)	-3.86 (-5.7,-2.03)	-1.8 (-3.35,-0.25)	-5.07 (-7.61,-2.53)	-3.95 (-5.63,-2.27)	-1.86 (-3.54,-0.18)	-2.01 (-3.61,-0.41)	-1.84 (-3.47,-0.2)
0.7 (-1.04,2.44)	-1.79 (-3.31,-0.27)	0.51 (-0.83,1.85)	2.75 (1.52,3.98)	-0.29 (-1.98,1.4)	2.77 (1.46,4.09)	2.67 (1.11,4.24)	2.68 (1.47,3.88)	2.86 (1.29,4.43)	-1.6 (-4.12,0.92)	-0.59 (-2.08,0.9)	-1.59 (-2.74,-0.44)	-1.84 (-3.12,-0.55)	-1.13 (-2.75,0.48)	0.64 (-0.66,1.94)	2.2 (0.95,3.46)	1.75 (0.17,3.33)	2.4 (0.53,4.27)	**NAT**	-1.46 (-2.91,-0.02)	0.6 (-0.45,1.65)	-2.67 (-4.95,-0.39)	-1.55 (-2.8,-0.31)	0.54 (-0.71,1.79)	0.39 (-0.75,1.52)	0.56 (-0.62,1.74)
2.16 (0.47,3.86)	-0.33 (-1.36,0.71)	1.97 (0.95,2.99)	4.21 (3.07,5.36)	1.17 (-0.47,2.81)	4.24 (2.97,5.5)	4.13 (2.64,5.63)	4.14 (3.02,5.25)	4.32 (2.79,5.85)	-0.14 (-2.64,2.36)	0.87 (-0.56,2.3)	-0.13 (-1.14,0.89)	-0.38 (-1.6,0.85)	0.33 (-1.23,1.89)	2.1 (0.9,3.31)	3.66 (2.49,4.83)	3.22 (1.68,4.75)	3.86 (2.03,5.7)	1.46 (0.02,2.91)	**PIO**	2.06 (1.07,3.05)	-1.21 (-3.46,1.04)	-0.09 (-1.27,1.09)	2.0 (0.81,3.19)	1.85 (0.8,2.9)	2.02 (0.92,3.12)
0.1 (-1.28,1.48)	-2.39 (-3.49,-1.29)	-0.09 (-0.92,0.73)	2.15 (1.52,2.78)	-0.89 (-2.21,0.43)	2.17 (1.38,2.96)	2.07 (0.91,3.23)	2.08 (1.49,2.66)	2.26 (1.1,3.43)	-2.2 (-4.49,0.09)	-1.19 (-2.24,-0.14)	-2.19 (-2.65,-1.73)	-2.44 (-3.17,-1.7)	-1.73 (-2.96,-0.51)	0.04 (-0.72,0.8)	1.6 (0.92,2.28)	1.15 (-0.02,2.33)	1.8 (0.25,3.35)	-0.6 (-1.65,0.45)	-2.06 (-3.05,-1.07)	**PLC**	-3.27 (-5.29,-1.25)	-2.15 (-2.81,-1.49)	-0.06 (-0.73,0.6)	-0.21 (-0.63,0.2)	-0.04 (-0.57,0.5)
3.37 (0.93,5.81)	0.88 (-1.42,3.18)	3.18 (1,5.36)	5.42 (3.31,7.54)	2.38 (-0.04,4.79)	5.44 (3.28,7.61)	5.34 (3.01,7.67)	5.35 (3.24,7.45)	5.53 (3.2,7.86)	1.07 (-1.99,4.13)	2.08 (-0.2,4.35)	1.08 (-0.99,3.15)	0.83 (-1.32,2.98)	1.54 (-0.82,3.9)	3.31 (1.15,5.47)	4.87 (2.74,7)	4.42 (2.09,6.76)	5.07 (2.53,7.61)	2.67 (0.39,4.95)	1.21 (-1.04,3.46)	3.27 (1.25,5.29)	**REP**	1.12 (-1.01,3.24)	3.21 (1.08,5.33)	3.06 (1,5.12)	3.23 (1.14,5.32)
2.25 (0.73,3.78)	-0.24 (-1.51,1.04)	2.06 (1.01,3.11)	4.31 (3.43,5.18)	1.26 (-0.09,2.61)	4.33 (3.31,5.35)	4.23 (2.91,5.54)	4.23 (3.37,5.09)	4.41 (3.07,5.75)	-0.05 (-2.43,2.34)	0.96 (-0.08,2)	-0.03 (-0.8,0.73)	-0.28 (-1.25,0.68)	0.42 (-0.92,1.77)	2.2 (1.21,3.18)	3.75 (2.85,4.66)	3.31 (1.96,4.66)	3.95 (2.27,5.63)	1.55 (0.31,2.8)	0.09 (-1.09,1.27)	2.15 (1.49,2.81)	-1.12 (-3.24,1.01)	**ROSI**	2.09 (1.16,3.02)	1.94 (1.25,2.63)	2.12 (1.32,2.92)
0.16 (-1.37,1.69)	-2.33 (-3.61,-1.05)	-0.03 (-1.09,1.03)	2.21 (1.31,3.12)	-0.83 (-2.3,0.64)	2.24 (1.28,3.19)	2.13 (0.8,3.47)	2.14 (1.26,3.02)	2.32 (0.98,3.66)	-2.14 (-4.53,0.25)	-1.13 (-2.37,0.11)	-2.13 (-2.93,-1.33)	-2.38 (-3.16,-1.59)	-1.67 (-3.05,-0.29)	0.1 (-0.9,1.11)	1.66 (0.72,2.6)	1.22 (-0.14,2.57)	1.86 (0.18,3.54)	-0.54 (-1.79,0.71)	-2 (-3.19,-0.81)	0.06 (-0.6,0.73)	-3.21 (-5.33,-1.08)	-2.09 (-3.02,-1.16)	**SAX**	-0.15 (-0.91,0.61)	0.02 (-0.83,0.88)
0.31 (-1.13,1.75)	-2.18 (-3.34,-1.02)	0.12 (-0.8,1.03)	2.37 (1.73,3)	-0.68 (-1.96,0.6)	2.39 (1.52,3.25)	2.28 (1.09,3.48)	2.29 (1.64,2.93)	2.47 (1.24,3.71)	-1.99 (-4.32,0.34)	-0.98 (-2.07,0.11)	-1.98 (-2.49,-1.47)	-2.23 (-3.01,-1.44)	-1.52 (-2.67,-0.37)	0.25 (-0.56,1.07)	1.81 (1.16,2.46)	1.37 (0.12,2.61)	2.01 (0.41,3.61)	-0.39 (-1.52,0.75)	-1.85 (-2.9,-0.8)	0.21 (-0.2,0.63)	-3.06 (-5.12,-1)	-1.94 (-2.63,-1.25)	0.15 (-0.61,0.91)	**SITA**	0.17 (-0.46,0.81)
0.14 (-1.34,1.62)	-2.35 (-3.56,-1.14)	-0.06 (-1.03,0.92)	2.19 (1.4,2.98)	-0.86 (-2.26,0.55)	2.21 (1.26,3.16)	2.11 (0.86,3.36)	2.11 (1.36,2.86)	2.3 (1.02,3.58)	-2.16 (-4.52,0.19)	-1.15 (-2.18,-0.13)	-2.15 (-2.75,-1.55)	-2.4 (-3.3,-1.5)	-1.69 (-3.01,-0.38)	0.08 (-0.8,0.96)	1.64 (0.81,2.46)	1.19 (-0.1,2.48)	1.84 (0.2,3.47)	-0.56 (-1.74,0.62)	-2.02 (-3.12,-0.92)	0.04 (-0.5,0.57)	-3.23 (-5.32,-1.14)	-2.12 (-2.92,-1.32)	-0.02 (-0.88,0.83)	-0.17 (-0.81,0.46)	**VILDA**
