# Supplementary material for: Comparative Efficacy and Safety of Antidiabetic Drug Regimens Added to Metformin Monotherapy in Patients with Type 2 Diabetes: A Network Meta-Analysis
Source: PLoS One. 2015 Apr 28;10(4):e0125879. doi: 10.1371/journal.pone.0125879 (PMC4412636; doi:10.1371/journal.pone.0125879)
Supplement: S11 Fig — Therapies are reported in alphabetical order. Results are reported in WMD, mmHg (95% CI). Results for changes in systolic blood pressure (SBP) on the top portion of the matrix represent changes in the row-defining treatment vs. those in the column-defining treatment (referent). For changes in SBP, negative values favor the first agent in alphabetical order. Statistically significant results of the sensitivity analysis are colored grey. Sodium glucose co-transporter-2 (SGLT-2) inhibitors are highlighted. The results on the bottom portion of the matrix represent the reciprocal of the top portion. CANA = canagliflozin; DAPA = dapagliflozin; EMPA = empagliflozin; EMPA/LINA = empagliflozin/linagliptin; EXEN = exenatide; GLIM = glimepiride; GLIP = glipizide; LINA = linagliptin; LIRA = liraglutide; PIO = pioglitazone; PLC = placebo; SAX = saxagliptin; SITA = sitagliptin; VILDA = vildagliptin. (PDF) [file pone.0125879.s014.pdf]

Figure S11. Sensitivity Analysis Results of the Effect of Antidiabetic Therapies on Change in Systolic Blood Pressure From Baseline

**CANA**	0.36 (-4.7,5.42)	1.29 (-2.85,5.43)	1 (-1.6,3.61)	-1.3 (-5.29,2.68)	-4.4 (-6.58,-2.22)	-4.64 (-10.57,1.29)	-2.56 (-7.03,1.91)	-1.1 (-3.79,1.59)	-1.4 (-6.75,3.94)	-4.14 (-6.32,-1.96)	-4.78 (-9.02,-0.54)	-2.26 (-4.37,-0.15)	-0.26 (-9.37,8.84)
-0.36 (-5.42,4.7)	**DAPA**	0.93 (-5.06,6.93)	0.64 (-4.41,5.7)	-1.66 (-7.74,4.42)	-4.76 (-9.84,0.32)	-5.0 (-8.09,-1.91)	-2.92 (-9.14,3.31)	-1.46 (-6.73,3.81)	-1.76 (-8.81,5.28)	-4.5 (-9.06,0.06)	-5.14 (-10.98,0.69)	-2.62 (-7.59,2.35)	-0.62 (-10.78,9.54)
-1.29 (-5.43,2.85)	-0.93 (-6.93,5.06)	**EMPA/LINA**	-0.29 (-3.51,2.93)	-2.59 (-7.72,2.53)	-5.69 (-9.58,-1.8)	-5.93 (-12.68,0.81)	-3.85 (-7.48,-0.22)	-2.39 (-6.72,1.93)	-2.69 (-8.94,3.55)	-5.43 (-9.32,-1.54)	-6.08 (-11.4,-0.75)	-3.55 (-7.62,0.51)	-1.55 (-11.3,8.19)
-1 (-3.61,1.6)	-0.64 (-5.7,4.41)	0.29 (-2.93,3.51)	**EMPA**	-2.3 (-6.29,1.68)	-5.4 (-7.59,-3.22)	-5.64 (-11.57,0.28)	-3.56 (-7.19,0.07)	-2.1 (-4.99,0.78)	-2.4 (-7.75,2.94)	-5.14 (-7.33,-2.96)	-5.79 (-10.03,-1.54)	-3.26 (-5.74,-0.78)	-1.26 (-10.46,7.94)
1.3 (-2.68,5.29)	1.66 (-4.42,7.74)	2.59 (-2.53,7.72)	2.3 (-1.68,6.29)	**EXEN**	-3.1 (-6.43,0.23)	-3.34 (-10.16,3.48)	-1.26 (-6.65,4.14)	0.2 (-3.94,4.34)	-0.1 (-6.01,5.81)	-2.84 (-6.85,1.18)	-3.48 (-8.9,1.94)	-0.96 (-4.99,3.08)	1.04 (-8.69,10.78)
4.4 (2.22,6.58)	4.76 (-0.32,9.84)	5.69 (1.8,9.58)	5.4 (3.22,7.59)	3.1 (-0.23,6.43)	**GLIM**	-0.24 (-6.19,5.71)	1.84 (-2.39,6.08)	3.3 (0.85,5.75)	3 (-1.88,7.88)	0.26 (-1.97,2.5)	-0.38 (-4.65,3.89)	2.14 (-0.13,4.42)	4.14 (-5,13.29)
4.64 (-1.29,10.57)	5 (1.91,8.09)	5.93 (-0.81,12.68)	5.64 (-0.28,11.57)	3.34 (-3.48,10.16)	0.24 (-5.71,6.19)	**GLIP**	2.08 (-4.87,9.04)	3.54 (-2.57,9.65)	3.24 (-4.46,10.93)	0.5 (-5.01,6.01)	-0.14 (-6.75,6.46)	2.38 (-3.47,8.23)	4.38 (-6.24,15)
2.56 (-1.91,7.03)	2.92 (-3.31,9.14)	3.85 (0.22,7.48)	3.56 (-0.07,7.19)	1.26 (-4.14,6.65)	-1.84 (-6.08,2.39)	-2.08 (-9.04,4.87)	**LINA**	1.46 (-3.18,6.1)	1.16 (-5.31,7.62)	-1.58 (-5.82,2.66)	-2.23 (-7.81,3.36)	0.3 (-4.1,4.7)	2.3 (-7.59,12.19)
1.1 (-1.59,3.79)	1.46 (-3.81,6.73)	2.39 (-1.93,6.72)	2.1 (-0.78,4.99)	-0.2 (-4.34,3.94)	-3.3 (-5.75,-0.85)	-3.54 (-9.65,2.57)	-1.46 (-6.1,3.18)	**LIRA**	-0.3 (-5.76,5.16)	-3.04 (-5.68,-0.4)	-3.68 (-8.18,0.81)	-1.16 (-3.29,0.98)	0.84 (-8.27,9.95)
1.4 (-3.94,6.75)	1.76 (-5.28,8.81)	2.69 (-3.55,8.94)	2.4 (-2.94,7.75)	0.1 (-5.81,6.01)	-3 (-7.88,1.88)	-3.24 (-10.93,4.46)	-1.16 (-7.62,5.31)	0.3 (-5.16,5.76)	**PIO**	-2.74 (-8.11,2.63)	-3.38 (-9.87,3.1)	-0.86 (-6.24,4.53)	1.14 (-9.22,11.51)
4.14 (1.96,6.32)	4.5 (-0.06,9.06)	5.43 (1.54,9.32)	5.14 (2.96,7.33)	2.84 (-1.18,6.85)	-0.26 (-2.5,1.97)	-0.5 (-6.01,5.01)	1.58 (-2.66,5.82)	3.04 (0.4,5.68)	2.74 (-2.63,8.11)	**PLC**	-0.64 (-4.28,3)	1.88 (-0.09,3.85)	3.88 (-5.19,12.95)
4.78 (0.54,9.02)	5.14 (-0.69,10.98)	6.08 (0.75,11.4)	5.79 (1.54,10.03)	3.48 (-1.94,8.9)	0.38 (-3.89,4.65)	0.14 (-6.46,6.75)	2.23 (-3.36,7.81)	3.68 (-0.81,8.18)	3.38 (-3.1,9.87)	0.64 (-3,4.28)	**SAX**	2.52 (-1.61,6.66)	4.52 (-5.25,14.3)
2.26 (0.15,4.37)	2.62 (-2.35,7.59)	3.55 (-0.51,7.62)	3.26 (0.78,5.74)	0.96 (-3.08,4.99)	-2.14 (-4.42,0.13)	-2.38 (-8.23,3.47)	-0.3 (-4.7,4.1)	1.16 (-0.98,3.29)	0.86 (-4.53,6.24)	-1.88 (-3.85,0.09)	-2.52 (-6.66,1.61)	**SITA**	2 (-6.86,10.86)
0.26 (-8.84,9.37)	0.62 (-9.54,10.78)	1.55 (-8.19,11.3)	1.26 (-7.94,10.46)	-1.04 (-10.78,8.69)	-4.14 (-13.29,5)	-4.38 (-15,6.24)	-2.3 (-12.19,7.59)	-0.84 (-9.95,8.27)	-1.14 (-11.51,9.22)	-3.88 (-12.95,5.19)	-4.52 (-14.3,5.25)	-2 (-10.86,6.86)	**VILDA**
