# Supplementary material for: Comparative Efficacy and Safety of Antidiabetic Drug Regimens Added to Metformin Monotherapy in Patients with Type 2 Diabetes: A Network Meta-Analysis
Source: PLoS One. 2015 Apr 28;10(4):e0125879. doi: 10.1371/journal.pone.0125879 (PMC4412636; doi:10.1371/journal.pone.0125879)
Supplement: S12 Fig — Therapies are reported in alphabetical order. Results for risk of confirmed hypoglycemia on the top portion of the matrix represent relative risks (RRs) of hypoglycemia in the row-defining treatment vs. those the column-defining treatment (referent). For confirmed hypoglycemia, RRs lower than 1 favor the first agent in alphabetical order. Statistically significant results of the sensitivity analysis are colored grey. Sodium glucose co-transporter-2 (SGLT-2) inhibitors are highlighted. To obtain RRs for comparisons in the opposite direction, reciprocals should be taken or the lower portion of the matrix can be used. ACA = acarbose; ALO = alogliptin; CANA = canagliflozin; DAPA = dapagliflozin; EMPA = empagliflozin; EMPA/LINA = empagliflozin/linagliptin; EXEN = exenatide; GLAR = glargine; GLIC = gliclazide; GLIM = glimepiride; GLIP = glipizide; LINA = linagliptin; LIRA = liraglutide; LIX = lixisenatide; NAT = nateglinide; PIO = pioglitazone; PLC = placebo; REP = repaglinide; ROSI = rosiglitazone; SAX = saxagliptin; SITA = sitagliptin; VILDA = vildagliptin. (PDF) [file pone.0125879.s015.pdf]

Figure S12. Sensitivity Analysis Results of the Effect of Antidiabetic Therapies on Risk of Confirmed Hypoglycemia

ACA	3.1 (0.03,276.52)	0.56 (0.01,23.46)	0.52 (0.01,22.4)	1 (0.02,43.05)	1.34 (0.02,92.13)	0.28 (0.01,11.42)	0.07 (0,4.22)	0.05 (0,2.94)	0.13 (0,4.57)	0.04 (0,1.79)	1.1 (0.03,44.83)	0.65 (0.02,26.39)	0.55 (0.01,28.72)	0.07 (0,4.4)	0.4 (0.01,23.72)	0.51 (0.02,16.66)	0.03 (0,5.45)	0.5 (0.01,25.57)	0.58 (0.01,26.77)	0.39 (0.01,14.61)	0.68 (0.02,29.09)
0.32 (0,28.86)	ALO	0.18 (0.01,4.1)	0.17 (0.01,3.92)	0.32 (0.01,7.56)	0.43 (0.01,17.49)	0.09 (0,1.99)	0.02 (0,0.78)	0.02 (0,0.54)	0.04 (0,0.77)	0.01 (0,0.31)	0.35 (0.02,7.79)	0.21 (0.01,4.57)	0.18 (0.01,5.22)	0.02 (0,0.82)	0.13 (0,4.41)	0.16 (0.01,2.76)	0.01 (0,1.16)	0.16 (0.01,4.63)	0.19 (0.01,4.77)	0.13 (0.01,2.49)	0.22 (0.01,5.1)
1.79 (0.04,75.51)	5.55 (0.24,126.42)	CANA	0.94 (0.14,6.13)	1.79 (0.3,10.57)	2.4 (0.17,33.32)	0.5 (0.09,2.75)	0.13 (0.01,1.3)	0.09 (0.01,1)	0.23 (0.06,0.8)	0.08 (0.01,0.45)	1.97 (0.37,10.59)	1.17 (0.23,5.92)	0.98 (0.11,9.02)	0.13 (0.01,1.59)	0.71 (0.06,7.89)	0.91 (0.24,3.46)	0.05 (0,3.28)	0.9 (0.1,8.17)	1.03 (0.14,7.39)	0.7 (0.17,2.91)	1.22 (0.2,7.27)
1.91 (0.04,81.55)	5.91 (0.25,136.93)	1.06 (0.16,6.94)	DAPA	1.9 (0.27,13.16)	2.55 (0.16,39.39)	0.53 (0.08,3.37)	0.14 (0.01,1.49)	0.1 (0.01,1.15)	0.24 (0.05,1.14)	0.08 (0.02,0.35)	2.09 (0.33,13.1)	1.25 (0.22,7.16)	1.04 (0.1,10.48)	0.13 (0.01,1.8)	0.76 (0.06,9.37)	0.97 (0.24,3.84)	0.05 (0,3.54)	0.96 (0.1,9.12)	1.1 (0.15,8.05)	0.74 (0.16,3.47)	1.3 (0.19,8.91)
1 (0.02,43.43)	3.11 (0.13,73.1)	0.56 (0.09,3.31)	0.53 (0.08,3.65)	EMPA	1.34 (0.15,11.7)	0.28 (0.05,1.62)	0.08 (0.01,0.78)	0.05 (0,0.58)	0.13 (0.03,0.47)	0.04 (0.01,0.27)	1.1 (0.24,5.16)	0.66 (0.12,3.58)	0.55 (0.06,5.26)	0.07 (0.01,0.92)	0.4 (0.03,4.57)	0.51 (0.12,2.08)	0.03 (0,1.88)	0.5 (0.05,4.79)	0.58 (0.08,4.44)	0.39 (0.08,1.82)	0.68 (0.11,4.26)
0.75 (0.01,51.63)	2.32 (0.06,93.91)	0.42 (0.03,5.8)	0.39 (0.03,6.06)	0.75 (0.09,6.5)	EMPA/LINA	0.21 (0.02,2.85)	0.06 (0,1.18)	0.04 (0,0.85)	0.09 (0.01,0.99)	0.03 (0,0.46)	0.82 (0.08,8.48)	0.49 (0.04,6.46)	0.41 (0.02,8.02)	0.05 (0,1.31)	0.3 (0.01,6.7)	0.38 (0.03,4.15)	0.02 (0,2.13)	0.38 (0.02,7.32)	0.43 (0.03,7.19)	0.29 (0.02,3.48)	0.51 (0.04,7.32)
3.59 (0.09,147.48)	11.12 (0.5,245.67)	2 (0.36,11.03)	1.88 (0.3,11.93)	3.58 (0.62,20.69)	4.8 (0.35,65.59)	EXEN	0.27 (0.03,2.69)	0.18 (0.02,1.94)	0.46 (0.13,1.57)	0.16 (0.03,0.89)	3.94 (0.76,20.52)	2.35 (0.46,12.01)	1.97 (0.38,10.25)	0.25 (0.02,3.09)	1.42 (0.13,15.47)	1.82 (0.51,6.44)	0.1 (0,6.43)	1.81 (0.21,15.85)	2.07 (0.29,14.91)	1.4 (0.32,6.12)	2.44 (0.42,14.1)
13.37 (0.24,754.41)	41.4 (1.29,1330.77)	7.45 (0.77,72.31)	7.01 (0.67,73.08)	13.31 (1.28,138.41)	17.86 (0.85,375.22)	3.72 (0.37,37.21)	GLAR	0.68 (0.04,11.52)	1.69 (0.23,12.75)	0.58 (0.07,5.04)	14.67 (1.5,143.91)	8.74 (1.12,68.38)	7.32 (0.49,108.71)	0.94 (0.05,17.99)	5.3 (0.3,92.24)	6.78 (0.9,50.84)	0.36 (0,31.76)	6.72 (0.47,95.16)	7.7 (0.76,77.88)	5.21 (0.89,30.49)	9.09 (0.85,96.6)
19.78 (0.34,1149.37)	61.23 (1.84,2037.13)	11.03 (1,121.08)	10.37 (0.87,123.09)	19.69 (1.73,224.68)	26.42 (1.18,593.13)	5.51 (0.51,58.85)	1.48 (0.09,25.21)	GLIC	2.51 (0.3,20.64)	0.86 (0.08,9.47)	21.7 (2.06,229.08)	12.92 (1.25,133.41)	10.83 (0.69,169.7)	1.39 (0.29,6.67)	7.84 (1.27,48.17)	10.02 (1.26,79.74)	0.53 (0.01,48.25)	9.94 (1.08,91.29)	11.39 (0.87,149.5)	7.7 (0.84,70.76)	13.44 (1.18,152.72)
7.89 (0.22,284.8)	24.43 (1.29,461.77)	4.4 (1.25,15.53)	4.14 (0.88,19.46)	7.86 (2.11,29.3)	10.54 (1.01,110.2)	2.2 (0.64,7.56)	0.59 (0.08,4.44)	0.4 (0.05,3.28)	GLIM	0.34 (0.09,1.38)	8.66 (2.62,28.59)	5.16 (1.58,16.79)	4.32 (0.65,28.83)	0.56 (0.06,5.37)	3.13 (0.39,25.39)	4 (1.78,9.0)	0.21 (0,12.6)	3.97 (0.58,27.01)	4.54 (0.86,23.99)	3.07 (1.16,8.14)	5.36 (1.41,20.39)
23.03 (0.56,950)	71.29 (3.21,1584.11)	12.84 (2.24,73.53)	12.07 (2.88,50.54)	22.93 (3.71,141.52)	30.77 (2.15,439.93)	6.41 (1.12,36.62)	1.72 (0.2,14.95)	1.16 (0.11,12.84)	2.92 (0.73,11.75)	GLIP	25.27 (4.5,141.75)	15.05 (3.2,70.81)	12.61 (1.36,117.23)	1.62 (0.13,20.31)	9.12 (0.8,104.34)	11.67 (3.25,41.92)	0.62 (0.01,41.38)	11.57 (1.32,101.71)	13.26 (2.26,77.88)	8.97 (2.59,31.1)	15.65 (2.52,97.22)
0.91 (0.02,37.25)	2.82 (0.13,61.99)	0.51 (0.09,2.73)	0.48 (0.08,2.99)	0.91 (0.19,4.25)	1.22 (0.12,12.57)	0.25 (0.05,1.32)	0.07 (0.01,0.67)	0.05 (0,0.49)	0.12 (0.03,0.38)	0.04 (0.01,0.22)	LINA	0.6 (0.12,2.97)	0.5 (0.06,4.38)	0.06 (0.01,0.78)	0.36 (0.03,3.86)	0.46 (0.13,1.61)	0.02 (0,1.62)	0.46 (0.05,3.98)	0.52 (0.07,3.72)	0.35 (0.08,1.51)	0.62 (0.11,3.5)
1.53 (0.04,61.83)	4.74 (0.22,102.66)	0.85 (0.17,4.31)	0.8 (0.14,4.61)	1.52 (0.28,8.32)	2.04 (0.15,26.99)	0.43 (0.08,2.18)	0.11 (0.01,0.9)	0.08 (0.01,0.8)	0.19 (0.06,0.63)	0.07 (0.01,0.31)	1.68 (0.34,8.37)	LIRA	0.84 (0.1,7.25)	0.11 (0.01,1.28)	0.61 (0.06,6.38)	0.78 (0.23,2.62)	0.04 (0,2.7)	0.77 (0.09,6.41)	0.88 (0.15,5.2)	0.6 (0.21,1.71)	1.04 (0.19,5.79)
1.83 (0.03,95.86)	5.66 (0.19,167.07)	1.02 (0.11,9.36)	0.96 (0.1,9.61)	1.82 (0.19,17.39)	2.44 (0.12,47.8)	0.51 (0.1,2.65)	0.14 (0.01,2.03)	0.09 (0.01,1.45)	0.23 (0.03,1.55)	0.08 (0.01,0.74)	2 (0.23,17.61)	1.19 (0.14,10.33)	LIX	0.13 (0.01,2.26)	0.72 (0.05,11.6)	0.93 (0.14,5.98)	0.05 (0,4.07)	0.92 (0.07,12.07)	1.05 (0.09,11.81)	0.71 (0.09,5.46)	1.24 (0.13,11.81)
14.19 (0.23,885.44)	43.93 (1.22,1586.87)	7.91 (0.63,99.3)	7.44 (0.56,99.43)	14.13 (1.08,184.03)	18.96 (0.76,472.22)	3.95 (0.32,48.21)	1.06 (0.06,20.25)	0.72 (0.15,3.43)	1.8 (0.19,17.36)	0.62 (0.05,7.71)	15.57 (1.29,188)	9.27 (0.78,109.71)	7.77 (0.44,136.19)	NAT	5.62 (0.59,53.6)	7.19 (0.79,65.56)	0.38 (0,36.91)	7.13 (0.61,83.78)	8.17 (0.55,120.89)	5.52 (0.52,58.55)	9.64 (0.74,124.82)
2.52 (0.04,151.14)	7.81 (0.23,269.14)	1.41 (0.13,15.62)	1.32 (0.11,16.39)	2.51 (0.22,28.89)	3.37 (0.15,76.24)	0.7 (0.06,7.63)	0.19 (0.01,3.29)	0.13 (0.02,0.78)	0.32 (0.04,2.6)	0.11 (0.01,1.25)	2.77 (0.26,29.62)	1.65 (0.16,17.35)	1.38 (0.09,22.13)	0.18 (0.02,1.7)	PIO	1.28 (0.15,10.78)	0.07 (0,6.33)	1.27 (0.11,14.9)	1.45 (0.11,19.69)	0.98 (0.1,9.28)	1.72 (0.15,19.74)
1.97 (0.06,64.9)	6.11 (0.36,103.02)	1.1 (0.29,4.19)	1.03 (0.26,4.11)	1.97 (0.48,8.05)	2.64 (0.24,28.85)	0.55 (0.16,1.94)	0.15 (0.02,1.11)	0.1 (0.01,0.79)	0.25 (0.11,0.56)	0.09 (0.02,0.31)	2.17 (0.62,7.57)	1.29 (0.38,4.35)	1.08 (0.17,6.98)	0.14 (0.02,1.27)	0.78 (0.09,6.59)	PLC	0.05 (0,2.91)	0.99 (0.16,6)	1.14 (0.23,5.6)	0.77 (0.29,2.02)	1.34 (0.34,5.35)
37.34 (0.18,7596.52)	115.58 (0.86,15560.1)	20.81 (0.3,1421.2)	19.57 (0.28,1354.39)	37.17 (0.53,2599.52)	49.88 (0.47,5303.9)	10.39 (0.16,693.95)	2.79 (0.03,247.6)	1.89 (0.02,171.91)	4.73 (0.08,282.09)	1.62 (0.02,108.75)	40.96 (0.62,2725.03)	24.39 (0.37,1606.27)	20.44 (0.25,1698.17)	2.63 (0.03,255.57)	14.79 (0.16,1384.02)	18.92 (0.34,1039.73)	REP	18.76 (0.23,1516.99)	21.49 (0.29,1603.67)	14.54 (0.24,896.87)	25.37 (0.37,1758.66)
1.99 (0.04,101.24)	6.16 (0.22,175.52)	1.11 (0.12,10.05)	1.04 (0.11,9.91)	1.98 (0.21,18.8)	2.66 (0.14,51.71)	0.55 (0.06,4.86)	0.15 (0.01,2.11)	0.1 (0.01,0.92)	0.25 (0.04,1.72)	0.09 (0.01,0.76)	2.18 (0.25,18.95)	1.3 (0.16,10.83)	1.09 (0.08,14.31)	0.14 (0.01,1.65)	0.79 (0.07,9.26)	1.01 (0.17,6.1)	0.05 (0,4.31)	ROSI	1.15 (0.11,12.2)	0.77 (0.11,5.58)	1.35 (0.14,12.72)
1.74 (0.04,80.79)	5.38 (0.21,137.84)	0.97 (0.14,6.93)	0.91 (0.12,6.68)	1.73 (0.23,13.27)	2.32 (0.14,38.73)	0.48 (0.07,3.48)	0.13 (0.01,1.31)	0.09 (0.01,1.15)	0.22 (0.04,1.16)	0.08 (0.01,0.44)	1.91 (0.27,13.5)	1.13 (0.19,6.7)	0.95 (0.08,10.68)	0.12 (0.01,1.81)	0.69 (0.05,9.32)	0.88 (0.18,4.33)	0.05 (0,3.47)	0.87 (0.08,9.3)	SAX	0.68 (0.15,3.01)	1.18 (0.15,9.16)
2.57 (0.07,96.35)	7.95 (0.4,157.55)	1.43 (0.34,5.97)	1.35 (0.29,6.28)	2.56 (0.55,11.88)	3.43 (0.29,40.94)	0.71 (0.16,3.13)	0.19 (0.03,1.12)	0.13 (0.01,1.19)	0.33 (0.12,0.86)	0.11 (0.03,0.39)	2.82 (0.66,11.96)	1.68 (0.59,4.81)	1.41 (0.18,10.8)	0.18 (0.02,1.92)	1.02 (0.11,9.6)	1.3 (0.49,3.43)	0.07 (0,4.24)	1.29 (0.18,9.3)	1.48 (0.33,6.59)	SITA	1.75 (0.36,8.39)
1.47 (0.03,63)	4.56 (0.2,105.83)	0.82 (0.14,4.89)	0.77 (0.11,5.3)	1.47 (0.23,9.15)	1.97 (0.14,28.31)	0.41 (0.07,2.37)	0.11 (0.01,1.17)	0.07 (0.01,0.85)	0.19 (0.05,0.71)	0.06 (0.01,0.4)	1.61 (0.29,9.13)	0.96 (0.17,5.35)	0.81 (0.08,7.66)	0.1 (0.01,1.34)	0.58 (0.05,6.71)	0.75 (0.19,2.97)	0.04 (0,2.73)	0.74 (0.08,6.96)	0.85 (0.11,6.57)	0.57 (0.12,2.75)	VILDA
