# Supplementary material for: Comparative Efficacy and Safety of Antidiabetic Drug Regimens Added to Metformin Monotherapy in Patients with Type 2 Diabetes: A Network Meta-Analysis
Source: PLoS One. 2015 Apr 28;10(4):e0125879. doi: 10.1371/journal.pone.0125879 (PMC4412636; doi:10.1371/journal.pone.0125879)
Supplement: S13 Fig — Therapies are reported in alphabetical order. Results for risk of urinary tract infection (UTI) on the top portion of the matrix represent relative risks (RRs) of UTI in the row-defining treatment vs. those the column-defining treatment (referent). For UTI, RRs lower than 1 favor the first agent in alphabetical order. Statistically significant results of the sensitivity analysis are colored grey. Sodium glucose co-transporter-2 (SGLT-2) inhibitors are highlighted. To obtain RRs for comparisons in the opposite direction, reciprocals should be taken or the lower portion of the matrix can be used. ALO/PIO = alogliptin/pioglitazone; ALO = alogliptin; CANA = canagliflozin; DAPA = dapagliflozin; EMPA = empagliflozin; EMPA/LINA = empagliflozin/linagliptin; GLIM = glimepiride; GLIP = glipizide; LINA = linagliptin; PIO = pioglitazone; PLC = placebo; SAX = saxagliptin; SITA = sitagliptin; VILDA = vildagliptin. (PDF) [file pone.0125879.s016.pdf]

Figure S13. Sensitivity Analysis Results of the Effect of Antidiabetic Therapies on Risk of Urinary Tract Infections

**ALO/PIO**	0.83 (0.36,1.93)	0.7 (0.22,2.24)	0.68 (0.21,2.25)	1.45 (0.38,5.46)	1.02 (0.33,3.14)	0.99 (0.32,3.03)	1.6 (0.44,5.84)	0.92 (0.29,2.93)	0.62 (0.31,1.25)	0.87 (0.33,2.34)	0.74 (0.23,2.35)	0.91 (0.29,2.85)	0.97 (0.08,12.12)
1.21 (0.52,2.81)	**ALO**	0.85 (0.27,2.61)	0.82 (0.26,2.63)	1.75 (0.48,6.4)	1.23 (0.41,3.67)	1.19 (0.4,3.53)	1.93 (0.55,6.85)	1.11 (0.36,3.42)	0.75 (0.31,1.81)	1.06 (0.41,2.72)	0.89 (0.29,2.74)	1.1 (0.37,3.33)	1.18 (0.1,14.41)
1.43 (0.45,4.56)	1.18 (0.38,3.66)	**CANA**	0.97 (0.45,2.12)	2.07 (0.83,5.17)	1.46 (0.81,2.61)	1.41 (0.83,2.39)	2.29 (0.94,5.54)	1.32 (0.69,2.51)	0.89 (0.27,2.92)	1.25 (0.67,2.32)	1.05 (0.48,2.28)	1.31 (0.69,2.46)	1.39 (0.13,15.36)
1.47 (0.44,4.83)	1.21 (0.38,3.88)	1.03 (0.47,2.23)	**DAPA**	2.12 (0.8,5.64)	1.5 (0.76,2.94)	1.45 (0.77,2.73)	2.35 (0.84,6.55)	1.35 (0.65,2.8)	0.92 (0.27,3.09)	1.28 (0.65,2.51)	1.08 (0.45,2.6)	1.34 (0.59,3.04)	1.43 (0.13,16.01)
0.69 (0.18,2.61)	0.57 (0.16,2.1)	0.48 (0.19,1.21)	0.47 (0.18,1.25)	**EMPA/LINA**	0.71 (0.33,1.49)	0.68 (0.32,1.47)	1.11 (0.35,3.52)	0.64 (0.3,1.37)	0.43 (0.11,1.66)	0.6 (0.25,1.48)	0.51 (0.18,1.44)	0.63 (0.24,1.68)	0.67 (0.06,8.09)
0.98 (0.32,3.02)	0.81 (0.27,2.42)	0.69 (0.38,1.23)	0.67 (0.34,1.32)	1.42 (0.67,2.99)	**EMPA**	0.97 (0.71,1.31)	1.57 (0.63,3.92)	0.91 (0.58,1.42)	0.61 (0.19,1.93)	0.86 (0.5,1.48)	0.72 (0.34,1.55)	0.9 (0.46,1.76)	0.96 (0.09,10.37)
1.01 (0.33,3.11)	0.84 (0.28,2.49)	0.71 (0.42,1.2)	0.69 (0.37,1.3)	1.47 (0.68,3.16)	1.03 (0.76,1.4)	**GLIM**	1.62 (0.66,4)	0.94 (0.62,1.41)	0.63 (0.2,1.99)	0.89 (0.52,1.52)	0.75 (0.35,1.59)	0.93 (0.48,1.79)	0.99 (0.09,10.69)
0.62 (0.17,2.28)	0.52 (0.15,1.83)	0.44 (0.18,1.06)	0.43 (0.15,1.19)	0.9 (0.28,2.88)	0.64 (0.26,1.59)	0.62 (0.25,1.52)	**GLIP**	0.58 (0.22,1.5)	0.39 (0.1,1.45)	0.55 (0.24,1.27)	0.46 (0.19,1.09)	0.57 (0.31,1.06)	0.61 (0.05,7.18)
1.08 (0.34,3.44)	0.9 (0.29,2.75)	0.76 (0.4,1.44)	0.74 (0.36,1.53)	1.57 (0.73,3.36)	1.1 (0.7,1.73)	1.07 (0.71,1.6)	1.73 (0.67,4.51)	**LINA**	0.68 (0.21,2.2)	0.95 (0.52,1.73)	0.8 (0.35,1.8)	0.99 (0.48,2.06)	1.05 (0.1,11.6)
1.6 (0.8,3.21)	1.33 (0.55,3.18)	1.12 (0.34,3.67)	1.09 (0.32,3.68)	2.32 (0.6,8.93)	1.63 (0.52,5.16)	1.58 (0.5,4.97)	2.56 (0.69,9.55)	1.48 (0.45,4.81)	**PIO**	1.4 (0.51,3.85)	1.18 (0.36,3.85)	1.46 (0.46,4.68)	1.56 (0.12,19.62)
1.14 (0.43,3.06)	0.95 (0.37,2.44)	0.8 (0.43,1.49)	0.78 (0.4,1.53)	1.65 (0.68,4.04)	1.17 (0.68,2.01)	1.13 (0.66,1.93)	1.83 (0.79,4.24)	1.06 (0.58,1.93)	0.71 (0.26,1.97)	**PLC**	0.84 (0.46,1.55)	1.05 (0.59,1.85)	1.11 (0.11,11.35)
1.36 (0.43,4.33)	1.13 (0.36,3.47)	0.95 (0.44,2.06)	0.93 (0.38,2.23)	1.96 (0.69,5.57)	1.39 (0.64,2.98)	1.34 (0.63,2.86)	2.17 (0.92,5.16)	1.25 (0.56,2.83)	0.85 (0.26,2.77)	1.19 (0.64,2.19)	**SAX**	1.24 (0.68,2.27)	1.32 (0.12,14.59)
1.09 (0.35,3.41)	0.91 (0.3,2.73)	0.77 (0.41,1.44)	0.75 (0.33,1.69)	1.58 (0.59,4.21)	1.12 (0.57,2.19)	1.08 (0.56,2.09)	1.75 (0.94,3.25)	1.01 (0.49,2.1)	0.68 (0.21,2.18)	0.96 (0.54,1.69)	0.81 (0.44,1.47)	**SITA**	1.07 (0.1,11.63)
1.03 (0.08,12.76)	0.85 (0.07,10.41)	0.72 (0.07,7.93)	0.7 (0.06,7.84)	1.48 (0.12,17.83)	1.05 (0.1,11.35)	1.01 (0.09,10.96)	1.64 (0.14,19.38)	0.95 (0.09,10.43)	0.64 (0.05,8.06)	0.9 (0.09,9.13)	0.76 (0.07,8.33)	0.94 (0.09,10.23)	**VILDA**
