# Supplementary material for: Comparative Efficacy and Safety of Antidiabetic Drug Regimens Added to Metformin Monotherapy in Patients with Type 2 Diabetes: A Network Meta-Analysis
Source: PLoS One. 2015 Apr 28;10(4):e0125879. doi: 10.1371/journal.pone.0125879 (PMC4412636; doi:10.1371/journal.pone.0125879)
Supplement: S14 Fig — Therapies are reported in alphabetical order. Results for risk of genital tract infection (GTI) on the top portion of the matrix represent relative risks (RRs) of GTI in the row-defining treatment vs. those the column-defining treatment (referent). For GTI, RRs lower than 1 favor the first agent in alphabetical order. Statistically significant results of the sensitivity analysis are colored grey. Sodium glucose co-transporter-2 (SGLT-2) inhibitors are highlighted. To obtain RRs for comparisons in the opposite direction, reciprocals should be taken or the lower portion of the matrix can be used. CANA = canagliflozin; DAPA = dapagliflozin; EMPA = empagliflozin; EMPA/LINA = empagliflozin/linagliptin; GLIM = glimepiride; GLIP = glipizide; LINA = linagliptin; PLC = placebo; SITA = sitagliptin. (PDF) [file pone.0125879.s017.pdf]

Figure S14. Sensitivity Analysis Results of the Effect of Antidiabetic Therapies on Risk of Genital Tract Infections

**CANA**	3.72 (0.56,24.53)	2.72 (0.64,11.55)	1.17 (0.44,3.15)	6.25 (2.68,14.58)	16.99 (2.14,134.64)	10.49 (0.63,173.66)	8.03 (1.68,38.36)	3.44 (1.27,9.33)
0.27 (0.04,1.78)	**DAPA**	0.73 (0.08,6.88)	0.32 (0.04,2.28)	1.68 (0.24,12.01)	4.57 (1.95,10.7)	2.82 (0.11,75.56)	2.16 (0.75,6.21)	0.93 (0.12,7.11)
0.37 (0.09,1.56)	1.37 (0.15,12.85)	**EMPA/LINA**	0.43 (0.15,1.24)	2.3 (0.67,7.91)	6.25 (0.57,68.64)	3.86 (0.25,59.17)	2.95 (0.41,21.3)	1.27 (0.25,6.42)
0.85 (0.32,2.29)	3.17 (0.44,22.84)	2.32 (0.81,6.66)	**EMPA**	5.33 (2.81,10.1)	14.48 (1.68,124.41)	8.94 (0.65,123.64)	6.84 (1.29,36.33)	2.93 (0.85,10.07)
0.16 (0.07,0.37)	0.59 (0.08,4.24)	0.43 (0.13,1.49)	0.19 (0.1,0.36)	**GLIM**	2.72 (0.32,23.12)	1.68 (0.11,25.06)	1.28 (0.24,6.73)	0.55 (0.17,1.81)
0.06 (0.01,0.47)	0.22 (0.09,0.51)	0.16 (0.01,1.76)	0.07 (0.01,0.59)	0.37 (0.04,3.13)	**GLIP**	0.62 (0.02,18.41)	0.47 (0.12,1.83)	0.2 (0.02,1.84)
0.1 (0.01,1.58)	0.35 (0.01,9.49)	0.26 (0.02,3.98)	0.11 (0.01,1.55)	0.6 (0.04,8.91)	1.62 (0.05,48.33)	**LINA**	0.77 (0.03,17.21)	0.33 (0.02,5.98)
0.12 (0.03,0.6)	0.46 (0.16,1.33)	0.34 (0.05,2.44)	0.15 (0.03,0.78)	0.78 (0.15,4.09)	2.12 (0.55,8.21)	1.31 (0.06,29.39)	**PLC**	0.43 (0.07,2.45)
0.29 (0.11,0.79)	1.08 (0.14,8.3)	0.79 (0.16,4.01)	0.34 (0.1,1.17)	1.82 (0.55,5.98)	4.94 (0.54,44.99)	3.05 (0.17,55.56)	2.33 (0.41,13.35)	**SITA**
