# Supplementary material for: Comparative Efficacy and Safety of Antidiabetic Drug Regimens Added to Metformin Monotherapy in Patients with Type 2 Diabetes: A Network Meta-Analysis
Source: PLoS One. 2015 Apr 28;10(4):e0125879. doi: 10.1371/journal.pone.0125879 (PMC4412636; doi:10.1371/journal.pone.0125879)
Supplement: S1 Table — BID = twice daily; BMI = body mass index; DM = diabetes mellitus; FPG = fasting plasma glucose; HbA1c = hemoglobin A1c; n = number of patients; NR = not reported; OAD = oral antidiabetic drug; SBP = systolic blood pressure; SU = sulfonylurea; SE = standard error; TID = three times a day; qAM = daily AM dosing; qPM = daily PM dosing; QW = weekly. aNumber of study participants evaluated for change in A1c from baseline (sample size may vary for other endpoints). bDoses were given daily, unless otherwise specified. cData given in median (range). (PDF) [file pone.0125879.s018.pdf]

Table S1. Baseline Characteristics of Randomized Controlled Trials Evaluating Antidiabetic Therapies in Addition to Metformin in Adults with Type 2 Diabetes

Author, Year N[a]	Follow Up, weeks	Inclusion criteria	Interventions Evaluated[b]	Population Characteristics (Age, mean, years (SE); Males, %)	Baseline HbA1c, mean, % (SE)	SBP, mean, mmHg (SE)	Baseline weight, mean, kg (SE)	Baseline BMI, mean, kg/m2 (SE)	Duration of DM, mean, years (SE)
**Defronzo 2014 N=674**	24	HbA1c: 7-10.5%<br>Stable therapy for ≥12 weeks<br>Metformin Dose: ≥1500 mg/d (or maximum tolerated dose)<br>BMI: ≤45 kg/m2<br>FPG: < 250 mg/dl	Empagliflozin 25 mg /Linagliptin 5 mg QD	Age: 57.1 (0.88)<br>Males: 54%	7.9 (0.07)	130.9 (1.35)	85.5 (1.76)	NR	58.2% >5 years
			Empagliflozin 10 mg /Linagliptin 5 mg QD	Age: 56.2 (0.89)<br>Males: 62%	8.0 (0.07)	130.5 (1.31)	86.6 (1.64)	NR	49.7% >5 years
			Empagliflozin 25 mg QD	Age: 55.5 (0.85)<br>Males: 46%	8.0 (0.07)	129.2 (1.14)	87.7 (1.49)	NR	57.1% >5 years
			Empagliflozin 10 mg QD	Age: 56.1 (0.90)<br>Males: 57%	8.0 (0.08)	131.6 (1.23)	86.1 (1.55)	NR	53.3% >5 years
			Linagliptin 5 mg QD	Age: 56.2 (0.88)<br>Males: 50%	8.0 (0.08)	128.4 (1.11)	85.0 (1.62)	NR	57.8% >5 years
**Bolli 2014 N=484**	24	HbA1c: 7-10%<br>Stable therapy for ≥12 weeks<br>Metformin Dose: ≥1500 mg/d<br>FPG: < 250 mg/dl	Lixisenatide 20 µg, 1-step titration QD	Age: 55.4 (0.70)<br>Males: 44%	8.0 (0.07)	NR	90.3 (1.50)	33.0 (0.46)	5.8 (0.31)
			Lixisenatide 20 µg, 2-step titration QD	Age: 54.6 (0.70)<br>Males: 45%	8.1 (0.07)	NR	88.0 (1.32)	32.1 (0.38)	6.0 (0.36)
			Placebo	Age: 58.2 (0.77)<br>Males: 45%	8.0 (0.06)	NR	87.9 (1.37)	32.4 (0.43)	6.2 (0.37)
**Derosa 2014 N=167**	26	HbA1c: 7-9%<br>Metformin Dose: Maximum tolerated dose	Glimepiride 2 mg TID	Age: 57.2 (1.00)<br>Males: 50%	7.7 (0.08)	NR	77.0 (0.70)	27.6 (0.14)	0.57 (0.03)
			Vildagliptin 50 mg BID	Age: 59.8 (1.07)<br>Males: 49%	7.9 (0.10)	NR	77.8 (0.74)	27.9 (0.17)	0.58 (0.04)
**Haring 2014 N=637**	24	HbA1c: 7-10%<br>Stable therapy for ≥12 weeks<br>Metformin Dose: ≥1500 mg/d (or maximum tolerated dose)<br>BMI: ≤45 kg/m2	Empagliflozin 10 mg QD	Age: 55.5 (0.67)<br>Males: 58%	7.9 (0.05)	129.6 (0.96)	81.6 (1.26)	29.1 (0.37)	55% >5 years
			Empagliflozin 25 mg QD	Age: 55.6 (0.70)<br>Males: 56%	7.9 (0.06)	130.0 (1.03)	82.2 (1.32)	29.7 (0.39)	59% >5 years
			Placebo	Age:56.0 (0.67)<br>Males: 56%	7.9 (0.06)	128.6 (1.02)	79.7 (1.29)	28.7 (0.36)	50% >5 years
**Nauck 2014 N=1098**	26	HbA1c: 7-9.5%<br>≥6 week lead-in for washout of other OADs and stabilize with metformin ≥1500 mg/d<br>BMI: 25-40 kg/m2<br>Weight: Stable for ≥12 weeks before screening	Sitagliptin 100 mg QD	Age: 54 (0.56)<br>Males: 48%	8.1 (0.06)	127 (0.73)	86 (0.96)	31 (0.23)	7 (0.28)
			Placebo	Age: 55 (0.68)<br>Males: 51%	8.1 (0.08)	128 (0.98)	87 (1.28)	31 (0.30)	7 (0.38)
**Ridderstråle 2014 N=1549**	52	HbA1c: 7-10%<br>BMI: ≤45 kg/m2<br>Stable therapy for ≥12 weeks<br>Metformin Dose: ≥1500 mg/d (or maximum tolerated dose)	Emapgliflozin 25mg QD	Age: 56.2 (0.37)<br>Males: 56%	7.9 (0.03)	133.4 (0.54)	82.5 (0.69)	30.0 (0.19)	45% >5 years
			Glimepiride 1-4mg QD	Age: 55.7 (0.37)<br>Males: 54%	7.9 (0.03)	133.5 (0.57)	83.0 (0.69)	30.3 (0.19)	45% >5 years

**White<br>2014<br>N=160**	12	HbA1c: 7-10%<br>Stable therapy for ≥8 weeks<br>Metformin Dose: ≥1500 mg/d<br>BMI: ≤45 kg/m2	Saxagliptin 2.5 mg BID	Age: 53.9 (1.20)<br>Males: 54%	7.9 (0.11)	128.2 (1.76)	NR	33.7 (0.69)	5.8 (0.74)
			Placebo	Age: 56.6 (1.08)<br>Males: 52%	8.0 (0.09)	127.4 (1.56)	NR	32.5 (0.67)	6.2 (0.45)
**Charbonnel<br>2013<br>N=653**	26	HbA1c: 7-11%<br>Stable therapy for ≥12 weeks<br>Metformin Dose: ≥1500 mg/d<br>FPG: <270 mg/dl	Sitagliptin 100 mg QD	Age: 56.9 (0.55)<br>Males: 55%	8.2 (0.06)	130.0 (0.89)	91.0 (1.14)	32.6 (0.33)	7.6 (0.27)
			Liraglutide 1.2 mg QD	Age: 57.6 (0.60)<br>Males: 55%	8.1 (0.05)	131.9 (0.90)	92.1 (1.13)	32.7 (0.34)	8.2 (0.34)
**Chawla<br>2013<br>N=52**	16	HbA1c: 7.5-11%<br>Stable therapy for ≥4 weeks<br>Metformin Dose: ≥1500 mg/d<br>FPG: ≥140 mg/dl	Sitagliptin 100 mg QD	Age: 49.5 (1.94)<br>Males: 60%	8.1 (0.14)	NR	72.1 (2.76)	29.0 (0.99)	4.1 (0.74)
			Pioglitazone 30 mg QD	Age:52.2 (1.90)<br>Males: 56%	8.2 (0.16)	NR	72.7 (2.15)	28.7 (0.75)	4.5 (0.73)
**Cefalu<br>2013<br>N=1450**	52	HbA1c: 7.0-9.5%<br>Stable therapy for ≥10 weeks<br>Metformin ≥2000 mg/d (or 1500 mg/d if unable to tolerate a higher dose). If lower dose, then up to a 12 week dose-stable run-in period	Glimepride 6 to 8 mg QD	Age: 56.3 (0.41)<br>Males: 55%	7.8 (0.04)	129.5 (0.62)	86.5 (0.90)	30.9 (0.25)	6.6 (0.23)
			Canaglifozin 100 mg QD	Age: 56.4 (0.43)<br>Males: 52%	7.8 (0.04)	130.0 (0.57)	86.9 (0.91)	31.0 (0.24)	6.5 (0.25)
			Canaglifozin 300 mg QD	Age: 55.8 (0.42)<br>Males: 50%	7.8 (0.04)	130.0 (0.63)	86.6 (0.89)	31.2 (0.25)	6.7 (0.25)
**Derosa<br>2013<br>N=167**	52	HbA1c: >8.0%<br>35±8 week run-in of metformin 2500±500 mg/d<br>BMI: 25-30 kg/m2	Vildagliptin 50 mg BID	Age: 54.2 (0.91)<br>Males: 50%	8.1 (0.07)	NR	76.9 (0.63)	27.9 (0.16)	0.5 (0.03)
			Placebo	Age: 52.4 (0.78)<br>Males: 52%	8.2 (0.08)	NR	78.5 (0.70)	27.8 (0.15)	0.5 (0.04)
**Lavalle-González<br>2013<br>N=1284**	26	HbA1c: 7-10.5%<br>FPG: <15mmol/l<br>Stable therapy for ≥8 weeks<br>Metformin Dose: ≥2000 mg/d<br>(≥1500 mg/d if unable to tolerate higher dose)	Canagliflozin 100 mg QD	Age: 55.5 (0.49)<br>Males: 47%	7.9 (0.05)	128.0 (0.66)	88.8 (1.16)	32.4 (0.33)	6.7 (0.28)
			Canagliflozin 300 mg QD	Age: 55.3 (0.48)<br>Males: 45%	7.9 (0.05)	128.7 (0.69)	85.4 (1.09)	31.4 (0.33)	7.1 (0.28)
			Sitagliptin 100 mg QD	Age: 55.5 (0.50)<br>Males: 47%	7.9 (0.05)	128.0 (0.71)	87.7 (1.13)	32.0 (0.32)	6.8 (0.27)
			Placebo	Age: 55.3 (0.72)<br>Males: 51%	8.0 (0.07)	128.0 (0.94)	86.6 (1.66)	31.1 (0.45)	6.8 (0.39)
**Rosenstock<br>2013<br>N=495**	12	HbA1c: 6.5-9%<br>Stable therapy for ≥10 weeks<br>Metformin Dose: ≥1500 mg/d<br>(or maximum tolerated dose)<br>4 week washout of other OADs<br>BMI: ≤40 kg/m2	Empagliflozin 10 mg QD	Age: 59 (1.07)<br>Males: 47%	7.9 (0.08)	132.4 (NR)	87.9 (1.71)	31.4 (0.47)	NR
			Empagliflozin 25 mg QD	Age: 59 (0.97)<br>Males: 53%	8.1 (0.10)	135.3 (NR)	90.5 (2.02)	31.5 (0.57)	NR
			Sitagliptin 100 mg QD	Age: 58 (1.20)<br>Males: 54%	8.1 (0.11)	135.5 (NR)	88.0 (1.78)	31.0 (0.53)	NR
			Placebo	Age: 60 (1.01)<br>Males: 47%	8.0 (0.08)	136.0 (NR)	87.7 (1.86)	31.3 (0.53)	NR
**Rosenstock<br>2013<br>N=634**	24	HbA1c: 7-10%<br>Stable therapy for ≥12 weeks<br>Metformin Dose: ≥1500 mg/d<br>FPG: >250 mg/dl	Lixisenatide 20 µg,<br>2-step titration QD	Age: 57.3 (0.52)<br>Males:48%	8.0 (0.04)	NR	94.0 (1.10)	33.7 (0.35)	6.8 (0.31)
			Exenatide 10 µg BID	Age: 57.6 (0.60)<br>Males: 59%	8.0 (0.05)	NR	96.1 (1.27)	33.5 (0.37)	6.8 (0.28)
**Aschner<br>2012<br>N=515**	24	HbA1c: 7-11%<br>Stable therapy for ≥8 weeks<br>Metformin Dose: ≥1000 mg/d<br>BMI: 25–45 kg/m^2<br>FPG: <15.4 mmol/L	Insulin glargine titrated to FPG 4-5.5 mmol/L QD	Age 53.9 (0.59)<br>Males: 50%	8.5 (0.07)	129.8 (0.88)	83.4 (1.21)	31.1 (0.33)	3.9 (NR)
			Sitagliptin 100 mg QD	Age: 53.3 (0.55)<br>Males: 52%	8.5 (0.07)	131.7 (0.95)	84.2 (1.15)	31.3 (0.31)	4.8 (NR)

**Bergenstal 2012 N=666**	24	HbA1c: 7-10%<br>Stable therapy for ≥12 weeks<br>Metformin Dose: ≥1500 mg/d (or maximum tolerated dose)<br>BMI: ≥25 kg/m2 (or >23 kg/m2 for Asian patients)<br>Weight: Stable (<5% variance) for ≥12 weeks before screening	Sitagliptin 100 mg QD	Age: 55.5 (0.74)<br>Males: 59%	8.0 (0.06)	NR	92.5 (1.48)	32.4 (0.38)	6.0 (0.38)
			Placebo	Age: 56.1 (1.06)<br>Males: 52%	8.0 (0.09)	NR	91.1 (2.00)	32.5 (0.58)	5.5 (0.41)
**DeFronzo 2012 N=1554**	26	HbA1c: 7.5-10%<br>Stable therapy for ≥8 weeks<br>Metformin Dose: ≥1500 mg/d<br>If on 1000mg metformin, then titration to 1500mg for 2 weeks then optional 12 week stabilization<br>BMI: 23-45 kg/m2	Placebo	Age: 55.2 (0.87)<br>Males: 47%	8.5 (0.05)	NR	NR	30.6 (0.42)	6.0 (0.44)
			Alogliptin 12.5 mg QD	Age: 53.1 (0.85)<br>Males: 52%	8.6 (0.06)	NR	NR	31 (0.45)	6.2 (0.49)
			Alogliptin 25 mg QD	Age: 53.7 (0.82)<br>Males: 39%	8.6 (0.06)	NR	NR	31.5 (0.50)	5.6 (0.43)
			Pioglitazone 15 mg QD	Age: 54.1 (0.83)<br>Males: 47%	8.5 (0.06)	NR	NR	31.3 (0.46)	5.7 (0.42)
			Pioglitazone 30 mg QD	Age: 56.1 (0.83)<br>Males: 49%	8.5 (0.06)	NR	NR	31.4 (0.48)	7.6 (0.63)
			Pioglitazone 45 mg QD	Age: 54.5 (0.85)<br>Males: 41%	8.5 (0.06)	NR	NR	30.7 (0.41)	5.7 (0.37)
			Alogliptin 12.5 mg + pioglitazone 15 mg QD	Age: 53.6 (0.87)<br>Males: 46%	8.5 (0.06)	NR	NR	31.5 (0.44)	6.1 (0.48)
			Alogliptin 12.5 mg + pioglitazone 30 mg QD	Age: 55.0 (0.80)<br>Males: 42%	8.5 (0.06)	NR	NR	31.1 (0.45)	5.8 (0.45)
			Alogliptin 12.5 mg + pioglitazone 45 mg QD	Age: 54.0 (0.86)<br>Males: 46%	8.5 (0.06)	NR	NR	31.5 (0.46)	6.6 (0.46)
			Alogliptin 25 mg + pioglitazone 15 mg QD	Age: 54.9 (0.81)<br>Males: 47%	8.5 (0.06)	NR	NR	30.8 (0.41)	6.9 (0.48)
			Alogliptin 25 mg + pioglitazone 30 mg QD	Age: 54.4 (0.85)<br>Males: 42%	8.5 (0.06)	NR	NR	31.9 (0.49)	6.6 (0.53)
			Alogliptin 25 mg + pioglitazone 45 mg QD	Age: 54.2 (0.78)<br>Males: 40%	8.6 (0.06)	NR	NR	30.6 (0.42)	6.2 (0.44)
**Derosa 2012 N=174**	52	HbA1c: 8-11%<br>8+/-2 month run-in period with 2500+/-500 mg/d<br>BMI: 25-30 kg/m2	Exenatide 10 µg BID	Age: 57.3 (0.83)<br>Males: 50%	8.1 (0.09)	NR	89.0 (1.05)	31.9 (0.18)	0.6 (0.02)
			Placebo	Age: 56.7 (0.79)<br>Males: 48%	7.9 (0.07)	NR	90.5 (1.12)	31.7 (0.16)	0.7 (0.03)
**Derosa 2012 N=178**	52	HbA1c: >8%<br>8+/-2 month run-in period with 2500+/-500 mg/d<br>BMI: 25-30 kg/m2	Sitagliptin 100 mg QD	Age: 55.9 (0.92)<br>Males: 46%	8.1 (0.08)	NR	78.4 (0.69)	28.1 (0.13)	0.5 (0.02)
			Placebo	Age: 54.8 (0.85)<br>Males: 51%	8.0 (0.08)	NR	78.6 (0.72)	28.9 (0.21)	0.5 (0.02)
**Gallwitz 2012 N=1029**	52	HbA1c: 6.5-9%<br>Stable therapy for ≥12 weeks<br>Metformin Dose: Max tolerated dose<br>BMI: 25-40 kg/m2	Exenatide 10 µg BID	Age: 56 (0.45)<br>Males: 56%	7.5 (0.03)	132.8 (0.71)	92.8 (0.75)	32.6 (0.19)	5.8 (0.22)
			Glimepiride, up to maximum tolerable dose	Age: 56 (0.41)<br>Males: 52%	7.4 (0.03)	133.4 (0.68)	91.1 (0.67)	32.3 (0.18)	5.5 (0.19)

**Gallwitz 2012 N=1551**	52	HbA1C: 6.5-10%<br>Metformin Dose: ≥1500 mg/d (or maximum tolerated dose)<br>BMI: ≤40 kg/m2<br>If on dual therapy, entered 6week washout period	Linagliptin 5 mg QD	Age: 59.8 (0.34)<br>Males: 60%	7.7 (0.03)	NR	86.1 (0.63)	30.2 (0.17)	52 % >5 years
			Glimepiride 1-4 mg QD	Age: 59.8 (0.34)<br>Males: 61%	7.7 (0.03)	NR	86.8 (0.60)	30.3 (0.17)	54% >5 years
**Ljunggren 2012 N=182**	50	HbA1C: 6.5-8.5%<br>Stable therapy for ≥12 weeks<br>Metformin Dose: ≥1500 mg/d<br>BMI: ≥25 kg/m2<br>Weight: ≤120 kg<br>FPG: ≤237.8 mg/dl	Dapagliflozin 10 mg QD	Age: 60.6 (0.87)<br>Males: 55%	7.2 (0.06)	NR	92.1 (1.49)	32.1 (0.41)	6.0 (0.48)
			Placebo	Age: 60.8 (0.72)<br>Males: 56%	7.2 (0.05)	NR	90.9 (1.44)	31.7 (0.41)	5.5 (0.56)
**Pan 2012 N=438**	24	HbA1c: 7-10%<br>Stable therapy for ≥4 weeks<br>Metformin Dose: ≥1500 mg/d<br>BMI: 20-40 kg/m2<br>FPG: <270 mg/dl	Vildagliptin 50 mg QD	Age: 53.7 (0.82)<br>Males: 55%	8.1 (0.07)	NR	68.4 (0.91)	25.0 (0.25)	5.0 (0.36)
			Vildagliptin 50 mg BID	Age: 54.2 (0.80)<br>Males: 50%	8.1 (0.07)	NR	71.6 (0.99)	26.0 (0.27)	4.9 (0.40)
			Placebo	Age: 54.5 (0.81)<br>Males: 56%	8.0 (0.07)	NR	69.8 (0.93)	25.5 (0.26)	5.2 (0.38)
**Rizzo 2012 N=90**	12	HbA1c: >7.5%<br>Stable therapy for ≥8 weeks<br>Metformin Dose: ≥2000 mg/d	Sitagliptin 100 mg QD	Age: 60 (1.27)<br>Males: 44%	8.5 (0.16)	129 (2.24)	NR	30 (0.85)	8.6 (0.33)
			Vildagliptin 50 mg BID	Age: 60 (1.31)<br>Males: 51%	8.2 (0.10)	132 (1.94)	NR	29.7 (0.76)	8.9 (0.28)
**Rosenstock 2012 N=451**	12	HbA1c: 7-10.5%<br>Stable therapy for ≥12 weeks<br>Metformin Dose: ≥1500 mg/d<br>BMI: 25-45 kg/m2 (or 24-45 kg/m2 for Asian patients)<br>Weight: Stable	Placebo	Age: 53.3 (0.97)<br>Males: 48%	7.8 (0.10)	125 (1.24)	85.9 (2.42)	30.6 (0.57)	6.4 (0.62)
			Canagliflozin 100 mg QD	Age: 51.7 (1.00)<br>Males: 56%	7.8 (0.12)	127 (1.63)	87.7 (1.94)	31.7 (0.63)	6.1 (0.59)
			Canagliflozin 300 mg QD	Age: 52.3(0.86)<br>Males: 56%	7.7 (0.13)	126 (1.50)	87.3 (1.99)	31.6 (0.61)	5.9 (0.65)
			Sitagliptin 100 mg QD	Age: 51.7 (1.00)<br>Males: 59%	7.6 (0.12)	129 (1.61)	87.2 (2.23)	31.6 (0.62)	5.6 (0.58)
**Ross 2012 N=491**	12	HbA1C: 7-10%<br>Stable therapy for ≥12 weeks<br>Metformin Dose: ≥1500 mg/d (or maximum tolerated dose)<br>BMI: ≤45 kg/m2	Linagliptin 2.5 mg BID	Age: 58.7 (0.66)<br>Males: 62%	8.0 (0.05)	NR	82.2 (1.15)	29.8 (0.35)	52.3% >5 years
			Linagliptin 5 mg QD	Age: 58.4 (0.71)<br>Males: 54%	8.0 (0.05)	NR	80.6 (1.17)	29.6 (0.33)	52.0% >5 years
			Placebo	Age: 59.9 (1.61)<br>Males: 48%	7.9 (0.11)	NR	77.7 (2.92)	28.7 (0.83)	55.8% >5 years
**Arechavaleta 2011 N=1035**	30	HbA1c: 6.5-9%<br>Stable therapy for ≥12 weeks<br>Metformin Dose: ≥1500 mg/d	Sitagliptin 100 mg QD	Age: 56.3 (0.43)<br>Males: 55%	7.5 (0.03)	NR	80.6 (0.67)	29.7 (0.20)	6.8 (0.20)
			Glimeperide 1 – 6 mg QD	Age: 56.2 (0.44)<br>Males: 54%	7.5 (0.04)	NR	82.0 (0.73)	30.2 (0.19)	6.7 (0.21)

**Nauck 2011 N=801**	52	HbA1c: 6.5-10%<br>Therapy with mono- or dual OAD ≥8 weeks<br>Metformin Dose: ≥1500 mg/d<br>If taking <1500 mg/d then entered an 8week dose stabilization period with 1500-2000 mg/d<br>FPG < 270.3 mg/dl	Dapagliflozin 2.5-10 mg QD	Age: 58 (0.45)<br>Males: 55%	7.7 (0.05)	132.8 (NR)	96.4 (NR)	31.7 (0.26)	6 (0.25)
			Glipizide 5-20 mg QD	Age: 59 (0.50)<br>Males: 55%	7.7 (0.05)	133.8 (NR)	96.5 (NR)	31.2 (0.25)	7 (0.30)
**Pfützner 2011 N=288**	24	HbA1c: ≥6.5-9%<br>Stable therapy for ≥12 weeks<br>Metformin Dose: Maximally tolerated dose (850-2000 mg)	Pioglitazone 15 mg BID	Age: 59 (0.83)<br>Males: 66%	7.1 (0.05)	138 (1.24)	96.2 (1.45)	32.6 (0.41)	6.2 (0.45)
			Glimepiride 1 mg BID	Age: 59 (0.84)<br>Males: 64%	7.4 (0.06)	137 (1.09)	94.1 (1.51)	32.5 (0.44)	5.9 (0.40)
**Taskinen 2011 N=700**	24	HbA1c: 7-10%<br>Stable therapy for ≥12 weeks<br>Metformin Dose: ≥1500 mg/d (or maximum tolerated dose)<br>BMI ≤40 kg/m2	Placebo	Age: 56.6 (0.82)<br>Males: 57%	8.0 (0.07)	NR	83.3 (1.25)	30.1 (0.38)	53% >5 years
			Linagliptin 5 mg QD	Age: 56.5 (0.44)<br>Males: 53%	8.1 (0.04)	NR	82.2 (0.75)	29.9 (0.21)	56% >5 years
**Wang 2011 N=55**	16	HbA1c: 7-11%;<br>Therapy with mono- or dual OAD ≥12 weeks<br>Metformin Dose: 1500 mg/d<br>8week monotherapy stabilization period	Acarbose 100 mg TID	Age: 52.8 (1.55)<br>Males: 53.6%	8.2 (0.15)	129 (3.21)	69.8 (1.87)	25.9 (0.57)	7.6 (NR)
			Glibenclamide 5 mg TID	Age: 54.7 (1.73)<br>Males: 44%	8.6 (0.33)	128 (3.34)	66.0 (3.21)	25.3 (0.79)	6.0 (NR)
**Yang 2011 N=570**	24	HbA1c: 7-10%<br>Stable therapy for ≥8 weeks<br>Metformin Dose: ≥1500 mg/d	Saxagliptin 5 mg QD	Age: 53.8 (0.62)<br>Males: 48%	7.9 (0.05)	NR	68.9 (0.74)	26.3 (0.21)	5.1 (0.30)
			Placebo	Age: 54.4 (0.60)<br>Males: 48%	7.9 (0.05)	NR	69.0 (0.70)	26.1 (0.21)	5.1 (0.24)
**Bailey 2010 N=546**	24	HbA1c: 7-10%<br>Stable therapy for ≥8 weeks<br>Metformin Dose: ≥1500 mg/d<br>BMI: ≤45 kg/m2	Placebo	Age: 53.7 (0.88)<br>Males: 55%	8.1 (0.08)	127.7 (1.25)	87.7 (1.64)	31.8 (0.45)	5.8 (0.44)
			Dapagliflozin 5 mg	Age: 54.3 (0.80)<br>Males: 50%	8.2 (0.08)	126.9 (1.22)	84.7 (1.39)	31.4 (0.43)	6.4 (0.50)
			Dapagliflozin 10 mg QD	Age: 52.7 (0.85)<br>Males: 57%	7.9 (0.07)	126.0 (1.37)	86.3 (1.50)	31.2 (0.44)	6.1 (0.46)
**Filozof 2010 N=1007**	52	HbA1c: 7.5-11%<br>Stable therapy for ≥4 weeks<br>Metformin Dose: ≥1500 mg/d	Vildagliptin 50 mg BID	Age: 59.2 (0.44)<br>Males: 52%	8.5 (0.04)	NR	85.7 (0.73)	31.2 (0.22)	6.4 (0.23)
			Gliclazide 80-320 mg QD	Age: 59.7 (0.46)<br>Males: 52%	8.5 (0.04)	NR	84.2 (0.81)	30.8 (0.22)	6.8 (0.24)
**Goke 2010 N=858**	52	HbA1c: 6.5-10%<br>Stable therapy for ≥8 weeks<br>Metformin Dose: ≥1500 mg/d	Saxagliptin 5 mg QD	Age: 57.5 (0.50)<br>Males: 50%	7.7 (0.04)	NR	88.7 (0.90)	31.5 (0.28)	5.5 (0.22)
			Glipizide 5-20 mg QD	Age: 57.6 (0.50)<br>Males: 54%	7.7 (0.04)	NR	88.6 (0.95)	31.3 (0.30)	5.4 (0.23)
**Pratley 2010 N=665**	26	HbA1c:7.5-10%<br>Stable therapy for ≥12 weeks<br>Metformin Dose: ≥1500 mg/d<br>BMI: ≤45 kg/m2	Liraglutide 1.2 mg QD	Age: 55.9 (0.6)<br>Males: 52%	8.4 (0.05)	131.2 (0.96)	93.7 (1.23)	32.6 (0.35)	6.0 (0.30)
			Liraglutide 1.8 mg QD	Age: 55.0 (0.6)<br>Males: 52%	8.4 (0.05)	133.4 (0.98)	94.6 (1.22)	33.1 (0.34)	6.4 (0.36)
			Sitagliptin 100 mg QD	Age: 55.0 (0.6)<br>Males: 55%	8.5 (0.05)	132.1 (1.00)	93.1 (1.28)	32.6 (0.36)	6.3 (0.36)

**Rigby 2010 N=169**	16	HbA1C: 7-10%<br>Stable therapy for ≥12 weeks<br>Metformin Dose: 1500-2550 mg/d<br>BMI: ≤40 kg/m2	Colesvelam 3.75 g QD	Age: 56.6 (1.22)<br>Males: 49%	8.1 (0.10)	NR	78.7 (2.45)	NR	8.1 (0.79)
			Rosiglitazone 4 mg QD	Age: 54.7 (1.46)<br>Males: 41%	8.1 (0.10)	NR	81.1 (2.39)	NR	7.6 (0.80)
			Sitagliptin 100 mg QD	Age: 54.8 (1.31)<br>Males: 36%	8.2 (0.12)	NR	79.6 (2.42)	NR	8.4 (0.94)
**Scheen 2010 N=801**	18	HbA1C: 6.5-10%<br>Stable therapy for ≥8 weeks<br>Metformin Dose: ≥1500 mg/d	Saxagliptin 5 mg QD	Age: 58.8 (0.50)<br>Males: 47%	7.7 (0.05)	NR	NR	31.1 (0.26)	6.3 (0.25)
			Sitagliptin 100 mg QD	Age: 58.1 (0.53)<br>Males: 51%	7.7 (0.05)	NR	NR	30.9 (0.28)	6.3 (0.24)
**DeFronzo 2009 N=743**	24	HbA1C: 7-10%<br>Stable therapy for ≥8 weeks<br>Metformin Dose: 1500-2550 mg/d<br>BMI: ≤40 kg/m2	Placebo	Age: 54.8 (0.76)<br>Males: 54%	8.1 (0.07)	NR	87.1 (1.33)	31.6 (0.36)	6.7 (0.42)
			Saxagliptin 2.5 mg QD	Age: 54.7 (0.73)<br>Males: 43%	8.1 (0.07)	NR	86.0 (1.27)	31.7 (0.38)	6.7 (0.40)
			Saxagliptin 5 mg QD	Age: 54.7 (0.69)<br>Males: 54%	8.1 (0.06)	NR	87.3 (1.23)	31.2 (0.34)	6.4 (0.34)
**Ferrannini 2009 N=2789**	52	HbA1c: 6.5-8.5%<br>Stable therapy for ≥4 weeks<br>Metformin Dose: ≥1500 mg/d<br>BMI: 22-45 kg/m2	Vildagliptin 50mg BID	Age: 57.5 (0.24)<br>Males: 53%	7.3 (0.02)	NR	89.0 (NR)	31.8 (0.14)	5.7 (0.14)
			Glimeperide 2 to 6 mg QD	Age: 57.5 (0.25)<br>Males: 54%	7.3 (0.02)	NR	88.6 (NR)	31.7 (0.14)	5.8 (0.13)
**Goodman 2009 N=370**	24	HbA1c: 7.5-11%<br>Stable therapy for ≥12 weeks<br>Metformin Dose: ≥1500 mg<br>BMI: 22-40 kg/m2<br>FPG: <270 mg/dl	Vildagliptin 100 mg QD	Age: 54.9 (0.69)<br>Males: 53%	8.5 (0.06)	NR	NR	31.4 (0.30)	NR
			Placebo	Age: 54.5 (0.88)<br>Males: 67%	8.7 (0.10)	NR	NR	31.7 (0.39)	NR
**Nauck 2009 N=527**	26	HbA1c: 7-10%<br>Stable therapy for ≥12 weeks<br>Metformin Dose: ≥1500 mg/d<br>BMI: 23-45 kg/m2<br>FPG: <275 mg/dl	Placebo	Age: 56 (1.08)<br>Males: 48%	8.0 (0.09)	129 (1.67)	NR	32 (0.59)	6 (0.49)
			Alogliptin 12.5 mg QD	Age: 55 (0.75)<br>Males: 47%	7.9 (0.05)	127 (0.89)	NR	32 (0.34)	6 (0.34)
			Alogliptin 25 mg QD	Age: 54 (0.76)<br>Males: 54%	7.9 (0.06)	127.3 (1.03)	NR	32 (0.35)	6 (0.28)
**Nauck 2009 N=1087**	26	HbA1c: 7-11%<br>Therapy with mono- or dual OAD ≥12 weeks<br>3 week metformin titration to 2 g/d then a 3 week stabilization period<br>BMI: ≤40 kg/m2	Liraglutide 0.6 mg QD	Age: 56 (0.71)<br>Males: 62%	8.4 (0.06)	131 (0.90)	NR	30.5 (0.31)	7 (0.32)
			Liraglutide 1.2 mg QD	Age: 57 (0.58)<br>Males: 54%	8.3 (0.06)	132 (0.90)	NR	31.1 (0.31)	7 (0.32)
			Liraglutide 1.8 mg QD	Age: 57 (0.58)<br>Males: 59%	8.4 (0.06)	131 (0.90)	NR	30.9 (0.30)	8 (0.32)
			Glimepiride 4 mg QD	Age: 57 (0.58)<br>Males: 57%	8.4 (0.06)	132 (1.03)	NR	31.2 (0.30)	8 (0.32)
			Placebo	Age:56 (0.82)<br>Males: 60%	8.4 (0.10)	135 (1.45)	NR	31.6 (0.40)	8 (0.55)

**Hamann 2008 N=596**	52	HbA1c: 7-10%<br>Stable therapy for ≥8 weeks<br>Metformin Dose: ≥850 mg/d<br>If taking ≥1.5 g/d entered a 4 week titration with 2 g/d.<br>If taking <1.5 g/d entered an additional 1 week titration with 1.5 g/d before entering 4 week titration with 2 g/d<br>BMI: ≥25 kg/m2	Rosiglitazone/metformin 4 mg QD	Age: 58.5 (0.56)<br>Males: 53%	8.0 (0.05)	NR	91.4 (1.00)	33.0 (0.34)	6.3 (0.31)
			Glibenclamide 5 mg QD	Age: 59.3 (0.53)<br>Males: 52%	8.0 (0.06)	NR	88.9 (0.96)	32.2 (0.28)	6.4 (0.32)
			Gliclazide 80 mg QD						
**Khanolkar 2008 N=50**	24	HbA1c: >6.5%<br>If <2 g/d metformin, entered 4week titration, when max tolerated dose was reached (up to 2 g/d) then entered a 4week stabilization	Rosiglitazone 4 mg QD	Age: 59 (NR)<br>Males: 56%	7.3 (0.10)	NR	NR	34.6 (1.77)	NR
			Gliclazide 80 mg QD	Age: 56 (NR)<br>Males: 60%	7.1 (0.14)	NR	NR	33.7 (1.64)	NR
**Raz 2008 N=190**	30	HbA1c: 8-11%<br>Therapy with mono- or dual OAD. OAD(s) switched to metformin 1500-2550 mg/d and entered a 6 week stabilization<br>FPG: 129.7-281.1 mg/dl	Sitagliptin 100 mg QD	Age: 53.6 (0.97)<br>Males: 51%	9.3 (0.09)	NR	81.5 (1.71)	30.1 (0.45)	8.4 (0.66)
			Placebo	Age: 56.1 (0.98)<br>Males: 42%	9.1 (0.08)	NR	81.2 (2.00)	30.4 (0.55)	7.3 (0.55)
**Scott 2008 N=273**	18	HbA1c: 7-11%<br>Stable therapy for ≥10 weeks<br>Metformin Dose: ≥1500 mg/d<br>FPG: <270 mg/dl	Placebo	Age: 55.3 (0.97)<br>Males: 59%	7.7 (0.09)	NR	84.6 (1.72)	30.0 (0.47)	5.4 (0.39)
			Sitagliptin 100 mg QD	Age: 55.2 (1.01)<br>Males: 55%	7.8 (0.10)	NR	83.1 (1.76)	30.3 (0.48)	4.9 (0.36)
			Rosiglitazone 8 mg QD	Age: 54.8 (1.13)<br>Males: 63%	7.7 (0.09)	NR	84.9 (1.98)	30.4 (0.59)	4.6 (0.43)
**Bosi 2007 N=544**	24	HbA1c: 7.5-11%<br>Stable therapy for ≥4 weeks<br>Metformin Dose: ≥1500 mg/d<br>If tolerated, increased to 2 g/d at visit 1<br>BMI: 22-45 kg/m2<br>FPG: < 270.3 mg/dl	Vildagliptin 50 mg QD	Age: 54.3 (0.73)<br>Males: 57%	8.4 (0.07)	NR	NR	32.1 (0.40)	6.8 (0.41)
			Vildagliptin 100 mg QD	Age: 53.9 (0.70)<br>Males: 62%	8.4 (0.07)	NR	NR	32.9 (0.37)	5.8 (0.35)
			Placebo	Age: 54.5 (0.76)<br>Males: 53%	8.3 (0.07)	NR	NR	33.2 (0.45)	6.2 (0.39)
**Nauck 2007 N=1172**	52	HbA1c: 6.5-10%<br>Metformin Dose: ≥1.5 g/d<br>If not on a OAD, or on dual-OAD then entered a metformin monotherapy titration and stabilization for 8weeks	Sitagliptin 100 mg QD	Age: 56.8 (0.38)<br>Males: 57%	7.7 (0.04)	NR	89.5 (0.72)	31.2 (0.21)	6.5 (0.25)
			Glipizide 5 – 20 mg QD	Age:56.9 (0.41)<br>Males: 61%	7.6 (0.04)	NR	89.7 (0.72)	31.3 (0.22)	6.2 (0.22)
**Ristic 2006 N=262**	24	HbA1c: 6.8-9%<br>Stable therapy for ≥8 weeks<br>Metformin Dose: ≥1000 mg/d (or maximum tolerated dose)<br>BMI: 20-35 kg/m2	Nateglinide 60 – 180 mg TID	Age: 62.0 (0.95)<br>Males: 54%	7.7 (0.05)	NR	NR	28.5 (0.30)	7.2 (0.55)
			Gliclazide 80 – 240 mg QD	Age: 61.6 (0.89)<br>Males: 50%	7.6 (0.05)	NR	NR	29.5 (0.32)	6.7 (0.49)

**DeFronzo 2005 N=336**	30	HbA1c: 7.1-11%<br>Stable therapy for ≥12 weeks<br>Metformin Dose: ≥1500 mg/d<br>BMI: 27-45 kg/m2<br>FPG: <240 mg/dl<br>Weight: Stable (<10% variance) for ≥12 weeks before screening	Placebo	Age: 54 (0.84)<br>Males: 59%	8.2 (0.09)	NR	100 (1.79)	34 (0.56)	6.6 (0.57)
			Exenatide 5 ug BID	Age: 53 (1.05)<br>Males: 52%	8.3 (0.10)	NR	100 (2.10)	34 (0.57)	6.2 (0.56)
			Exenatide 10 ug BID	Age: 52 (1.03)<br>Males: 60%	8.2 (0.09)	NR	101 (1.88)	34 (0.56)	4.9 (0.44)
**Feinglos 2005 N=122**	16	HbA1c: 7-8.5%<br>Stable therapy for ≥12 weeks<br>Metformin Dose: ≥1 g/d<br>BMI: 27-38 kg/m2	Glipizide 2.5 mg QD	Age: 57.7 (1.37)<br>Males: 46%	7.5 (NR)	NR	90.0 (2.39)	31.7 (0.56)	6.5 (NR)
			Placebo	Age: 58.8 (1.28)<br>Males: 41%	7.6 (NR)	NR	90.8 (2.36)	32.1 (0.63)	4.6 (NR)
**Matthews 2005 N=630**	52	HbA1c: 7.5-11%<br>Stable therapy for ≥12 weeks<br>Metformin Dose: ≥50% maximum recommended dose or maximum tolerated dose	Pioglitazone 15 – 45 mg QD	Age: 56 (0.52)<br>Males: 51%	8.7 (0.06)	NR	91.8 (0.91)	32.6 (0.28)	5.8 (0.29)
			Gliclazide 80 – 320 mg QD	Age:57 (0.51)<br>Males: 49%	8.5 (0.05)	NR	92.7 (0.98)	32.6 (0.33)	5.5 (0.29)
**Ahren 2004 N=71**	12	HbA1c: 7-9.5%<br>Stable therapy for ≥12 weeks<br>Metformin Dose: 1.5-3 g/d<br>BMI: 20-35 kg/m2	Vildagliptin 50 mg QD	Age: 58.4 (1.42)<br>Males: 62%	7.6 (0.09)	NR	NR	29.6 (0.57)	5.8 (0.65)
			Placebo	Age: 54.3 (2.27)<br>Males: 76%	7.8 (0.11)	NR	NR	29.9 (0.67)	4.6 (0.67)
**Gomez-Perez 2002 N=116**	26	FPG: 140-300 mg/dl<br>3-6 week titration with 2.5 g/d then 4 week stabilization period	Placebo	Age: 53.4 (1.29)<br>Males: 29%	NR	NR	NR	28.5 (0.67)	9.1 (0.96)
			Rosiglitazone 2 mg QD	Age: 51.7 (1.45)<br>Males: 29%	NR	NR	NR	28.0 (0.68)	11.1 (1.20)
			Rosiglitazone 4 mg QD	Age: 54.2 (1.55)<br>Males: 19%	NR	NR	NR	27.6 (0.53)	10.7 (1.17)
**Marre 2002 N=467**	24	HbA1c: 6.8-11%<br>Stable therapy for ≥4 weeks<br>Metformin Dose: ≥1500 mg/d<br>4 week 2 g/d stabilization period<br>BMI: 20-35 kg/m2	Nateglinide 60 mg TID	Age: 57.9 (0.80)<br>Males: 61%	8.0 (NR)	NR	84.8 (1.1)	29.4 (0.30)	7.2 (0.51)
			Nateglinide 120 mg TID	Age: 57.3 (0.83)<br>Males: 61%	8.2 (NR)	NR	85.2 (1.1)	29.3 (0.28)	6.8 (0.43)
			Placebo	Age: 56.4 (0.84)<br>Males: 55%	8.3 (NR)	NR	84.9 (1.2)	29.6 (0.32)	6.5 (0.53)
**Charpentier 2001 N=372**	20	Stable therapy for ≥4 weeks<br>Metformin Dose: 2550 mg/d<br>FPG: 7.8-13.9 mmol/l<br>BMI: 23-40 kg/m2	Placebo	Age: 56.7 (NR)<br>Males: 60%	6.8 (0.14)	142 (1.27)	82.2 (NR)	29.2 (NR)	7.0 (NR)
			Glimepiride 1 – 6 mg QD	Age: 56.8 (NR)<br>Males: 59%	6.4 (0.09)	140 (0.99)	81.2 (NR)	29.5 (NR)	5.6 (NR)
**Van Gaal 2001 N=152**	32	HbA1c: 7.5-10.5%<br>Stable therapy for ≥12 weeks<br>Metformin Dose: 1500-2550 mg/d<br>BMI: 23-40 kg/m2<br>Weight: Stable (<5% variance) for ≥12 weeks before enrollment	Miglitol 100 mg TID	Age: 57.9 (1.14)<br>Males: 42%	8.5 (0.11)	NR	NR	30.0 (0.46)	6 (1.32)[c]
			Placebo	Age: 57.9 (0.98)<br>Males: 49%	8.4 (0.12)	NR	NR	29.7 (0.45)	6 (0.22)[c]

**Halimi 2000 N= 129**	24	HbA1c: 7-11%<br>Stable therapy for ≥8 weeks<br>Metformin Dose: 1500-2550 mg/d<br>BMI: 25-35 kg/m2	Acarbose 50-100 mg TID	Age: 56 (1.20)<br>Males: 48%	8.6 (0.14)	NR	NR	30.1 (0.43)	9.5 (0.97)
			Placebo	Age: 55 (1.20)<br>Males: 63%	8.5 (0.13)	NR	NR	29.7 (0.39)	9.0 (0.90)
**Fonseca 2000 N=348**	26	Metformin Dose: ≥2.5 g/d<br>If <2.5 g/d, entered a 3 week titration to 2.5 g/d then 4 week stabilization period<br>BMI 22-38 kg/m2<br>Weight: Stable (<10% variance) between screening and baseline<br>FPG: 140-300 mg/dl	Placebo	Age: 58.8 (0.87)<br>Males: 74%	8.6 (0.12)	NR	NR	30.3 (0.41)	7.3 (0.54)
			Rosiglitazone 4 mg QD	Age: 57.5 (0.97)<br>Males: 62%	8.9 (0.12)	NR	NR	30.2 (0.39)	7.5 (0.58)
			Rosiglitazone 8 mg QD	Age: 58.3 (0.84)<br>Males: 68%	8.9 (0.14)	NR	NR	29.8 (0.37)	8.3 (0.60)
**Moses 1999 N=83**	12	HbA1c: >7.1%<br>Stable therapy: ≥6 months<br>Metformin Dose: 1-3 g/d<br>BMI: ≥21 kg/m2	Repaglinide 4 mg QD	Age: 57.2 (1.60)<br>Males: 67%	8.3 (0.17)	NR	NR	33.2 (1.08)	5.9 (0.56)
			Placebo	Age: 57.8 (1.83)<br>Males: 63%	8.6 (0.21)	NR	NR	31.8 (1.15)	8.0 (1.19)
**Rosenstock 1998 N=148**	24	HbA1c: 7-10%<br>Stable therapy for ≥8 weeks<br>Metformin Dose: 2 or 2.5 g/d<br>Weight: Stable (< 3 kg) for ≥4 weeks before screening	Acarbose 50-100 mg TID	Age: 57.2 (NR)<br>Males: 61%	8.5 (NR)	NR	94.4 (NR)	32.4 (NR)	7.2 (NR)
			Placebo	Age: 55.9 (NR)<br>Males: 49%	8.2 (NR)	NR	91.5 (NR)	32.3 (NR)	7.8 (NR)
