# Supplementary material for: Comparative Efficacy and Safety of Antidiabetic Drug Regimens Added to Metformin Monotherapy in Patients with Type 2 Diabetes: A Network Meta-Analysis
Source: PLoS One. 2015 Apr 28;10(4):e0125879. doi: 10.1371/journal.pone.0125879 (PMC4412636; doi:10.1371/journal.pone.0125879)
Supplement: S2 Table — ACA = acarbose; ALO = alogliptin; ALO/PIO = alogliptin/pioglitazone; CANA = canagliflozin; COL = colesevelam; DAPA = dapagliflozin; EMPA = empagliflozin; EMPA/LINA = empagliflozin/linagliptin; EXEN = exenatide; GLAR = insulin glargine; GLIB = glibenclamide; GLIC = gliclazide; GLIM = glimepiride; GLIP = glipizide; LINA = linagliptin; LIRA = liraglutide; LIX = lixisenatide; MIG = miglitol; NAT = nateglinide; PIO = pioglitizone; PLC = placebo; REP = repaglinide; ROSI = rosiglitizone; SAX = saxagliptin; SITA = sitagliptin; VILDA = vildagliptin. aI2>50%. (PDF) [file pone.0125879.s019.pdf]

**Table S2. Results of Traditional Meta-Analysis Comparing Antidiabetic Therapies Effect on HbA1c**

| Comparison | No. of Trials | Change in HbA1c, % WMD (95%CI) |
|---|---|---|
| ACA vs. PLC | 2 | -0.78 (-1.06, -0.50) |
| ALO vs.  PLC | 2 | -0.60 (-0.76, -0.43) |
| ALO/PIO vs. PLC | 1 | -1.29 (-1.49, -1.09) |
| CANA vs. PLC | 2 | -0.67 (-0.79, -0.55) |
| DAPA vs. PLC | 2 | -0.43 (-0.55, -0.31) |
| EMPA vs. PLC | 2 | -0.64 (-0.75, -0.52) |
| EXEN vs. PLC | 2 | -0.79 (-0.86, -0.72) |
| GLIM vs. PLC | 2 | -0.95 (-1.23, -0.67) |
| GLIP vs. PLC | 1 | -0.47 (-0.74, -0.20) |
| LINA vs. PLC | 2 | -0.68 (-0.81, -0.54) |
| LIRA vs. PLC | 1 | -1.00 (-1.26, -0.75) |
| LIX vs. PLC | 1 | -0.45 (-0.69, -0.22) |
| MIG vs. PLC | 1 | -0.43 (-0.70, -0.16) |
| NAT vs. PLC | 1 | -0.44 (-0.60, -0.28) |
| PIO vs. PLC | 1 | -0.76 (-0.98, -0.54) |
| REP vs. PLC | 1 | -1.08 (-1.73, -0.43) |
| ROSI vs. PLC | 3 | -0.91 (-1.38, -0.44)[a] |
| SAXA vs. PLC | 3 | -0.50 (-0.74, -0.26)[a] |
| SITA vs. PLC | 8 | -0.67 (-0.73, -0.60) |
| VILDA vs. PLC | 5 | -0.63 (-0.83, -0.43)[a] |
| ACA vs. GLIB | 1 | 0.50 (-0.13, 1.13) |
| ALO vs. ALO/PIO | 1 | 0.65 (0.49, 0.81) |
| ALO vs. PIO | 1 | 0.12 (-0.06, 0.30) |
| CANA vs. GLIM | 1 | -0.06 (-0.16, 0.04) |
| CANA vs. SITA | 2 | -0.05 (-0.14, 0.04) |
| COL vs. ROSI | 1 | 0.30 (-0.05, 0.65) |
| COL vs. SITA | 1 | 0.10 (-0.25, 0.45) |
| DAPA vs. GLIP | 1 | 0.00 (-0.12, 0.12) |
| EMPA vs. GLIM | 1 | -0.07 (-0.15, 0.01) |
| EMPA vs. LINA | 1 | 0.06 (-0.08, 0.20) |
| EMPA vs.  SITA | 1 | -0.11 (-0.33, 0.11) |
| EMPA/LINA vs. EMPA | 1 | -0.49 (-0.61, -0.37) |
| EMPA/LINA vs. LINA | 1 | -0.43 (-0.57, -0.29) |
| EXEN vs. GLIM | 1 | -0.18 (-0.30, -0.06) |
| LINA vs. GLIM | 1 | 0.21 (0.13, 0.29) |
| LIRA vs. GLIM | 1 | 0.10 (-0.10,  0.30) |
| LIRA vs. SITA | 2 | -0.28 (-0.64, 0.08) |
| LIX vs. EXEN | 1 | 0.17 (0.03, 0.31) |
| NAT vs. GLIC | 1 | 0.16 (-0.06, 0.38) |
| PIO vs.  ALO/PIO | 1 | 0.53 (0.39, 0.67) |
| PIO vs. GLIC | 1 | 0.02 (-0.16, 0.20) |
| PIO vs. GLIM | 1 | 0.12 (-0.08, 0.32) |
| ROSI vs. GLIC | 2 | -0.01 (-0.27, 0.24) |
| SAX vs. GLIP | 1 | 0.08 (-0.06, 0.18) |
| SAX vs. SITA | 1 | 0.10 (-0.02, 0.22) |
| SITA vs. GLARG | 1 | 0.59 (0.41, 0.77) |
| SITA vs. GLIM | 1 | 0.07 (-0.05, 0.19) |
| SITA vs. GLIP | 1 | 0.00 (-0.12, 0.12) |
| SITA vs. PIO | 1 | 0.09 (-0.07, 0.25) |
| SITA vs. ROSI | 2 | 0.09 (-0.08, 0.26) |
| SITA vs. VILDA | 1 | -0.20 (-0.53, 0.13) |
| VILDA vs. GLIC | 1 | 0.04 (-0.12, 0.20) |
| VILDA vs. GLIM | 2 | 0.08 (0.03, 0.14) |
