# Supplementary material for: Comparative Efficacy and Safety of Antidiabetic Drug Regimens Added to Metformin Monotherapy in Patients with Type 2 Diabetes: A Network Meta-Analysis
Source: PLoS One. 2015 Apr 28;10(4):e0125879. doi: 10.1371/journal.pone.0125879 (PMC4412636; doi:10.1371/journal.pone.0125879)
Supplement: S3 Table — ACA = acarbose; ALO = alogliptin; ALO/PIO = alogliptin/pioglitazone; CANA = canagliflozin; COL = colesevelam; DAPA = dapagliflozin; EMPA = empagliflozin; EMPA/LINA = empagliflozin/linagliptin; EXEN = exenatide; GLAR = insulin glargine; GLIB = glibenclamide; GLIC = gliclazide; GLIM = glimepiride; GLIP = glipizide; LINA = linagliptin; LIRA = liraglutide; LIX = lixisenatide; MIG = miglitol; NAT = nateglinide; PIO = pioglitizone; PLC = placebo; REP = repaglinide; ROSI = rosiglitizone; SAX = saxagliptin; SITA = sitagliptin; VILDA = vildagliptin. aI2>50%. (PDF) [file pone.0125879.s020.pdf]

Table S3. Results of Traditional Meta-Analysis Comparing Antidiabetic Therapies Effect on Body Weight

Comparison	No. of Trials	Change in Weight, kg WMD (95%CI)
ACA vs. PLC	1	-0.10 (-0.92, 0.72)
ALO vs. PLC	2	0.05 (-0.41, 0.51)
ALO/PIO vs. PLC	1	2.50 (1.74, 3.26)
CANA vs. PLC	2	-2.10 (-2.65, -1.55)
DAPA vs. PLC	2	-2.10 (-2.62, -1.59)
EMPA vs. PLC	2	-1.74 (-2.10, -1.37)
EXEN vs. PLC	2	-2.76 (-4.85, -0.67)
GLIM vs. PLC	2	1.92 (0.78, 3.06)
GLIP vs. PLC	1	2.10 (0.85, 3.35)
LINA vs. PLC	1	0.40 (-0.50, 1.30)
LIRA vs. PLC	1	-0.90 (-1.49, -0.31)
LIX vs. PLC	1	-1.02 (-1.96, -0.08)
MIG vs. PLC	1	-1.80 (-2.78, -0.82)
NAT vs. PLC	1	0.60 (0.13, 1.07)
PIO vs. PLC	1	2.14 (1.32, 2.96)
REP vs. PLC	1	3.27 (1.88, 4.66)
ROSI vs. PLC	3	2.27 (1.84, 2.70)
SAXA vs. PLC	2	-0.06 (-0.32, 0.20)
SITA vs. PLC	7	0.16 (-0.12, 0.45)
VILDA vs. PLC	4	-0.16 (-1.02, 0.70)[a]
ACA vs. GLIB	1	-2.30 (-3.53, -1.07)
ALO/PIO vs. ALO	1	2.19 (1.62, 2.76)
ALO/PIO vs. PIO	1	0.36 (-0.15, 0.87)
CANA vs. GLIM	1	-4.55 (-5.02, -4.08)
CANA vs. SITA	2	-2.28 (-2.67, -1.89)
COL vs. ROSI	1	-0.90 (-2.04, 0.24)
COL vs. SITA	1	0.51 (-0.31, 1.33)
DAPA vs. GLIP	1	-4.66 (-5.15, -4.17)
EMPA vs. GLIM	1	-4.80 (-5.07, -4.53)
EMPA vs. LINA	1	-2.15 (-2.88, -1.42)
EMPA vs. SITA	1	-1.85 (-2.61, -1.09)
EMPA/LINA vs. EMPA	1	0.05 (-0.54, 0.64)
EMPA/LINA vs. LINA	1	-2.10 (-2.83, -1.37)
LINA vs. GLIM	1	-2.50 (-2.87, -2.13)
LIRA vs. GLIM	1	-3.40 (-3.83, -2.97)
LIRA vs. SITA	2	-2.30 (-2.76, -1.85)
LIX vs. EXEN	1	1.02 (0.37, 1.67)
PIO vs. ALO	1	1.83 (1.20, 2.46)
PIO vs. GLIM	1	0.00 (-1.00, 1.00)
ROSI vs. GLIC	1	1.10 (0.28, 1.92)
ROSI vs. SITA	2	1.81 (1.31, 2.30)
SAX vs. GLIP	1	-2.20 (-2.69, -1.71)
SITA vs. GLARG	1	-1.52 (-2.11, -0.93)
SITA vs. GLIM	1	-2.00 (-2.41, -1.59
SITA vs. GLIP	1	-2.60 (-3.38, -1.82)
VILDA vs. GLIC	1	-1.28 (-2.04, -0.52)
VILDA vs. GLIM	2	-1.81 (-2.12, -1.50)
