# Supplementary material for: Comparative Efficacy and Safety of Antidiabetic Drug Regimens Added to Metformin Monotherapy in Patients with Type 2 Diabetes: A Network Meta-Analysis
Source: PLoS One. 2015 Apr 28;10(4):e0125879. doi: 10.1371/journal.pone.0125879 (PMC4412636; doi:10.1371/journal.pone.0125879)
Supplement: S4 Table — CANA = canagliflozin; DAPA = dapagliflozin; EMPA = empagliflozin; EMPA/LINA = empagliflozin/linagliptin; EXEN = exenatide; GLAR = insulin glargine; GLIM = glimepiride; GLIP = glipizide; LINA = linagliptin; LIRA = liraglutide; PIO = pioglitizone; PLC = placebo; SAX = saxagliptin; SBP = systolic blood pressure; SITA = sitagliptin; VILDA = vildagliptin. aEgger’s p-value <0.05 (PDF) [file pone.0125879.s021.pdf]

Table S4. Results of Traditional Meta-Analysis Comparing Antidiabetic Therapies Effect on Systolic Blood Pressure

Comparison	No. of Trials	Change in SBP, mmHg WMD (95%CI)
CANA vs. PLC	2	-3.52 (-8.74, 1.70)
DAPA vs. PLC	1	-4.5 (-7.54, -1.46)
EMPA vs. PLC	2	-4.41 (-5.96, -2.87)
GLIM vs. PLC	1	0.51 (-3.08, 4.10)
SAX vs. PLC	2	0.64 (-1.86, 3.13)
SITA vs. PLC	4	-2.11 (-3.90, -0.32)[a]
CANA vs. GLIM	1	-4.15 (-5.58, -2.72)
CANA vs. SITA	2	-2.44 (-3.77, -1.11)
DAPA vs. GLIP	1	-5.00 (-6.65, -3.35)
EMPA vs. GLIM	1	-5.8 (-7.03, -4.57)
EMPA vs. LINA	1	-3.56 (-5.76, -1.36)
EMPA vs. SITA	1	-4.65 (-8.22, -1.08)
EMPA/LINA vs. EMPA	1	-0.29 (-2.07, 1.49)
EMPA/LINA vs. LINA	1	-3.85 (-6.05, -1.65)
EXEN vs. GLIM	1	-3.10 (-5.00, -1.2)
GLIM vs. LIRA	1	2.96 (1.12, 4.80)
GLIM vs. PIO	1	3.00 (-0.31, 6.31)
LIRA vs. SITA	2	-1.29 (-4.33, 1.76)
VILDA vs. SITA	1	-2.00 (-8.53, 4.53)
