# Supplementary material for: Comparative Efficacy and Safety of Antidiabetic Drug Regimens Added to Metformin Monotherapy in Patients with Type 2 Diabetes: A Network Meta-Analysis
Source: PLoS One. 2015 Apr 28;10(4):e0125879. doi: 10.1371/journal.pone.0125879 (PMC4412636; doi:10.1371/journal.pone.0125879)
Supplement: S5 Table — ACA = acarbose; ALO = alogliptin; CANA = canagliflozin; DAPA = dapagliflozin; EMPA = empagliflozin; EMPA/LINA = empagliflozin/linagliptin; EXEN = exenatide; GLAR = insulin glargine; GLIC = gliclazide; GLIM = glimepiride; GLIP = glipizide; LINA = linagliptin; LIRA = liraglutide; LIX = lixisenatide; NAT = nateglinide; PIO = pioglitizone; PLC = placebo; REP = repaglinide; ROSI = rosiglitizone; SAX = saxagliptin; SITA = sitagliptin; VILDA = vildagliptin. (PDF) [file pone.0125879.s022.pdf]

Table S5. Results of Traditional Meta-Analysis Comparing Antidiabetic Therapies Effect on Experiencing Confirmed Hypoglycemia

Comparison	No. of Trials	Relative Risk (95%CI)
ACA vs. PLC	1	0.51 (0.05, 5.47)
ALO vs. PLC	1	0.16 (0.03, 0.97)
CANA vs. PLC	2	1.55 (0.43, 5.62)
DAPA vs. PLC	2	1.50 (0.54, 4.18)
EMPA vs. PLC	1	3.45 (0.43, 27.86)
EXEN vs. PLC	1	0.93 (0.35, 2.45)
GLIM v PLC	2	2.19 (0.42,11.50)
GLIP vs. PLC	1	4.5 (1.01, 19.98)
LINA vs. PLC	2	0.30 (0.09, 0.97)
LIRA vs. PLC	1	0.88 (0.31, 2.51)
LIX vs. PLC	1	3.48 (0.43, 28.03)
NAT vs. PLC	1	2.41 (0.28, 20.47)
REP vs. PLC	1	19.00 (1.16, 310.66)
ROSI vs. PLC	2	1.35 (0.37, 4.90)
SAXA vs. PLC	2	0.68 (0.10, 4.61)
SITA vs. PLC	5	1.19 (0.31, 4.56)
VILDA vs. PLC	4	1.79 (0.43, 7.45)
CANA vs. GLIM	1	0.15 (0.11, 0.20)
CANA vs. SITA	1	17.44 (1.05, 289.20)
DAPA vs. GLIP	1	0.05 (0.02, 0.10)
EMPA vs. GLIM	1	0.08 (0.04, 0.14)
EMPA vs. LINA	1	1.41 (0.29, 6.89)
EMPA vs. SITA	1	0.10 (0.00, 2.08)
EMPA/LINA vs. EMPA	1	0.44 (0.13, 1.54)
EMPA/LINA vs. LINA	1	0.97 (0.18, 5.21)
EXEN vs. GLIM	1	0.49 (0.43, 0.57)
EXEN vs. LIX	1	3.14 (1.44, 6.87)
GLAR vs. SITA	1	5.2 (2.86, 9.46)
GLIC vs. NAT	1	1.03 (0.65, 1.64)
GLIC vs. PIO	1	8.86 (3.18, 24.64)
GLIC vs. ROSI	1	20.51 (2.78, 151.51)
GLIM vs. LINA	1	6.08 (4.44, 8.33)
GLIM vs. LIRA	1	5.84 (3.52, 9.68)
GLIM vs. PIO	1	2.57 (0.51, 13.03)
GLIM vs. SITA	1	3.15 (2.21, 4.50)
GLIM vs. VILD	1	9.78 (6.41, 14.93)
GLIP vs. SAXA	1	76.64 (4.72, ??)
GLIP vs. SITA	1	6.49 (4.47, 9.44)
LIRA vs. SITA	2	0.61 (0.18, 2.04)
ROSI vs. SITA	1	1.08 (0.07, 17.01)
SAXA vs. SITA	1	1.14 (0.52, 2.52)
