# Supplementary material for: Comparative Efficacy and Safety of Antidiabetic Drug Regimens Added to Metformin Monotherapy in Patients with Type 2 Diabetes: A Network Meta-Analysis
Source: PLoS One. 2015 Apr 28;10(4):e0125879. doi: 10.1371/journal.pone.0125879 (PMC4412636; doi:10.1371/journal.pone.0125879)
Supplement: S6 Table — ALO = alogliptin; ALO/PIO = alogliptin/pioglitazone; CANA = canagliflozin; DAPA = dapagliflozin; EMPA = empagliflozin; EMPA/LINA = empagliflozin/linagliptin; GLIM = glimepiride; GLIP = glipizide; LINA = linagliptin; PIO = pioglitizone; PLC = placebo; SAX = saxagliptin; SITA = sitagliptin; VILDA = vildagliptin. (PDF) [file pone.0125879.s023.pdf]

Table S6. Results of Traditional Meta-Analysis Comparing Antidiabetic Therapies Effect on Experiencing Urinary Tract Infections

Comparison	No. of Trials	Relative Risk (95%CI)
ALO vs. PLC	2	1.05 (0.51, 2.15)
ALO/PIO vs. PLC	1	0.80 (0.34, 1.89)
CANA vs. PLC	2	1.38 (0.18, 10.90)
DAPA vs. PLC	1	0.96 (0.48, 1.93)
EMPA vs. PLC	2	1.20 (0.63, 2.32)
LINA vs. PLC	1	0.77 (0.32, 1.85)
SAX vs. PLC	2	1.37 (0.76, 2.46)
SITA vs. PLC	5	1.02 (0.51, 2.01)
PIO vs. PLC	1	1.25 (0.5, 3.15)
VILDA vs. PLC	2	1.11 (0.06, 19.52)
ALO vs. PIO	1	0.74 (0.34, 1.56)
ALO vs. ALO/PIO	1	1.15 (0.58, 2.27)
ALO/PIO vs. PIO	1	0.64 (0.35,1.17)
CANA vs. SITA	2	1.34 (0.71, 2.50)
CANA vs. GLIM	1	1.41 (0.88, 2.27)
DAPA vs. GLIM	1	1.77 (0.99, 3.16)
EMPA vs. EMPA/LINA	1	1.20 (0.67, 2.22)
EMPA vs. GLIM	1	0.98 (0.75, 1.27)
EMPA vs. LINA	1	0.62 (0.33, 1.14)
EMPA vs. SITA	1	1.17 (0.31, 4.41)
EMPA/LINA vs. LINA	1	0.51 (0.27, 0.98)
GLIP vs. SITA	1	0.57 (0.35, 0.57)
GLIM vs. LINA	1	1.04 (0.71, 1.52)
SAX vs. SITA	1	1.08 (0.61, 1.92)
