# Supplementary material for: Comparative Efficacy and Safety of Antidiabetic Drug Regimens Added to Metformin Monotherapy in Patients with Type 2 Diabetes: A Network Meta-Analysis
Source: PLoS One. 2015 Apr 28;10(4):e0125879. doi: 10.1371/journal.pone.0125879 (PMC4412636; doi:10.1371/journal.pone.0125879)
Supplement: S7 Table — CANA = canagliflozin; DAPA = dapagliflozin; EMPA = empagliflozin; EMPA/LINA = empagliflozin/linagliptin; GLIM = glimepiride; GLIP = glipizide; LINA = linagliptin; PLC = placebo; SITA = sitagliptin. (PDF) [file pone.0125879.s024.pdf]

Table S7. Results of Traditional Meta-Analysis Comparing Antidiabetic Therapies Effect on Experiencing Genital Tract Infections

Comparison	No. of Trials	Relative Risk (95%CI)
CANA vs. PLC	2	5.85 (1.39, 24.65)
DAPA vs. PLC	1	2.16 (0.97, 4.79)
EMPA vs. PLC	2	11.70 (1.59, 86.34)
SITA vs. PLC	3	2.27 (0.52, 9.90)
CANA vs. GLIM	1	6.04 (2.96, 12.31)
CANA vs. SITA	2	3.97 (1.71, 9.22)
DAPA vs. GLIP	1	4.57 (2.41, 8.65)
EMPA vs. EMPA/LINA	1	2.31 (1.03, 5.18)
EMPA vs. GLIM	1	5.40 (3.25, 8.98)
EMPA vs. LINA	1	8.93 (1.21, 65.96)
EMPA vs. SITA	1	1.76 (0.38, 8.27)
EMPA/LINA vs. LINA	1	3.87 (0.49, 30.61)
